# Global, regional and national burden of colorectal cancer and its risk factors, 1990–2021: a systematic analysis for the GBD 2021

**DOI:** 10.3389/fonc.2025.1673341

**Published:** 2025-11-24

**Authors:** Xuan Zeng, Jibo Wang, Ning Liu, Long Chen, Liang Liang, Li Zhuo, Xiaoyong Zhang, Mao Lin

**Affiliations:** 1 Guangdong Provincial People’s Hospital, Zhuhai Hospital (Jinwan Central Hospital of Zhuhai), Zhuhai, Guangdong, China; 2 Department of Experimental Management at Academic Affairs Office, Zunyi Medical University, Zhuhai, Guangdong, China; 3 Department of Basic Teaching and Research in General Medicine, Zunyi Medical University, Zhuhai, Guangdong, China; 4 Department of Fundamentals, Zhuhai Campus of Zunyi Medical University, Zhuhai, Guangdong, China

**Keywords:** colorectal cancer, Global Burden of Disease, Bayesian age-period-cohort model, disability-adjusted life years, age-standardized incidence rate, age-standardized mortality rate, age-standardized disability-adjusted life years rate, social demographic index

## Abstract

**Importance:**

Colorectal cancer (CRC) constitutes a significant segment of the global cancer burden, thereby warranting an in-depth epidemiological appraisal to inform strategic public health interventions and resource allocation. Previous studies, such as those based on the GBD 2019 dataset, have provided valuable insights into the CRC burden. However, they have limitations in terms of data recency, regional granularity, and comprehensive risk factor analysis.

**Objective:**

This research seeks to undertake a thorough analysis of the burden of CRC at global, regional, and national levels, along with its associated risk factors, spanning the period from 1990 to 2021. This analysis will employ data sourced from the Global Burden of Disease (GBD) 2021 study, addressing limitations in previous research by providing a more detailed and updated assessment.

**Methods:**

We assessed the distribution of CRC across 204 countries and territories, focusing on age, gender, and geographic variations. The impact of key risk factors (including behavioral risks, metabolic risks, behavioral risks, metabolic risks) on disability-adjusted life years (DALYs) was evaluated across 21 GBD regions. A Bayesian age-period-cohort (BAPC) model was employed to project CRC trends over the next three decades.

**Findings:**

In 2021, global CRC incidence was approximately 2,194,143 cases, with a prevalence of 11,679,120 and 24,401,100 DALYs. Central Europe exhibited the highest burden, with incidence peaking among individuals aged 84 to 94 years. From 1990 to 2021, age-standardized incidence, mortality, and DALY rates for CRC showed upward trends, particularly among males. The analysis of risk factors across 21 GBD regions reveals significant regional disparities in the colorectal cancer (CRC) burden, with Central Europe showing the highest contribution from risk factors (305.66). Behavioral risks, such as smoking and high alcohol use, have the greatest impact, followed by dietary risks (particularly low whole grain intake and high processed meat consumption) and metabolic risks (including high BMI and high fasting plasma glucose). By 2051, the global ASIR, ASMR, and ASDR of CRC are projected to reach 18.21 (95% UI: 10.83–25.59), 7.10 (95% UI: 4.36–9.84), and 165.21 (95% UI: 102.48–227.93) per 100,000 population, respectively, with the burden remaining higher in males than in females.

**Conclusion:**

This study provides the most granular assessment of CRC burden to date, highlighting dietary policies and sex-specific interventions as priorities. Methodological advancements in projection modeling offer actionable insights for long-term public health planning.

## 1 Introduction

Colorectal cancer (CRC), a malignancy of the gastrointestinal tract, represents a critical global health challenge. It ranks as the third most diagnosed cancer and the second leading cause of cancer-related mortality worldwide (1–3) Its clinical trajectory is often insidious, with over 60% of cases in low- and middle-income countries (LMICs) diagnosed at advanced stages. This contributing to poor survival outcomes and a disproportionate share of disability-adjusted life years (DALYs) lost to cancer (4). According to the International Agency for Research on Cancer (IARC), approximately 1.92 million new CRC cases were reported in 2022. This burden is projected to escalate by 55% by 2040 due to demographic aging and the global adoption of Westernized dietary and sedentary lifestyles (5, 6). While high-income regions have achieved progress in curbing CRC mortality through systematic screening programs (e.g., colonoscopy, fecal immunochemical testing) and advanced therapies, LMICs face a dual challenge: rising incidence rates compounded by fragmented healthcare systems and delayed diagnostic capacity (7).

The Global Burden of Disease (GBD) 2019 study provided foundational insights into CRC epidemiology, yet critical gaps hinder its application to current public health priorities. First, national-level data granularity remains insufficient, particularly in sub-Saharan Africa and South Asia. In these regions, cancer registry coverage spans less than 10% of populations, which obscures subnational disparities in risk and outcomes (8, 9). Second, Second, the absence of post-2019 data limits assessment of the COVID-19 pandemic’s impact, which precipitated a 30-50% global decline in CRC screening adherence and diagnostic delays, exacerbating preexisting inequities (10, 11). Beyond data limitations, methodological constraints in previous analyses present additional challenges. Third, reliance on deterministic projection models inadequately accounts for the complex interplay of age, period, and cohort effects. This methodological shortfall conflates biological susceptibility, temporal advancements in care, and generational shifts in risk behaviors (12, 13). For example, deterministic frameworks fail to explain the rising incidence of early-onset CRC (<50 years) in high-income nations, a trend strongly associated with obesity, processed food consumption, and sedentary lifestyles (14).

To address these limitations, this study harnesses the GBD 2021 dataset, which provides significant advantages over previous GBD cycles. While the GBD 2017 and 2019 studies provided foundational insights, they were constrained by earlier data cut-offs, lacked granularity in key regions, and relied on deterministic projection models that could not fully disentangle complex temporal trends. Our analysis, in contrast, encompassing 204 countries and territories from 1990 to 2021, specifically incorporating post-2019 data to quantitatively assess the impact of COVID-19 disruptions. Methodologically, we introduce a Bayesian age-period-cohort (BAPC) model enhanced through three innovations. First, we incorporate time-varying risk elasticities, which dynamically link temporal trends in modifiable risk factors such as smoking and processed meat intake to disease burden via GBD meta-regressions. Second, it applies penalized complexity priors, which stabilize estimates in regions with sparse data, thereby reducing the risk of overfitting. Third, it employs integrated nested Laplace approximation, a computationally efficient method for performing Bayesian inference on large datasets (15). Building upon recent methodological advances in cancer epidemiology, our framework extends the projection horizon to 2050, offering the first sex- and socio-demographic index (SDI)-stratified forecasts of CRC burden from 1990 to 2021 (16).

The implications of this analysis extend beyond academic inquiry, directly informing strategies to mitigate CRC’s growing burden. In high-risk regions such as Central Europe, where dietary factors drive 52% of CRC DALYs, fiscal policies targeting processed meats could yield substantial reductions in incidence (17). Concurrently, scaling up cost-effective screening initiatives in LMICs, modeled after Uruguay’s national program, may curb late-stage diagnoses and mortality (18). These efforts align with global health priorities, including the World Health Organization’s (WHO) mandate to reduce premature mortality from non-communicable diseases (NCDs) under Sustainable Development Goal 3.4 (19). By integrating longitudinal epidemiological data with advanced modeling techniques, this study provides a roadmap for equitable, evidence-based CRC control in an era of demographic and epidemiologic transition.

## 2 Methods

### 2.1 Overview

The study examines CRC from 1990 to 2021, utilizing data sourced from the Global Burden of Disease (GBD) 2021 study. The GBD serves as a comprehensive global health resource that quantifies the prevalence and burden of diseases, injuries, and risk factors on an international scale. For individuals unfamiliar with the GBD, it provides a comprehensive analysis of health status across various regions and periods, functioning as a vital tool for health policymakers and researchers. The dataset from the GBD 2021 encompasses a wide range of health metrics, including incidence rates, mortality rates, and disability-adjusted life years (DALYs), for 371 diseases and injuries, including CRC, across 204 countries and territories.

### 2.2 Data source

The GBD 2021 dataset provides the empirical basis for this research, offering standardized estimates of CRC incidence, mortality, DALYs, and risk factor attributions across 204 countries and territories from 1990 to 2021 (https://gbd2021.healthdata.org/gbd-results/). This dataset integrates data from 3,621 sources, including population-based cancer registries (e.g., SEER, EUROCARE), vital registration systems, hospital inpatient records, and nationally representative health surveys (e.g., WHO STEPwise, Demographic Health Surveys).

Data harmonization was conducted through a series of rigorous protocols. Underreporting was corrected using Bayesian hierarchical models, which incorporated covariates such as healthcare access (measured by the Healthcare Access and Quality Index) and GDP per capita. Age-standardization was applied to the GBD reference population to account for demographic heterogeneity. Additionally, low-quality data sources (rated<2 stars on a 5-star reliability scale) were excluded. The temporal coverage was extended to 2021, capturing the impact of the COVID-19 pandemic on CRC screening and diagnosis. Disruptions during the pandemic were modeled using interrupted time-series analysis to adjust for delayed case ascertainment. It is important to note that the GBD 2021 data used in this analysis represent the final estimates produced by IHME, which have already been adjusted for known biases and disruptions, including those related to the COVID-19 pandemic.

Estimation of CRC incidence and mortality rates was achieved using DisMod-MR 2.1, a Bayesian meta-regression tool designed to reconcile heterogeneous data sources. The model incorporated covariates such as screening coverage (colonoscopy and fecal immunochemical testing) and socio-demographic development (measured by the Socio-demographic Index, SDI) to address biases in regions with sparse data. For instance, in sub-Saharan Africa, where cancer registry coverage is limited, DisMod-MR 2.1 leveraged covariates like urbanization rates and healthcare expenditure to impute missing values. This methodological approach ensured robust estimation of CRC metrics even in data-scarce settings.

DALYs serve as a metric for quantifying the burden of CRC, incorporating both years of life lost due to premature mortality and years lived with disability (11). DALYs encompass both the years of life lost due to premature mortality and the years lived with disability, thereby offering a comprehensive assessment of disease impact. Our analysis of DALYs aimed to identify temporal trends in CRC burden throughout the study period.

The SDI, a composite measure reflecting socio-economic development through indicators such as educational attainment, per capita income, and fertility rate, was used to assess its association with the burden of CRC. The index values were categorized on a scale ranging from 0 to 1, with countries stratified into five quintiles: low, medium-low, medium, medium-high, and high. This stratification corresponded to the varying levels of developmental status among the countries under study.

### 2.3 Risk factors

The GBD 2021 study identifies 88 risk factors, specifically focusing on the DALYs attributable to CRC. The risk factors we analyzed include behavioral risks, metabolic risks, low physical activity, smoking, tobacco, dietary risks, high alcohol use, high body mass index, high fasting plasma glucose, diets low in calcium, diets low in fiber, diets low in milk, diets low in whole grains, diets high in processed meat, and diets high in red meat. These factors contribute to the DALYs associated with CRC, and our analysis measured the individual impacts of these factors within the GBD 2021 framework (20). Based on GBD 2021 on risk factors for CRC, this study investigates the effect of different risk factors on ASDR for CRC in 21 GBD regions. The attributable deaths and DALYs for each risk factor were calculated by applying the pre-computed Population Attributable Fractions (PAFs)—which were obtained directly from the GBD 2021 dataset—to the total CRC death and DALY estimates, respectively. These GBD PAFs already incorporate comprehensive uncertainty propagation from all input parameters.

### 2.4 Future projections

To project the burden of CRC through 2051, a BAPC model was implemented, with enhancements to address limitations identified in prior GBD studies (13, 21). This model disentangles temporal trends into three distinct dimensions: age effects, which reflect variations in biological susceptibility across different age groups, such as the increased incidence of CRC with advancing age; period effects, which capture temporal changes in healthcare access, screening technologies, and treatment protocols; and cohort effects, which represent generational shifts in risk behaviors, such as changes in dietary patterns or smoking initiation rates. In this study, the BAPC model was combined with integrated nested Laplace approximation (INLA) to forecast future trends in CRC burden. This advanced approach effectively untangles the complex interplay of age, period, and cohort effects on CRC incidence and mortality, providing a more precise perspective on disease trends. Utilizing Bayesian techniques, the BAPC model estimates age-specific rates while accounting for period and cohort variations, resulting in enhanced prediction accuracy.

The model incorporated several key enhancements. First, time-varying risk elasticities were operationalized by linking the model’s period effects to annual exposure levels of key risk factors (smoking and processed meat intake) from the GBD 2021 database. These exposures were incorporated as time-dependent covariates with log-linear coefficients derived from GBD meta-regressions, allowing temporal trends in risk factors to directly influence CRC incidence rates. Second, penalized complexity (PC) priors were applied to all random walk components (age, period, and cohort) to prevent overfitting. Specifically, a PC prior with a base model of a random walk of order 1 was used, with hyperparameters chosen to reflect the belief that the standard deviation of the random walk is unlikely to exceed moderate values, providing stabilization for regions with sparse data. Third, model estimation and uncertainty propagation were performed using the INLA algorithm, selected for its computational efficiency in handling high-dimensional latent Gaussian models, encompassing 204 countries over 32 years with age-stratified data. INLA provided robust posterior distributions for all parameters and a computationally feasible alternative to MCMC sampling.

The predictive performance of the BAPC model was evaluated through a back-testing procedure, in which the model was trained on data from 1990–2010 and used to predict the period 2011–2021. The model demonstrated good calibration and robust uncertainty quantification. Quantitative metrics, including MAE, RMSE, CRPS, and 95% UI coverage for each sex group, are provided in **Supplementary Table S1**, while calibration plots comparing observed and predicted values are shown in **Supplementary Figure S1**. Full details of the back-testing procedure are described in **Supplementary Method 1**.

In this framework, the BAPC linear predictor was structured as the sum of additive effects for age, period, and cohort, each modeled as a smooth random field using second-order random walks. Age effects captured biological susceptibility, period effects represented secular changes in healthcare and treatment, and cohort effects reflected generational variations in risk behavior. PC priors enforced moderate regularization, allowing the model to capture smooth trends while remaining data-driven. INLA approximation settings followed default Laplace controls, and model fit and convergence were assessed through standard measures including the deviance information criterion (DIC), Watanabe-Akaike information criterion (WAIC), and conditional predictive ordinates (CPO), ensuring reliability and stability of projections.

### 2.5 Statistical analyses

To analyze the levels in CRC incidence, deaths, and age-standardized rates (ASR) for DALYs. Specifically, the age-standardized incidence rate (ASIR), age-standardized mortality rate (ASMR), and age-standardized disability-adjusted life years rate (ASDR). This study utilized ASR metrics to evaluate levels in CRC over time. The ASR per 100,000 population was calculated using the following formula.


$$ASR=\frac{\sum_{\text{i}=1}^{A}{\text{a}}_{\text{i}}{\text{w}}_{\text{i}}}{\sum_{i=1}^{A}{\text{w}}_{\text{i}}}\times 100000$$

where a_i_ is the age-specific rate for the *i*th age group, W is the number of individuals in the standard population corresponding to the *i*th age group, and A is the total number of age groups.

Uncertainty intervals (95% UI) were generated via 1,000 Monte Carlo simulations, propagating input data variance (e.g., measurement error, model parameters).

## 3 Results

### 3.1 Global burden of CRC

**Figure 1:**
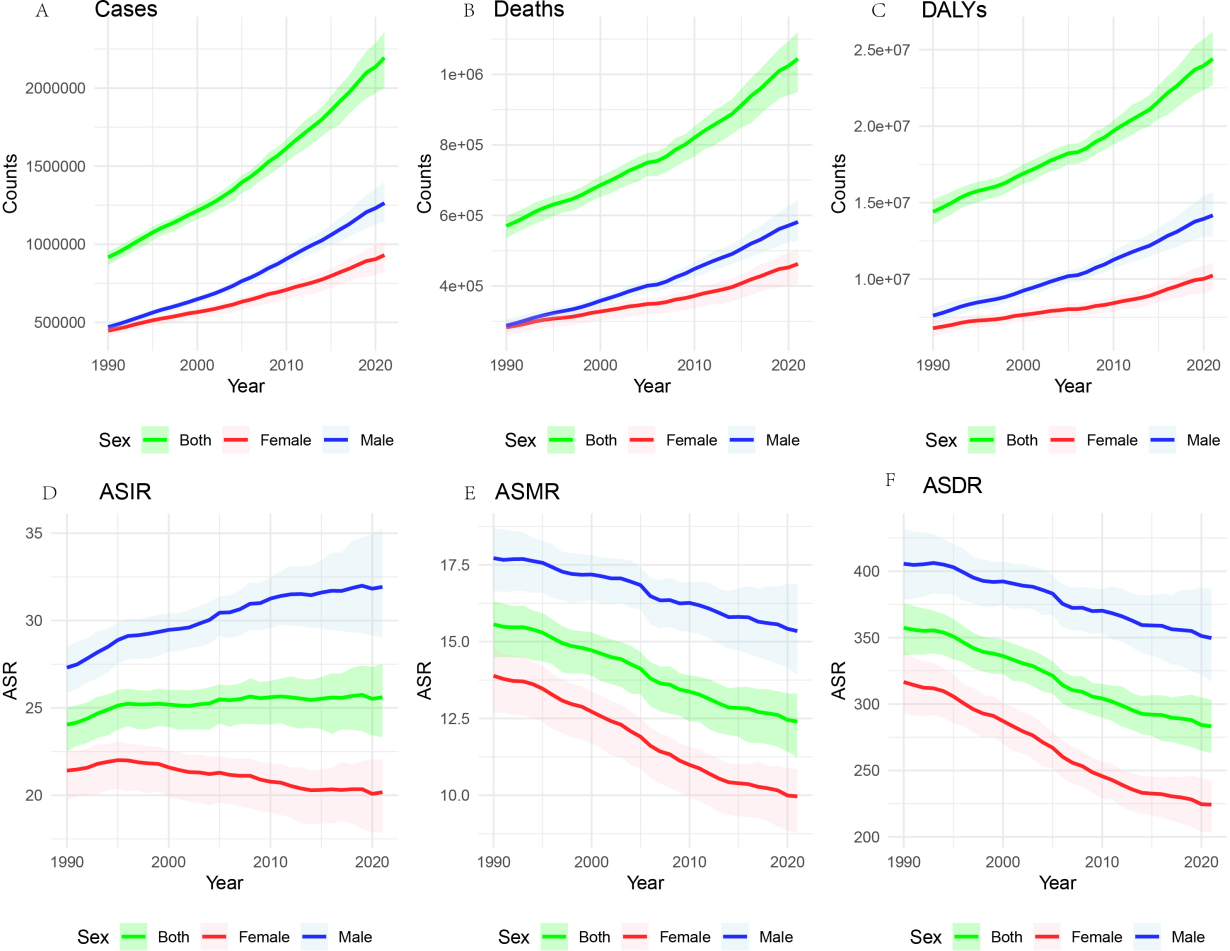
Global burden of CRC, 1990-2021. The number of cases **(A)**, the number of deaths **(B)** the total number of DALYs **(C)**. ASIR **(D)**, the age-standardized incidence rate; ASMR **(E)**, the age-standardized mortality rate; and ASDR **(F)**, the age-standardized disability-adjusted life years rate.

From 1990 to 2021, the global deaths of CRC and the number of new cases have shown a consistent annual increase, accompanied by a rise in Deaths and DALYs. Despite these escalating absolute figures, likely due to population growth, there has been a significant decline in the ASMR and ASDR (**Figure 1**).

### 3.2 CRC burden based on 21 GBD regions

**Figure 2:**
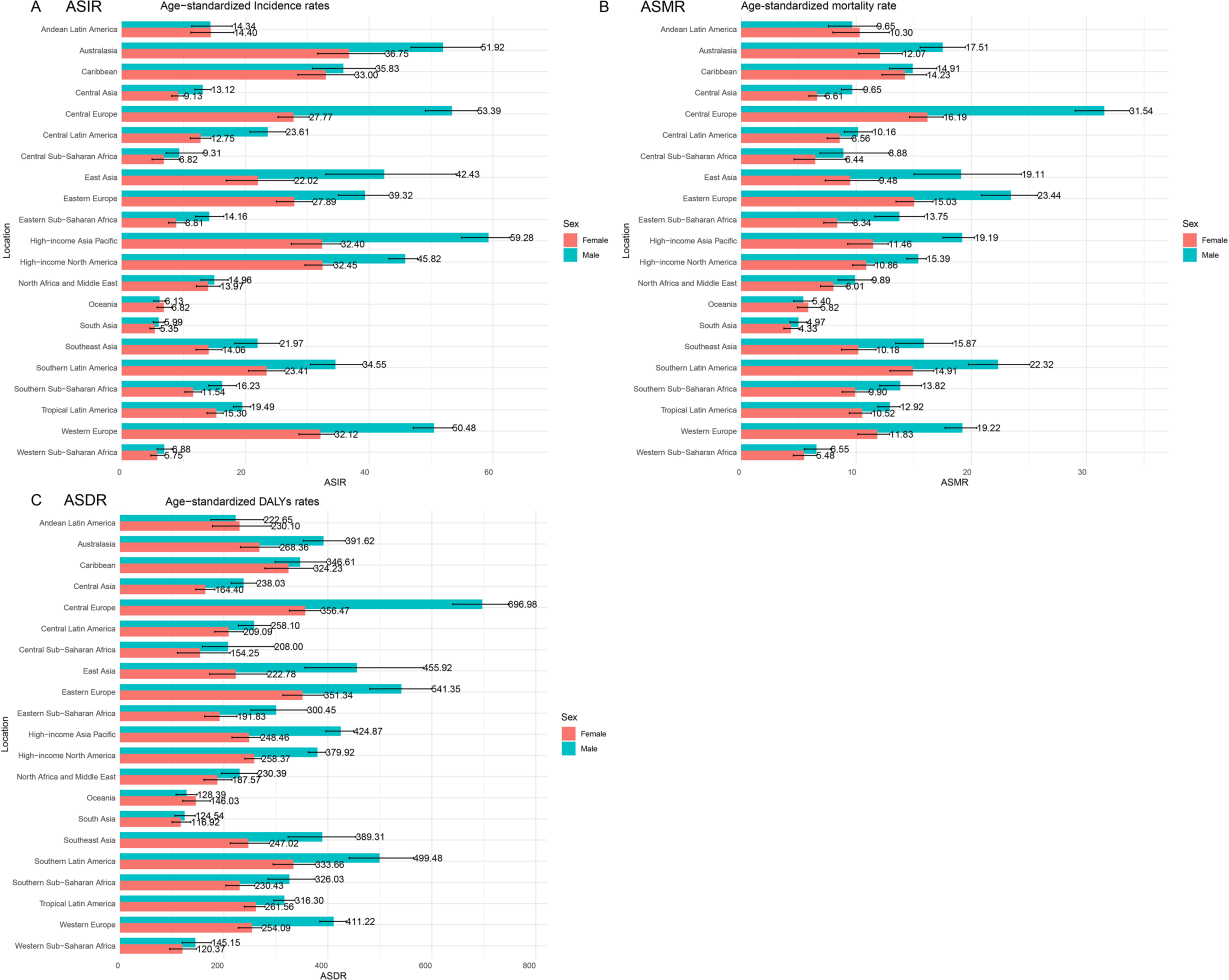
Disease burden of CRC across 21 GBD regions in 2021. **(A)** ASIR; **(B)** ASMR; **(C)** ASDR.

In 2021, the impact of CRC was evaluated across 21 GBD regions (**Figure 2**). Notable regional variations were observed. The ASIR was highest in the high-income Asia Pacific region at 44.89 per 100,000 population (95% UI: 40.2, 47.85) and lowest in South Asia at 5.65 per 100,000 population (95% UI: 5.08, 6.3). The ASMR peaked in Central Europe at 22.58 per 100,000 population (95% UI: 20.81, 24.28) and was lowest in South Asia at 4.63 per 100,000 population (95% UI: 4.17, 5.16). The ASDR showed a similar pattern, with Central Europe at 506.48 per 100,000 population (95% UI: 467.97, 544.57) and South Asia at 120.37 per 100,000 population (95% UI: 108.3, 135.3). Importantly, the burden of CRC was greater in males, especially in Central Europe, where males were nearly twice as likely to develop CRC compared to females, with an ASIR of 53.39 per 100,000 population for males versus 27.77 per 100,000 population for females.

### 3.3 Countries and territories CRC burden

In 2021, the ASIR, ASMR, and ASDR were analyzed for 204 countries and territories. The Kingdom of the Netherlands, the Principality of Monaco, and Bermuda had the highest ASIR burdens, at 69.80 (95% UI: 62.21, 76.79), 68.33 (95% UI: 54.05, 83.19), and 61.79 (95% UI: 51.46, 77.11) per 100,000 population, with EAPCs of -0.07 (95% CI: -0.30 to 0.15), 1.10 (95% CI: 0.94 to 1.26), and 0.33 (95% CI: 0.18 to 0.47), respectively.

The Eastern Republic of Uruguay, Hungary, and the Republic of Bulgaria had the highest ASMR burdens, at 27.46 (95% UI: 24.25, 30.91), 26.01 (95% UI: 21.73, 31.13), and 25.71 (95% UI: 20.96, 30.73) per 100,000 population, with EAPCs of 0.03 (95% CI: -0.06 to 0.13), -0.63 (95% CI: -0.86 to -0.40), and 1.05 (95% CI: 0.86 to 1.24), respectively.

**Figure 3:**
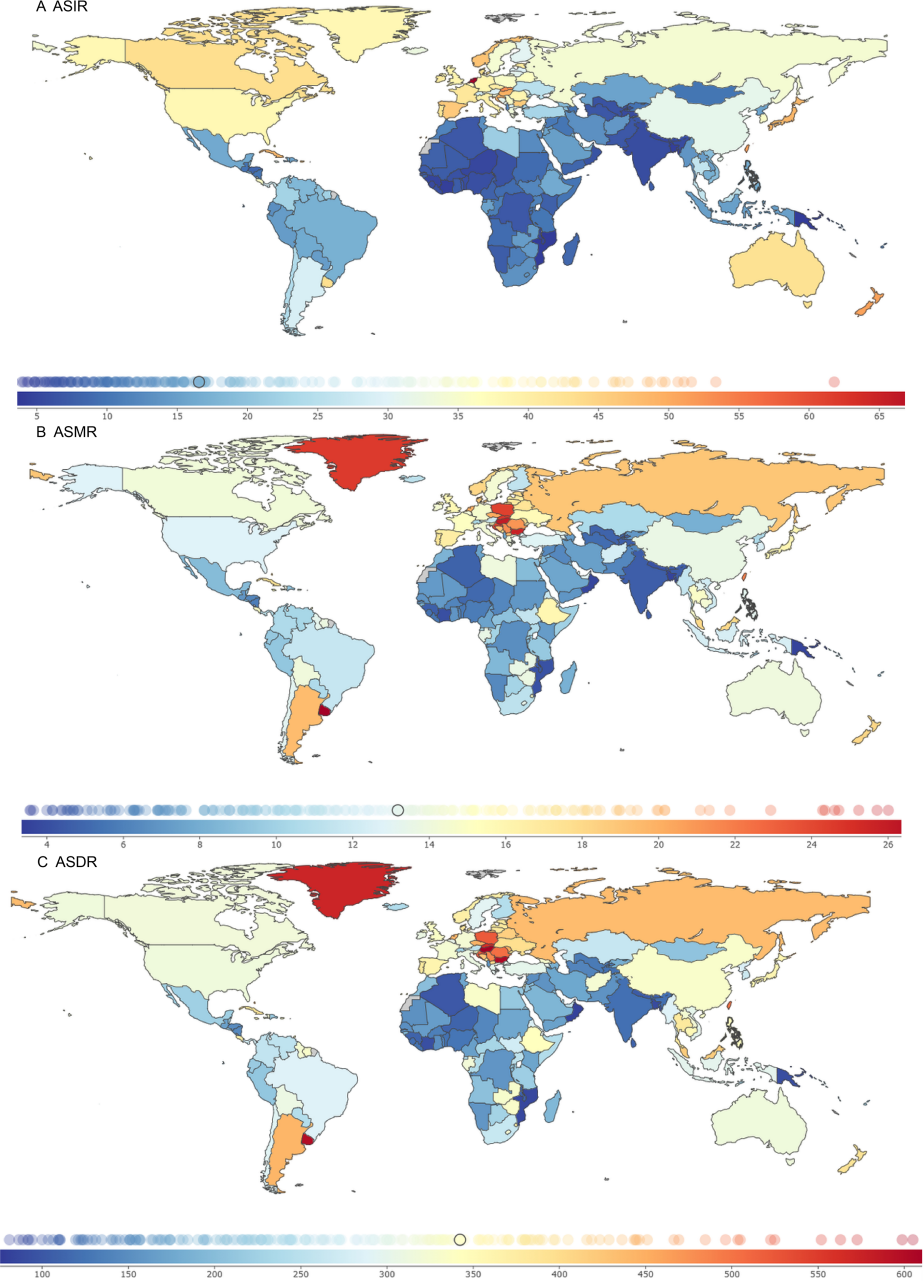
Global burden of colorectal cancer in 2021. **(A)** ASIR; **(B)** ASMR; **(C)** ASDR.

Hungary, the Republic of Bulgaria, and the Eastern Republic of Uruguay had the highest ASDR burdens, at 614.96 (95% UI: 519.37, 736.20), 605.00 (95% UI: 493.22, 726.67), and 598.78 (95% UI: 533.29, 672.27) per 100,000 population, with EAPCs of -0.53 (95% CI: -0.75 to -0.30), 0.85 (95% CI: 0.69 to 1.01), and -0.01 (95% CI: -0.11 to 0.08), respectively (**Figure 3**; **Supplementary Table S2**).

### 3.4 CRC burden by gender and SDI

**Figure 4:**
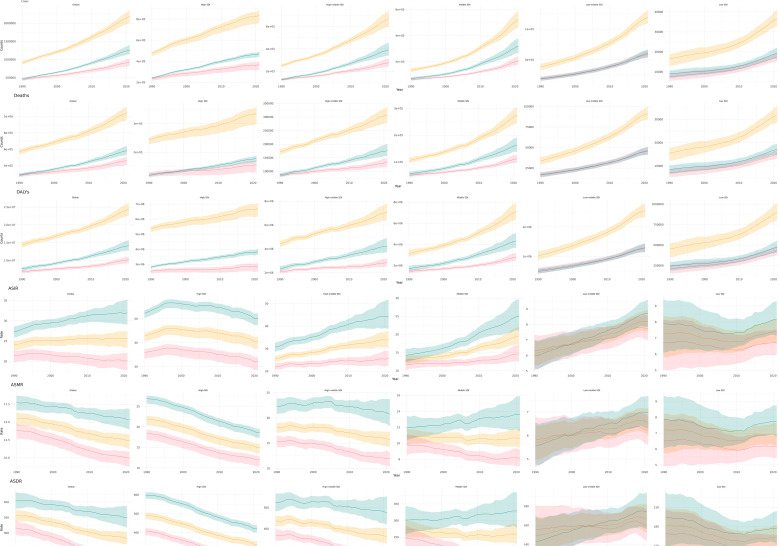
Temporal trends in the burden of CRC by gender and SDI regions from 1990 to 2021.

In all regions, the number of cases, deaths, and DALYs associated with CRC have increased annually from 1990 to 2021 (**Figure 4**). Although ASMR and ASDR are generally declining, both low and medium SDI regions are experiencing an increase in the burden of CRC. This trend indicates that SDI factors significantly influence the burden of CRC.

### 3.5 The burden of CRC by age

**Figure 5:**
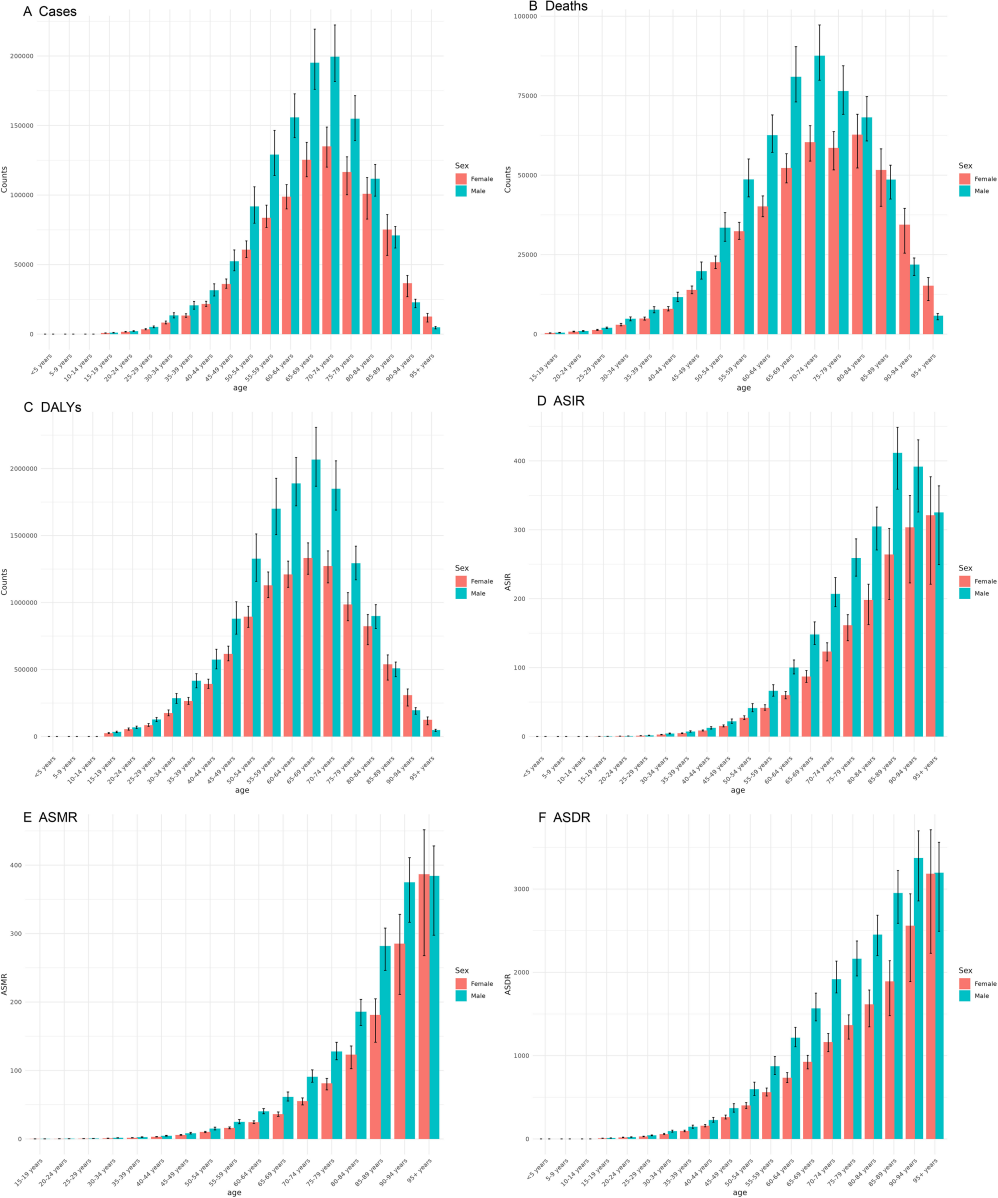
Burden of colorectal cancer among males and females across different age groups in 2021. **(A)** Number of incident cases; **(B)** Number of deaths; **(C)** DALYs; **(D)** ASIR; **(E)** ASMR; **(F)** ASDR.

The number of cases of CRC escalates with advancing age, reaching its highest between 65 and 74 years, and then gradually declines in older age groups (**Figure 5**). ASIR exhibits a continuous rise as age increases, reaching its highest point between 85 and 94 years. These findings indicate that the burden of CRC intensifies with age. The number of deaths and DALYs initially rises and then decreases, with their peaks occurring at ages 65–79 years and 60–74 years, respectively. This level is reflected in the ASMR and ASDR, both of which show a similar level of increasing with age.

### 3.6 Risk factors contributing to CRC burden

**Figure 6:**
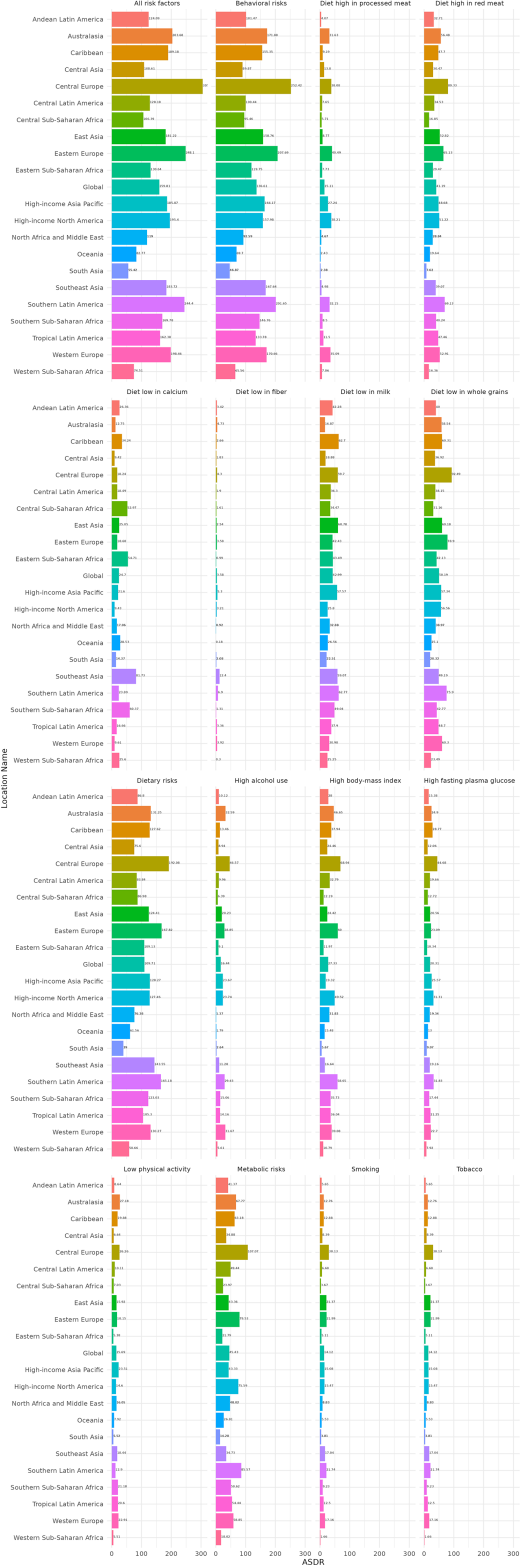
ASDR for CRC for 21 GBD regions risk factors in 2021.

The analysis of risk factors contributing to the CRC burden across the 21 GBD regions reveals significant regional disparities, with distinct contributions from specific risk factors. Central Europe exhibits the highest total contribution from risk factors to the CRC burden, amounting to 305.66 per 100,000 population. Within this region, dietary risks account for an ASDR of 192.08 per 100,000 population, while behavioral risks contribute an ASDR of 252.42 per 100,000 population. Notably, the impact of specific risk factors varies markedly across different GBD regions. For instance, in the Andean Latin America region, low milk intake (Diet low in milk) results in a CRC ASDR of 42.28 per 100,000 population, whereas smoking contributes a relatively lower ASDR of 5.65 per 100,000. In contrast, in the Southeast Asia region, low calcium intake (a Diet low in calcium) leads to a CRC ASDR of 81.73 per 100,000, while high alcohol use contributes a significantly lower ASDR of 11.28 per 100,000. Overall, among the risk factors contributing to the CRC burden, behavioral risks have the most substantial impact, followed by dietary risks, and then metabolic risks. Within dietary risks, low fiber intake (Diet low in fiber) generally contributes less to the CRC burden compared to low whole grain intake (Diet low in whole grains), which has the highest contribution. For example, in Central Europe, smoking results in a CRC ASDR of 8.39 per 100,000, and high alcohol use contributes an ASDR of 8.94 per 100,000. In Eastern Europe, the ASDR attributable to smoking is 21.37 per 100,000, and that for high alcohol use is 28.85 per 100,000. Regarding metabolic risks, the contributions of high BMI and high fasting plasma glucose to the CRC burden also vary across regions. In high-income North America, high BMI accounts for a CRC ASDR of 49.52 per 100,000, and high fasting plasma glucose contributes an ASDR of 31.31 per 100,000 (**Figure 6**; **Supplementary Table S3**).

### 3.7 Projections of CRC burden over the next 30 years

**Figure 7:**
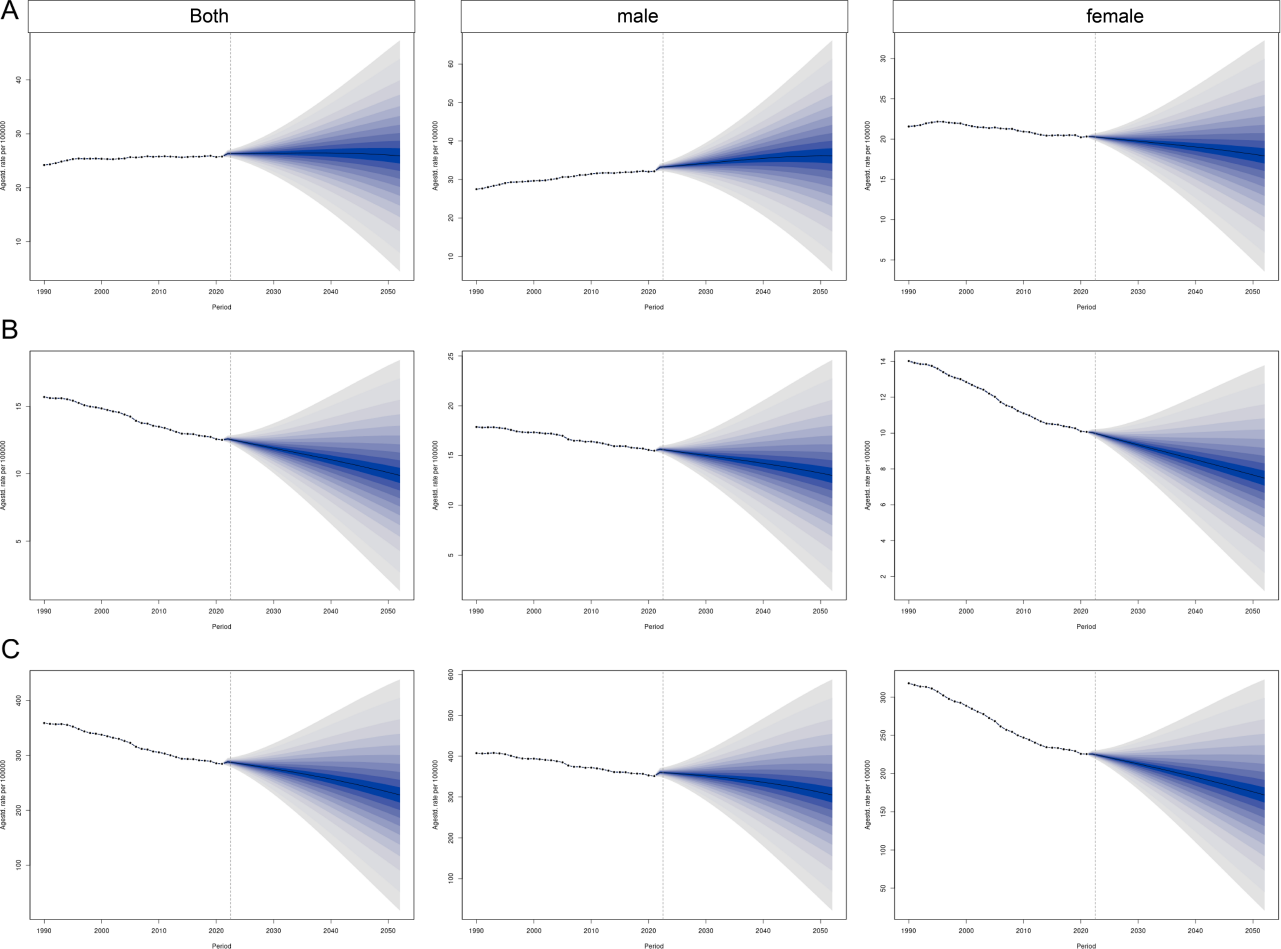
Prediction of the global burden of CRC over the next 30 years based on the Bayesian age–period–cohort model. **(A)** ASIR; **(B)** ASMR; **(C)** ASDR.

By 2051, the global burden of CRC is projected to decline substantially. The predicted ASIRs for both sexes, males, and females are 18.21 (95% UI: 10.83, 25.59), 25.36 (95% UI: 15.11, 35.61), and 12.51 (95% UI: 7.28, 17.75) per 100,000 population, respectively. The corresponding ASMRs are 7.10 (95% UI: 4.36, 9.84), 9.40 (95% UI: 5.87, 12.94), and 5.34 (95% UI: 3.22, 7.46) per 100,000 population. The ASDRs are 165.21 (95% UI: 102.48, 227.93), 223.53 (95% UI: 142.96, 304.10), and 124.21 (95% UI: 76.84, 171.58) per 100,000 population, respectively (**Figure 7**).

## 4 Discussion

This study provides a comprehensive analysis of trends in the global burden of CRC, emphasizing gender disparities and the impact of various risk factors. Although there has been an annual increase in the incidence and mortality rates of CRC, the ASIR, ASMR, and ASDR have shown a decline. This trend is primarily attributed to population growth and a reduction in the overall disease burden (7). In developed countries, CRC incidence and ASMR have stabilized or decreased, attributed largely to advancements in CRC screening programs and the widespread availability of colonoscopy procedures (22, 23).

In developed countries, there has been a stabilization or decrease in the number of new cases and ASMR, largely due to advancements in CRC screening programs and the widespread availability of colonoscopy procedures. However, it is essential to consider regional disparities. For instance, the highest ASIR has been observed in the high-income Asia-Pacific region, contrasting with the lowest rates found in South Asia. These variations are likely attributable to a confluence of factors, including economic development levels, healthcare resource accessibility, population lifestyle, and environmental influences (24, 25).

It is important to note that the burden estimates presented, particularly for regions with historically limited cancer registration, are the product of the GBD’s robust modeling framework which is explicitly designed to address data incompleteness. The wider uncertainty intervals around estimates for regions such as Sub-Saharan Africa quantitatively reflect the greater uncertainty inherent in the underlying data. Consequently, our comparative analyses of risk factors and trends across regions are based on these adjusted estimates, which provide a more valid basis for cross-country comparison than raw, unadjusted registry data would allow.

Research strongly confirms that modifiable lifestyle factors, particularly dietary patterns, alcohol consumption, and smoking, significantly contribute to the CRC burden (15). Specifically, a diet characterized by high intake of red and processed meats and low in fiber is consistently identified as a major risk factor (16). Regarding alcohol, even minimal intake has been shown to significantly elevate CRC risk, a finding that applies regardless of the beverage type (26). Furthermore, cigarette smoking stands as another well-established, independent risk factor for the disease (26).

The hierarchy of CRC risk factors demonstrates substantial geographic heterogeneity. The global predominance of behavioral risks reflects their widespread population impact, whereas dietary drivers emerge as the primary modifiable factor in specific high-burden regions such as Central Europe. Notably, extreme regional risks like low milk intake in Andean Latin America and low calcium intake in Southeast Asia highlight the critical influence of local dietary patterns and baseline nutritional status. These findings are biologically plausible, as calcium and dairy intake have demonstrated protective effects against CRC, and their attribution is magnified in populations with chronically low consumption.

Gender disparities in the burden of CRC are notable, with males experiencing a greater incidence than females. This discrepancy may be attributed to gender-specific biological factors, such as hormone levels, as well as lifestyle factors, including smoking and other unhealthy behaviors (17). For instance, Jemal et al. demonstrated higher morbidity and mortality rates of CRC in males compared to females (18). Potential explanations for this gender disparity include variations in hormonal profiles, the prevalence of smoking, and differences in dietary practices (27). Beyond modifiable risks, hereditary factors also contribute to CRC burden. Lynch syndrome, the most prevalent hereditary form, accounts for approximately 3% of all CRC cases (28). The combined variation in hereditary predisposition, modifiable risk factors, and gender across different regions further intensifies disparities in the global burden of CRC.

In addition to lifestyle and hereditary factors, emerging biological mechanisms may contribute to the observed sex disparity. Estrogen is thought to confer a protective effect in females via anti-proliferative pathways, while the male gut microbiome exhibits a higher abundance of bacteria capable of converting primary bile acids into potentially genotoxic secondary bile acids. The interplay between sex-hormone-mediated immunity and a sexually dimorphic gut microbiome represents a promising area for elucidating the etiological basis of this disparity (17, 29).

Societal development has increased consumption of animal-based foods and sedentary behavior, elevating CRC risk through obesity (30), while adequate intake of calcium, whole grains, and fiber may reduce risk (31). The variation in both hereditary predisposition and these modifiable factors across regions and genders intensifies disparities in CRC burden, underscoring the importance of comprehensive primary prevention strategies (32). Priority actions include taxing processed meats in high SDI regions (e.g., Europe) and scaling up colonoscopy access in low SDI regions (e.g., Sub-Saharan Africa), leveraging successful models from Uruguay’s national screening program. Our findings on the stratified burden can inform the design of context-specific strategies. For high SDI regions, key interventions might include comprehensive screening programs and fiscal policies. In middle SDI regions, a focus on opportunistic screening and diagnostic capacity is supported. For low SDI regions, the results underscore the importance of foundational health information systems and primary prevention.

By applying a BAPC model, our study advances beyond previous deterministic approaches to forecast a gradual decline in the CRC burden over the next three decades. This methodology provides novel, sex-stratified insights, refines the understanding of early-onset CRC trends, and incorporates adjustments for recent disruptions such as the COVID-19 pandemic, thereby offering critical guidance for long-term public health planning (33). Effective strategies to mitigate the incidence of CRC include advocating for a diet high in fiber and calcium, enhancing physical activity, reducing tobacco and alcohol consumption, and limiting the intake of red and processed meats (32). Furthermore, the burden of CRC can be further alleviated by directing health education efforts toward high-risk groups, taking into account regional variations in CRC risk factors. CRC screening, especially through colonoscopy, has demonstrated efficacy in decreasing CRC mortality and morbidity (34). By emulating successful screening frameworks from developed nations and tailoring them to align with local healthcare resources, countries can substantially alleviate the burden of CRC. The decline in ASMR observed in high SDI regions, in contrast to the stable or rising trends in many low SDI and middle SDI regions, can be largely attributed to the widespread implementation of organized screening programs. While our study design does not allow for a direct quantitative linkage, extensive evidence from the literature quantifies this impact. For instance, modeling studies and long-term observational data have demonstrated that organized screening programs, particularly with colonoscopy or fecal immunochemical test (FIT), are associated with a reduction in CRC mortality by approximately 40% in the target populations (35, 36). The temporal alignment between the rollout of national screening initiatives in countries like the United States and Germany and the subsequent accelerated decline in their CRC mortality rates provides strong ecological support for this causal relationship (37, 38). Therefore, the disparities in ASMR trends observed in our results are consistent with, and likely causally influenced by, the stark global inequity in access to effective CRC screening. Scaling up screening capacity in transitioning economies represents the single most promising strategy for bridging this mortality gap. Nonetheless, the precision of these models is influenced by various factors, such as the data source and sample size, necessitating a comprehensive consideration of these uncertainties in the formulation of policy (39).

Although explicit counterfactual policy modeling was beyond the scope of this study, the stratified burden estimates and projections presented here provide useful evidence to support the prioritization of colorectal cancer prevention strategies. For example, regions with high dietary-attributable CRC burdens may benefit from fiscal or labeling policies targeting processed meat consumption, while low SDI and middle SDI regions experiencing rising incidence could prioritize scaling up FIT-based screening programs. These findings offer practical guidance for tailoring interventions to local epidemiological profiles.

Although the uncertainty intervals for risk-attributable burden account for parameter uncertainty, dedicated analyses exploring the interdependence of risk factors (e.g., leave-one-risk-out sensitivity analysis) represent a valuable avenue for future methodological research. Our study is also limited by the lack of data on hereditary CRC syndromes and molecular subtypes, as the GBD relies on aggregate registry data. This precludes an assessment of how genetic factors contribute to the burden, especially in high-risk populations. Future integration of genomic epidemiology is essential to bridge this gap and develop subtype-specific burden estimates. Future studies should integrate multi-omics data with population-level analyses to clarify the mechanisms behind CRC disparities, especially in early-onset cases. Concurrently, robust systems for monitoring early-onset CRC trends are needed to guide targeted prevention. Although our study offers comprehensive insights into trends concerning the burden of CRC, including gender and regional disparities and the influence of risk factors, it is not devoid of limitations. Despite utilizing broad and representative data sources, potential biases and uncertainties may persist, highlighting the need for further enhancements and refinements in subsequent research. Projections assume no disruptive technological breakthroughs (e.g., liquid biopsy adoption); thus, results represent conservative estimates requiring periodic recalibration.

## 5 Conclusion

This research provides a detailed overview of the incidence and related risk factors of CRC from 1990 to 2021, using data from GBD 2021. Notable regional variations in CRC burden and dietary risks are identified. In particular, Central Europe is facing the highest burden, with dietary risks identified as the primary contributor to this burden. The study emphasizes the importance of tackling modifiable risk factors through public health programs, as these factors greatly impact CRC incidence and mortality. The anticipated reduction in CRC burden over the next thirty years, especially among females, presents an encouraging perspective, indicating the potential success of current health initiatives. Future research should focus on refining these projections and exploring the underlying reasons for regional disparities to facilitate the development of targeted prevention strategies.

## Data Availability

The datasets presented in this study can be found in online repositories. The names of the repository/repositories and accession number(s) can be found in the article/**Supplementary Material**.
