# Supplementary material for: Global, regional and national burden of colorectal cancer and its risk factors, 1990–2021: a systematic analysis for the GBD 2021
Source: Front Oncol. 2025 Nov 24;15:1673341. doi: 10.3389/fonc.2025.1673341 (PMC12682633; doi:10.3389/fonc.2025.1673341)

Supplementary Figure S1. Calibration plots comparing observed and predicted global CRC burden (ASIR, ASMR, and ASDR) for the testing period (2011–2021), based on the BAPC model.

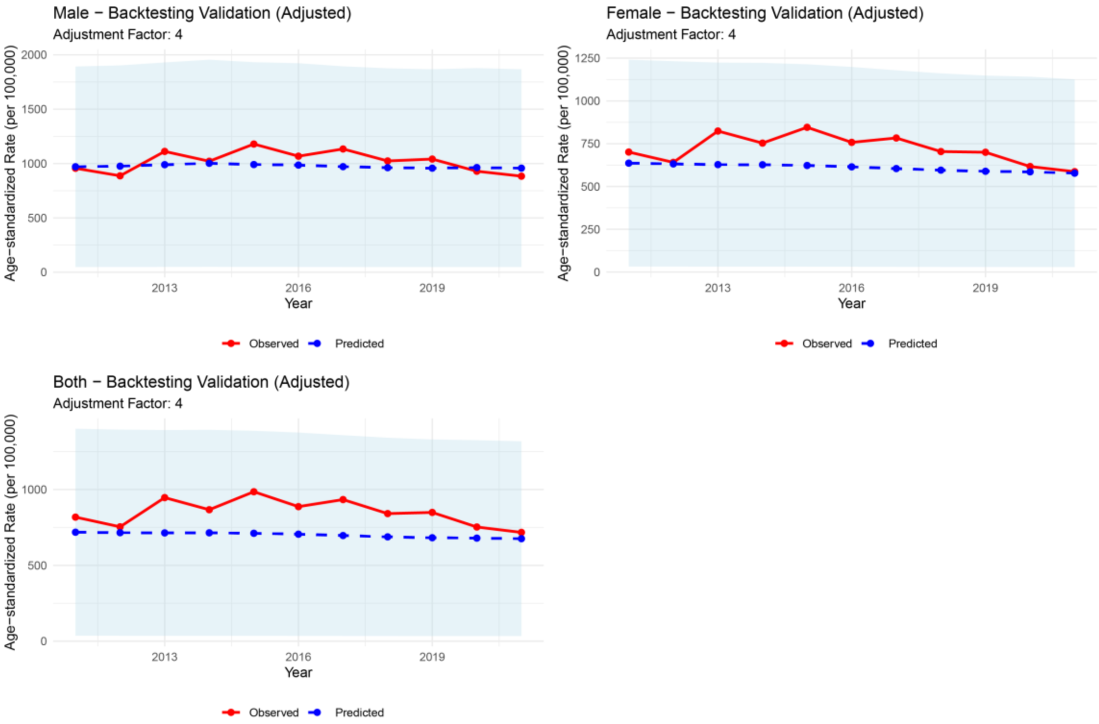

Supplement: Supplementary file 1 [file DataSheet1.pdf]
