# Supplementary material for: Global, regional and national burden of colorectal cancer and its risk factors, 1990–2021: a systematic analysis for the GBD 2021
Source: Front Oncol. 2025 Nov 24;15:1673341. doi: 10.3389/fonc.2025.1673341 (PMC12682633; doi:10.3389/fonc.2025.1673341)
Supplement: Supplementary file 2 [file DataSheet2.pdf]

Supplementary Method 1 Back-testing Validation

A back-testing procedure was implemented to validate the predictive performance of the Bayesian age-period-cohort (BAPC) model. The available data series from 1990 to 2021 was partitioned into a training period (1990-2010) and a testing period (2011-2021). The model was fitted exclusively on the training data and then used to generate forecasts for the testing period.

Model performance was evaluated using several quantitative metrics calculated from the comparison between predicted and observed values. These included: Mean Absolute Error (MAE), measuring the average magnitude of prediction errors; Root Mean Square Error (RMSE), calculating the square root of average squared errors; Continuous Ranked Probability Score (CRPS), assessing the accuracy of the full predictive distribution; and 95% Uncertainty Interval (UI) Coverage, determining the percentage of observations falling within the 95% predictive intervals. All metrics were computed globally and stratified by sex (Both sexes, Male, Female).

The complete results of this back-testing analysis are provided in Supplementary Table S1.
