# Supplementary material for: Global, regional and national burden of colorectal cancer and its risk factors, 1990–2021: a systematic analysis for the GBD 2021
Source: Front Oncol. 2025 Nov 24;15:1673341. doi: 10.3389/fonc.2025.1673341 (PMC12682633; doi:10.3389/fonc.2025.1673341)
Supplement: Supplementary file 4 [file Table1.docx]

eTable1. Incidence of CRC between 1990 and 2021at the Global and Regional Level

| Location | Rate per 100,000 (95% UI) | | | | | | | |
| --- | --- | --- | --- | --- | --- | --- | --- | --- |
|  | 1990 |  |  | 2021 |  |  | 1990-2021 |  |
|  | Incidence cases | ASIR |  | Incidence cases | ASIR |  | Cases change | EAPC (95% CI) |
| Global | 916583.53 (866238.31,951894.97) | 24.04  (22.54,25.01) |  | 2194143.25 (2001271.82,2359390.09) | 25.61 (23.32,27.52) |  | 2.84  (2.81,2.87) | 0.15  (0.12,0.19) |
| Sex | | | | | | | | |
| Male | 469762.64 (445308.28,492302.50) | 27.31  (25.89,28.51) |  | 1263462.39 (1146499.47,1400377.23) | 31.93 (29.04,35.26) |  | 3.27  (3.23,3.30) | 0.50  (0.45,0.54) |
| Female | 446820.89 (414136.02,472552.62) | 21.41  (19.75,22.62) |  | 930680.86 (824673.72,1017652.43) | 20.17 (17.86,22.05) |  | 2.33  (2.29,2.37) | -0.29  (-0.34,-0.25) |
| Regions | | | | | | | | |
| Andean Latin America | 1902.38 (1647.69,2186.12) | 9.49  (8.24,10.87) |  | 8452.06 (6698.58,10526.05) | 14.39 (11.39,17.89) |  | 5.14  (5.02,5.26) | 1.43  (1.32,1.53) |
| Australasia | 11843.25 (10910.28,12803.79) | 50.58 (46.64,54.65) |  | 23280.74 (20501.57,26412.69) | 43.97 (38.91,49.55) |  | 2.06  (1.93,2.18) | -0.58  (-0.71,-0.44) |
| Caribbean | 6206.59 (5824.85,6606.77) | 24.09 (22.57,25.67) |  | 18481.22 (16074.15,20935.80) | 34.33 (29.87,38.86) |  | 3.71  (3.62,3.81) | 1.26  (1.19,1.34) |
| Central Asia | 6180.16 (5774.01,6592.19) | 12.90 (12.03,13.79) |  | 8892.97 (7941.56,9809.25) | 10.82 (9.69,11.90) |  | 1.50  (1.19,1.81) | -0.15  (-0.36,0.06) |
| Central Europe | 42181.94 (40229.86,43855.90) | 28.32 (27.03,29.45) |  | 85866.58 (79290.33,92786.95) | 38.82 (35.71,41.96) |  | 2.34  (2.18,2.51) | 0.98  (0.81,1.15) |
| Central Latin America | 7632.33 (7310.61,7895.73) | 9.32  (8.90,9.67) |  | 44551.54 (39665.47,49720.64) | 17.74 (15.75,19.81) |  | 5.87  (5.81,5.93) | 2.05  (1.99,2.11) |
| Central Sub-Saharan  Africa | 1590.92 (1268.40,1963.74) | 7.40  (6.06,9.00) |  | 4206.45 (3206.03,5573.58) | 7.87  (6.09,10.53) |  | 3.20  (2.93,3.47) | 0.24  (0.06,0.41) |
| East Asia | 165083.41 (142141.86,189658.31) | 19.08 (16.55,21.79) |  | 684927.15 (559522.76,823301.33) | 31.60 (25.90,37.85) |  | 4.85  (4.70,5.00) | 1.75  (1.65,1.84) |
| Eastern Europe | 71750.52 (69017.26,73942.57) | 25.52 (24.50,26.32) |  | 113252.18 (104414.53,122488.52) | 32.11 (29.59,34.73) |  | 1.28  (1.15,1.42) | 0.62  (0.51,0.74) |
| Eastern Sub-Saharan  Africa | 8179.36 (6322.72,9307.75) | 11.08  (8.75,12.51) |  | 17951.90 (15710.43,20779.07) | 11.23 (9.84,12.77) |  | 2.41  (2.18,2.64) | -0.10  (-0.22,0.01) |
| High-income  Asia Pacific | 79543.27 (75502.03,82394.44) | 39.72 (37.43,41.17) |  | 207276.97 (179498.14,223331.93) | 44.89 (40.20,47.85) |  | 3.09  (2.93,3.25) | 0.33  (0.23,0.44) |
| High-income  North America | 167645.17 (155818.40,174737.06) | 47.34 (44.19,49.26) |  | 244681.45 (226550.01,256376.91) | 38.75 (36.13,40.48) |  | 1.11  (1.02,1.20) | -0.80  (-0.93,-0.67) |
| North Africa and  Middle East | 17568.09 (14962.62,19789.76) | 10.40  (9.08,11.63) |  | 66086.55 (58129.63,74936.33) | 14.43 (12.67,16.35) |  | 4.62  (4.42,4.81) | 1.31  (1.15,1.48) |
| Oceania | 196.96  (161.74,238.77) | 6.82  (5.75,8.03) |  | 481.60  (409.98,560.14) | 6.44  (5.55,7.42) |  | 2.94  (2.80,3.08) | -0.18  (-0.27,-0.09) |
| South Asia | 28138.21 (24015.65,31933.64) | 4.69  (4.00,5.34) |  | 85115.13 (76613.73,95247.80) | 5.65  (5.08,6.30) |  | 3.56  (3.40,3.71) | 0.46  (0.33,0.60) |
| Southeast Asia | 29320.90 (24899.61,33179.32) | 11.28  (9.64,12.72) |  | 116941.67 (101259.79,132256.46) | 17.70 (15.41,19.89) |  | 4.53  (4.50,4.55) | 1.45  (1.40,1.50) |
| Southern  Latin America | 10982.50 (10129.59,11892.01) | 24.09 (22.12,26.13) |  | 24746.82 (21921.77,27561.45) | 28.32 (25.10,31.53) |  | 2.86  (2.68,3.04) | 0.73  (0.56,0.90) |
| Southern Sub-Saharan  Africa | 2516.31 (2233.99,3056.70) | 9.46  (8.37,11.65) |  | 7623.41 (6875.23,8472.18) | 13.45 (12.16,14.83) |  | 3.74  (3.50,3.97) | 1.29  (1.06,1.52) |
| Tropical  Latin America | 9843.20 (9351.15,10323.58) | 11.00 (10.32,11.56) |  | 44244.63 (40859.43,47143.63) | 17.17 (15.82,18.31) |  | 4.97  (4.83,5.11) | 1.42  (1.31,1.54) |
| Western Europe | 243931.40 (229598.29,253377.82) | 41.81 (39.51,43.40) |  | 375461.84 (337713.28,401850.24) | 40.54 (37.17,43.07) |  | 1.44  (1.30,1.58) | -0.11  (-0.26,0.04) |
| Western Sub-Saharan  Africa | 4346.67 (3709.09,5101.10) | 5.20  (4.44,6.04) |  | 11620.39 (9673.14,13673.40) | 6.29  (5.36,7.30) |  | 3.37  (3.26,3.48) | 0.78  (0.71,0.85) |

Abbreviations: ASIR, age-standardized incidence rate; EAPC, estimated annual percentage change; SDI, Sociodemographic Index; UI, uncertainty interval.

eTable2. Death of CRC between 1990 and 2021 at the Global and Regional Level

| Location | Rate per 100,000 (95% UI) | | | | | | | |
| --- | --- | --- | --- | --- | --- | --- | --- | --- |
|  | 1990 |  |  | 2021 |  |  | 1990-2021 |  |
|  | Death cases | ASMR |  | Death cases | ASMR |  | Cases change | EAPC (95% CI) |
| Global | 570318.55 (536544.84,597668.66) | 15.56  (14.49,16.31) |  | 1044072.21 (950187.61,1120169.33) | 12.40 (11.24,13.31) |  | 1.93  (1.88,1.98) | -0.81  (-0.84,-0.77) |
| Sex | | | | | | | | |
| Male | 287710.67 (269819.10,304982.31) | 17.72  (16.67,18.68) |  | 581557.26 (528252.82,641420.11) | 15.35 (13.94,16.87) |  | 2.29  (2.26,2.33) | -0.50  (-0.52,-0.47) |
| Female | 282607.87 (258885.98,301416.10) | 13.89  (12.68,14.80) |  | 462514.95 (407296.33,503539.24) | 9.96  (8.78,10.84) |  | 1.51  (1.45,1.58) | -1.19  (-1.24,-1.14) |
| Regions | | | | | | | | |
| Andean Latin America | 1722.56 (1495.50,1967.13) | 8.88  (7.71,10.08) |  | 5777.84 (4597.21,7047.45) | 10.00 (7.98,12.18) |  | 4.23  (4.11,4.36) | 0.46  (0.35,0.57) |
| Australasia | 5777.75 (5304.53,6246.04) | 24.88  (22.77,26.91) |  | 8275.77 (7178.89,9408.21) | 14.62 (12.77,16.52) |  | 0.93  (0.83,1.03) | -1.92  (-2.01,-1.82) |
| Caribbean | 3471.99 (3238.58,3720.89) | 13.96 (13.01,14.95) |  | 7881.54 (6866.69,8970.24) | 14.57 (12.72,16.60) |  | 2.82  (2.77,2.87) | 0.27  (0.23,0.31) |
| Central Asia | 4831.31 (4498.76,5156.83) | 10.35  (9.61,11.06) |  | 6146.25 (5492.55,6771.90) | 7.87  (7.06,8.65) |  | 1.08  (0.85,1.30) | -0.50  (-0.65,-0.34) |
| Central Europe | 32120.12 (30603.11,33395.98) | 22.04 (21.00,22.93) |  | 51843.49 (47752.16,55681.29) | 22.58 (20.81,24.28) |  | 1.56  (1.46,1.66) | -0.03  (-0.15,0.10) |
| Central Latin America | 5615.77 (5367.62,5819.03) | 7.21  (6.85,7.49) |  | 22934.27 (20341.66,25521.14) | 9.30  (8.26,10.35) |  | 4.75  (4.69,4.82) | 0.86  (0.78,0.94) |
| Central Sub-Saharan  Africa | 1493.97 (1200.08,1837.78) | 7.43  (6.05,9.04) |  | 3686.77 (2802.24,4932.00) | 7.45  (5.77,10.14) |  | 2.98  (2.75,3.20) | 0.06  (-0.09,0.21) |
| East Asia | 123638.04 (106930.29,141856.96) | 15.43 (13.39,17.61) |  | 287880.01 (235559.37,343280.49) | 13.78 (11.33,16.35) |  | 2.72  (2.63,2.81) | -0.44  (-0.50,-0.39) |
| Eastern Europe | 50096.80 (48178.39,51687.69) | 18.06 (17.33,18.65) |  | 64373.25 (59115.50,69822.45) | 18.05 (16.58,19.56) |  | 0.56  (0.45,0.68) | -0.19  (-0.31,-0.07) |
| Eastern Sub-Saharan  Africa | 7778.77 (5997.52,8844.28) | 11.06  (8.71,12.49) |  | 15967.48 (13939.89,18320.33) | 10.76 (9.41,12.15) |  | 2.22  (2.01,2.42) | -0.21  (-0.31,-0.12) |
| High-income  Asia Pacific | 35635.73 (33642.83,36953.94) | 18.45  (17.23,19.20) |  | 80690.59 (67266.65,88001.37) | 14.99 (13.03,16.09) |  | 2.64  (2.58,2.71) | -0.73  (-0.78,-0.69) |
| High-income  North America | 73963.93 (67846.12,77459.92) | 20.58 (18.95,21.52) |  | 85865.38 (77871.23,90928.14) | 12.95 (11.89,13.65) |  | 0.36  (0.30,0.42) | -1.63  (-1.69,-1.57) |
| North Africa and  Middle East | 14108.39 (12125.66,15839.38) | 8.99  (7.94,10.06) |  | 37394.51 (32758.79,42274.13) | 8.95  (7.85,10.08) |  | 3.43  (3.25,3.61) | 0.21  (0.06,0.36) |
| Oceania | 165.31  (136.17,200.22) | 6.31  (5.37,7.42) |  | 381.66  (324.23,445.96) | 5.58  (4.80,6.42) |  | 2.77  (2.62,2.92) | -0.37  (-0.47,-0.26) |
| South Asia | 25281.24 (21597.24,28740.57) | 4.42  (3.76,5.06) |  | 66942.71 (60196.67,74844.40) | 4.63  (4.17,5.16) |  | 3.13  (3.01,3.25) | 0.02  (-0.09,0.13) |
| Southeast Asia | 24777.24 (20969.45,28065.26) | 10.11  (8.66,11.38) |  | 79419.60 (68449.69,89290.46) | 12.74 (11.12,14.26) |  | 3.79  (3.75,3.83) | 0.74  (0.67,0.80) |
| Southern  Latin America | 8973.71 (8273.58,9706.28) | 20.13 (18.48,21.81) |  | 16117.02 (14307.19,18001.80) | 18.13 (16.12,20.25) |  | 2.15  (1.96,2.33) | -0.10  (-0.27,0.07) |
| Southern Sub-Saharan  Africa | 2225.26 (1974.24,2720.01) | 8.82  (7.79,10.94) |  | 6129.62 (5550.19,6785.91) | 11.47  (10.37,12.61) |  | 3.40  (3.12,3.68) | 0.97  (0.70,1.25) |
| Tropical  Latin America | 8102.19 (7660.32,8506.31) | 9.55  (8.91,10.06) |  | 29414.56 (26980.72,31338.90) | 11.58 (10.59,12.34) |  | 4.32  (4.20,4.43) | 0.66  (0.57,0.75) |
| Western Europe | 136376.65 (127107.86,142174.17) | 22.98 (21.45,23.93) |  | 156637.47 (136856.63,169938.67) | 15.11 (13.53,16.25) |  | 0.45  (0.42,0.49) | -1.40  (-1.45,-1.36) |
| Western Sub-Saharan  Africa | 4161.81 (3556.90,4867.26) | 5.22  (4.48,6.04) |  | 10312.43 (8668.12,12099.87) | 5.98  (5.12,6.88) |  | 3.13  (3.04,3.22) | 0.62  (0.55,0.69) |

Abbreviations: ASIR, age-standardized incidence rate; EAPC, estimated annual percentage change; SDI, Sociodemographic Index; UI, uncertainty interval.

eTable3. DALYs of Stroke between 1990 and 2021 at the Global and Regional Level

| Location | Rate per 100,000 (95% UI) | | | | | | | |
| --- | --- | --- | --- | --- | --- | --- | --- | --- |
|  | 1990 |  |  | 2021 |  |  | 1990-2021 |  |
|  | DALY cases | ASDR |  | DALY cases | ASDR |  | Cases change | EAPC (95% CI) |
| Global | 14396657.72 (13568749.36,15166575.84) | 357.33 (336.62,375.74) |  | 24401100.18 (22689368.55,26161517.73) | 283.24 (263.11,303.33) |  | 1.66  (1.61,1.72) | -0.83  (-0.87,-0.80) |
| Sex | | | | | | | | |
| Male | 7609702.83 (7037903.25,8139914.51) | 405.58 (378.49,431.93) |  | 14167249.34 (12782331.80,15683970.51) | 349.67 (316.68,386.64) |  | 2.01  (1.98,2.05) | -0.52  (-0.55,-0.50) |
| Female | 6786954.89 (6292858.34,7304106.47) | 316.54 (292.93,340.38) |  | 10233850.84 (9257555.91,11064615.26) | 224.30 (203.21,242.65) |  | 1.23  (1.16,1.30) | -1.25  (-1.30,-1.19) |
| Regions | | | | | | | | |
| Andean Latin America | 43988.07 (37837.61,50560.74) | 203.47 (175.15,233.97) |  | 136183.21 (108534.69,167981.61) | 226.76 (180.59,279.80) |  | 3.84  (3.72,3.96) | 0.39  (0.27,0.50) |
| Australasia | 133623.41 (123838.12,143847.28) | 577.93 (535.91,621.91) |  | 166740.10 (147523.69,186890.83) | 327.19 (290.90,364.68) |  | 0.47  (0.36,0.58) | -2.06  (-2.17,-1.96) |
| Caribbean | 83679.93 (78049.17,89688.93) | 317.26 (295.90,339.95) |  | 179751.92 (155874.18,205420.07) | 335.02 (290.41,383.11) |  | 2.67  (2.62,2.72) | 0.33  (0.28,0.37) |
| Central Asia | 140373.97 (132360.71,149146.15) | 280.69 (263.92,298.74) |  | 169952.25 (151537.43,187708.00) | 196.55 (175.59,216.68) |  | 0.81  (0.58,1.04) | -0.88  (-1.00,-0.76) |
| Central Europe | 767091.48 (735174.35,795936.07) | 512.39 (489.92,531.48) |  | 1087562.43 (1003626.42,1168378.10) | 506.48 (467.97,544.57) |  | 1.10  (0.98,1.22) | -0.12  (-0.25,0.00) |
| Central Latin America | 149274.84 (143940.78,154014.73) | 165.76 (159.24,171.33) |  | 594116.79 (529274.15,662367.07) | 231.9 5(206.64,258.57) |  | 4.63  (4.58,4.69) | 1.11  (1.03,1.18) |
| Central Sub-Saharan  Africa | 44003.07 (35102.63,54650.24) | 179.38 (145.64,220.18) |  | 109622.05 (82688.06,146971.08) | 177.79 (135.17,238.59) |  | 3.05  (2.80,3.29) | 0.02  (-0.12,0.17) |
| East Asia | 3691551.51 (3152648.28,4246182.29) | 389.57 (334.25,446.90) |  | 7148995.32 (5822935.26,8561078.94) | 334.50 (274.01,399.93) |  | 2.10  (2.00,2.20) | -0.59  (-0.66,-0.51) |
| Eastern Europe | 1280866.45 (1237775.47,1320907.10) | 455.74 (440.17,469.53) |  | 1465104.15 (1343980.89,1600585.46) | 424.54 (389.69,463.67) |  | 0.10  (-0.04,0.24) | -0.48  (-0.61,-0.35) |
| Eastern Sub-Saharan  Africa | 225496.31 (171119.13,258758.96) | 274.48 (211.05,312.21) |  | 444252.95 (385799.78,525458.32) | 242.13 (211.30,279.08) |  | 2.03  (1.82,2.23) | -0.58  (-0.68,-0.47) |
| High-income  Asia Pacific | 874132.26 (837040.69,905134.70) | 431.84 (411.77,447.55) |  | 1441630.00 (1272495.98,1549758.69) | 331.48 (303.85,352.47) |  | 1.56  (1.48,1.65) | -0.93  (-0.98,-0.88) |
| High-income  North America | 1613201.65 (1529313.04,1679101.05) | 469.38 (446.30,487.53) |  | 1907928.37 (1788110.92,2003823.63) | 316.04 (298.52,330.84) |  | 0.52  (0.46,0.57) | -1.36  (-1.42,-1.31) |
| North Africa and  Middle East | 409788.25 (341302.19,464924.00) | 220.98 (187.75,248.96) |  | 1012651.60 (886198.88,1154502.81) | 209.04 (183.16,237.28) |  | 3.13  (2.96,3.31) | -0.01  (-0.15,0.12) |
| Oceania | 5152.78 (4141.84,6362.22) | 154.39 (127.38,186.63) |  | 11642.97 (9764.00,13750.35) | 136.65 (116.10,159.45) |  | 2.71  (2.56,2.86) | -0.39  (-0.48,-0.29) |
| South Asia | 787250.63 (675897.90,889379.65) | 119.12 (101.91,135.16) |  | 1908668.14 (1711982.01,2155055.24) | 120.37 (108.30,135.30) |  | 2.82  (2.71,2.94) | -0.10  (-0.21,0.01) |
| Southeast Asia | 735821.83 (612421.70,841711.89) | 257.93 (218.13,292.17) |  | 2166650.37 (1868880.14,2456349.67) | 313.37 (270.75,353.54) |  | 3.51  (3.47,3.56) | 0.61  (0.55,0.67) |
| Southern  Latin America | 206237.38 (189818.49,224030.55) | 445.86 (410.18,484.75) |  | 349581.13 (311369.42,391893.39) | 407.99 (363.05,457.56) |  | 1.97  (1.83,2.11) | -0.04  (-0.19,0.11) |
| Southern Sub-Saharan  Africa | 60914.31 (54661.61,72328.10) | 207.84 (185.42,251.15) |  | 166061.78 (149959.40,186753.43) | 270.64 (244.94,301.85) |  | 3.44  (3.14,3.74) | 1.06  (0.78,1.35) |
| Tropical  Latin America | 218356.84 (209208.99,228658.47) | 224.28 (213.27,235.19) |  | 744957.01 (694956.43,786849.09) | 286.22 (266.67,302.45) |  | 4.06  (3.94,4.18) | 0.77  (0.67,0.87) |
| Western Europe | 2815471.87 (2680130.07,2917394.76) | 498.57 (476.35,516.24) |  | 2912355.20 (2641021.36,3112518.77) | 326.82 (301.67,347.23) |  | 0.10  (0.06,0.14) | -1.41  (-1.46,-1.36) |
| Western Sub-Saharan  Africa | 110380.88 (93741.12,130867.07) | 120.11 (102.58,141.26) |  | 276692.44 (224567.81,328426.26) | 132.05 (110.39,155.48) |  | 3.15  (3.06,3.25) | 0.46  (0.40,0.52) |

Abbreviations: ASIR, age-standardized incidence rate; EAPC, estimated annual percentage change; SDI, Sociodemographic Index; UI, uncertainty interval.

**eTable4**. Incidence of CRC Between 1990 and 2021 at the 204 Countries and Territories Level

| Location | Rate per 100,000 (95% UI) | | | | | | | |
| --- | --- | --- | --- | --- | --- | --- | --- | --- |
|  | 1990 |  |  | 2021 |  |  | 1990-2021 |  |
|  | Incidence cases | ASIR |  | Incidence cases | ASIR |  | Cases change | EAPC (95% CI) |
| Countries | | | | | | | | |
| American Samoa | 3.79 (3.21,4.45) | 17.23 (14.76,20.19) |  | 9.05 (7.52,11.18) | 19.36 (16.14,23.75) |  | 2.78 (2.67,2.88) | 0.45 (0.35,0.55) |
| Antigua and Barbuda | 11.16 (10.16,12.22) | 20.46 (18.63,22.43) |  | 34.21 (31.16,37.47) | 32.44 (29.54,35.42) |  | 3.77 (3.60,3.95) | 1.50 (1.32,1.68) |
| Arab Republic of Egypt | 1783.29 (1588.88,1999.47) | 6.32 (5.59,7.20) |  | 8121.03 (6619.62,9961.30) | 12.57 (10.41,15.33) |  | 5.46 (5.27,5.65) | 2.82 (2.56,3.08) |
| Argentine Republic | 8203.66 (7456.52,8941.28) | 25.72 (23.30,28.03) |  | 16145.42 (14155.19,18142.98) | 28.79 (25.21,32.36) |  | 2.44 (2.18,2.69) | 0.61 (0.37,0.85) |
| Australia | 9465.16 (8607.42,10354.72) | 48.52 (44.14,53.17) |  | 18938.72 (16587.70,21680.37) | 42.68 (37.58,48.59) |  | 2.10 (1.95,2.25) | -0.55 (-0.71,-0.40) |
| Barbados | 94.61 (85.53,104.53) | 32.11 (29.06,35.39) |  | 262.56 (206.04,322.71) | 50.65 (39.74,62.59) |  | 3.65 (3.47,3.82) | 1.74 (1.45,2.04) |
| Belize | 8.77 (8.01,9.66) | 9.36 (8.54,10.33) |  | 50.07 (43.49,58.53) | 16.66 (14.53,19.41) |  | 5.97 (5.67,6.27) | 1.94 (1.52,2.36) |
| Bermuda | 35.84 (32.62,39.35) | 57.94 (52.60,63.69) |  | 84.14 (70.18,104.70) | 61.79 (51.46,77.11) |  | 3.02 (2.89,3.16) | 0.33 (0.18,0.47) |
| Bolivarian Republic of Venezuela | 1141.08 (1049.16,1231.60) | 11.77 (10.78,12.73) |  | 5430.61 (3985.76,7073.16) | 18.16 (13.38,23.62) |  | 5.32 (5.09,5.55) | 1.41 (1.21,1.60) |
| Bosnia and Herzegovina | 712.20 (620.59,806.65) | 17.60 (15.35,19.89) |  | 1933.79 (1493.91,2415.04) | 30.79 (23.78,38.32) |  | 3.85 (3.42,4.29) | 2.37 (2.06,2.68) |
| Brunei Darussalam | 36.34 (29.02,43.87) | 35.15 (28.37,42.52) |  | 103.85 (86.89,122.29) | 30.04 (25.37,35.53) |  | 3.90 (3.58,4.22) | -0.01 (-0.26,0.24) |
| Burkina Faso | 206.93 (165.74,258.73) | 5.10 (4.12,6.33) |  | 542.55 (399.28,700.67) | 6.26 (4.71,7.98) |  | 3.42 (3.28,3.57) | 0.88 (0.79,0.96) |
| Canada | 15295.62 (13994.66,16658.79) | 47.02 (43.08,51.17) |  | 30536.91 (26973.45,34052.87) | 43.29 (38.58,47.88) |  | 2.55 (2.33,2.77) | -0.05 (-0.26,0.17) |
| Central African Republic | 106.58 (70.23,144.06) | 9.47 (6.68,12.84) |  | 202.94 (134.67,291.70) | 9.11 (6.39,12.71) |  | 2.15 (2.09,2.21) | -0.11 (-0.17,-0.05) |
| Commonwealth of Dominica | 11.01 (9.82,12.64) | 18.43 (16.49,20.95) |  | 20.33 (17.15,24.64) | 24.62 (20.79,29.66) |  | 2.04 (1.98,2.11) | 0.97 (0.86,1.08) |
| Commonwealth of the Bahamas | 40.44 (36.47,44.76) | 25.60 (23.11,28.31) |  | 146.60 (116.12,182.11) | 36.11 (28.86,44.63) |  | 4.66 (4.44,4.88) | 1.49 (1.28,1.70) |
| Cook Islands | 1.14 (0.94,1.34) | 9.45 (7.85,10.99) |  | 2.42 (1.93,3.04) | 9.43 (7.53,11.93) |  | 2.31 (2.17,2.45) | -0.17 (-0.31,-0.03) |
| Czech Republic | 6585.14 (5882.20,7386.11) | 47.30 (42.43,53.08) |  | 8644.29 (7239.37,10237.41) | 40.24 (33.58,47.83) |  | 0.66 (0.43,0.89) | -0.88 (-1.17,-0.58) |
| Democratic People's Republic of Korea | 2256.58 (1640.34,3096.62) | 13.73 (10.14,18.57) |  | 5148.10 (3462.79,7554.28) | 15.43 (10.42,22.53) |  | 2.87 (2.74,3.00) | 0.58 (0.47,0.68) |
| Democratic Republic of Sao Tome and Principe | 5.15 (4.32,6.08) | 8.31 (6.98,9.79) |  | 12.48 (10.13,15.50) | 12.15 (9.98,14.94) |  | 2.99 (2.83,3.15) | 1.40 (1.34,1.46) |
| Democratic Republic of the Congo | 978.21 (757.64,1259.11) | 6.59 (5.14,8.58) |  | 2447.68 (1696.31,3516.88) | 6.90 (4.78,9.92) |  | 2.99 (2.69,3.29) | 0.20 (-0.02,0.43) |
| Democratic Republic of Timor-Leste | 25.05 (17.69,33.44) | 8.77 (6.39,11.43) |  | 90.87 (68.74,118.62) | 10.66 (8.11,13.78) |  | 4.69 (4.44,4.93) | 0.81 (0.61,1.01) |
| Democratic Socialist Republic of Sri Lanka | 605.39 (530.99,683.75) | 5.68 (4.98,6.44) |  | 2049.56 (1348.19,2868.97) | 7.54 (4.99,10.49) |  | 4.44 (4.27,4.62) | 1.35 (1.17,1.52) |
| Dominican Republic | 355.85 (296.31,417.55) | 9.94 (8.25,11.70) |  | 1547.38 (1214.25,1967.66) | 15.45 (12.13,19.74) |  | 5.10 (4.79,5.42) | 1.79 (1.56,2.01) |
| Eastern Republic of Uruguay | 1294.09 (1182.16,1411.08) | 33.18 (30.31,36.19) |  | 2416.34 (2144.91,2726.76) | 43.22 (38.45,48.54) |  | 1.85 (1.71,1.99) | 0.71 (0.57,0.84) |
| Federal Democratic Republic of Ethiopia | 4001.37 (2232.01,4938.43) | 21.32 (12.46,26.19) |  | 6703.07 (5577.04,8035.38) | 16.41 (13.70,19.54) |  | 1.31 (0.95,1.66) | -1.15 (-1.37,-0.93) |
| Federal Democratic Republic of Nepal | 406.58 (284.87,555.94) | 4.22 (3.06,5.72) |  | 1130.30 (837.74,1498.92) | 4.85 (3.61,6.39) |  | 3.58 (3.19,3.97) | 0.53 (0.17,0.89) |
| Federal Republic of Germany | 60067.58 (54298.50,65471.21) | 46.50 (42.18,50.68) |  | 73768.28 (62963.76,83617.31) | 38.53 (33.72,43.31) |  | 0.44 (0.29,0.60) | -0.91 (-1.08,-0.75) |
| Federal Republic of Nigeria | 2192.28 (1708.59,2782.25) | 5.19 (4.10,6.51) |  | 5216.40 (4014.99,6632.46) | 6.08 (4.88,7.47) |  | 2.94 (2.80,3.08) | 0.67 (0.59,0.76) |
| Federal Republic of Somalia | 236.72 (153.00,342.35) | 9.76 (6.52,14.33) |  | 600.15 (383.01,898.45) | 9.93 (6.34,14.70) |  | 3.22 (3.15,3.29) | 0.19 (0.13,0.24) |
| Federated States of Micronesia | 6.34 (4.96,8.18) | 13.24 (10.48,16.86) |  | 10.23 (7.30,14.05) | 14.14 (10.28,19.12) |  | 1.56 (1.41,1.71) | 0.22 (0.17,0.26) |
| Federative Republic of Brazil | 9684.81 (9201.55,10166.12) | 11.10 (10.42,11.68) |  | 43392.08 (40050.05,46280.20) | 17.23 (15.86,18.39) |  | 4.96 (4.82,5.10) | 1.40 (1.28,1.52) |
| French Republic | 34214.81 (31352.77,36919.20) | 40.83 (37.61,44.07) |  | 58098.94 (49412.92,66071.35) | 41.80 (36.04,47.25) |  | 1.99 (1.87,2.10) | 0.29 (0.16,0.41) |
| Gabonese Republic | 78.66 (47.32,111.68) | 14.03 (8.63,19.94) |  | 158.33 (114.52,209.76) | 15.45 (11.31,20.01) |  | 2.24 (2.10,2.39) | 0.28 (0.22,0.34) |
| Georgia | 875.35 (768.81,989.87) | 13.94 (12.27,15.73) |  | 1093.94 (938.29,1263.39) | 18.73 (16.08,21.69) |  | 1.63 (1.18,2.09) | 1.97 (1.52,2.43) |
| Grand Duchy of Luxembourg | 245.77 (227.69,262.26) | 45.12 (41.89,48.14) |  | 402.88 (355.88,451.70) | 37.51 (33.20,42.15) |  | 1.72 (1.51,1.94) | -0.48 (-0.80,-0.17) |
| Greenland | 17.11 (14.98,19.90) | 52.06 (45.88,60.38) |  | 26.10 (21.97,31.44) | 38.22 (32.36,45.88) |  | 1.53 (1.45,1.60) | -0.95 (-1.04,-0.87) |
| Grenada | 13.84 (11.94,15.94) | 19.19 (16.50,22.10) |  | 35.14 (29.54,41.35) | 31.36 (26.48,36.76) |  | 3.26 (3.12,3.40) | 1.63 (1.37,1.89) |
| Guam | 15.06 (13.01,17.05) | 20.36 (17.35,23.07) |  | 35.30 (30.30,40.89) | 16.97 (14.62,19.62) |  | 3.28 (3.05,3.50) | 0.01 (-0.27,0.29) |
| Hashemite Kingdom of Jordan | 193.65 (157.36,231.96) | 13.77 (11.33,16.43) |  | 1207.27 (926.10,1651.68) | 15.51 (11.84,20.96) |  | 6.31 (6.11,6.52) | 0.64 (0.38,0.90) |
| Hellenic Republic | 4646.26 (4239.16,5054.70) | 30.43 (27.82,33.05) |  | 8051.37 (7051.24,9006.38) | 33.38 (29.96,37.16) |  | 1.52 (1.28,1.75) | -0.04 (-0.25,0.17) |
| Hungary | 6010.31 (5124.56,6886.87) | 40.94 (34.94,46.76) |  | 9406.86 (7835.71,11264.60) | 49.51 (40.97,59.24) |  | 1.37 (1.07,1.67) | 0.44 (0.13,0.75) |
| Independent State of Papua New Guinea | 78.08 (55.50,106.10) | 4.15 (3.05,5.48) |  | 210.89 (160.44,265.76) | 3.94 (3.08,4.91) |  | 3.17 (2.97,3.36) | -0.29 (-0.38,-0.20) |
| Independent State of Samoa | 8.52 (6.90,10.17) | 10.43 (8.48,12.41) |  | 16.48 (13.14,20.44) | 11.59 (9.31,14.21) |  | 2.08 (2.03,2.13) | 0.28 (0.22,0.34) |
| Ireland | 1853.48 (1704.82,2002.33) | 45.39 (41.85,48.86) |  | 3242.63 (2784.78,3698.81) | 40.89 (35.16,46.57) |  | 2.21 (2.08,2.35) | 0.00 (-0.12,0.13) |
| Islamic Republic of Afghanistan | 864.19 (312.28,1353.49) | 12.41 (4.86,19.20) |  | 1550.86 (674.53,2451.88) | 14.16 (6.79,21.48) |  | 1.85 (1.59,2.11) | 0.57 (0.46,0.67) |
| Islamic Republic of Iran | 2318.68 (1931.65,2681.58) | 8.93 (7.51,10.30) |  | 10423.48 (9025.81,11676.71) | 13.19 (11.44,14.76) |  | 5.50 (5.25,5.74) | 1.68 (1.45,1.92) |
| Islamic Republic of Mauritania | 61.84 (48.07,76.09) | 6.47 (5.05,7.96) |  | 161.86 (118.30,217.62) | 7.93 (5.79,10.59) |  | 3.21 (2.94,3.48) | 0.66 (0.47,0.86) |
| Islamic Republic of Pakistan | 2882.62 (2427.17,3308.12) | 5.16 (4.32,5.91) |  | 8449.72 (6886.86,10537.42) | 6.88 (5.67,8.56) |  | 3.23 (3.10,3.36) | 0.66 (0.45,0.86) |
| Jamaica | 327.41 (290.66,363.06) | 18.11 (16.08,20.10) |  | 975.68 (738.37,1303.27) | 31.70 (23.99,42.39) |  | 3.57 (3.18,3.96) | 1.84 (1.44,2.23) |
| Japan | 73089.48 (68829.35,75765.47) | 43.15 (40.48,44.79) |  | 171043.28 (148912.54,184888.97) | 48.70 (44.07,51.75) |  | 2.69 (2.54,2.84) | 0.29 (0.19,0.39) |
| Kingdom of Bahrain | 25.77 (22.57,29.62) | 15.46 (13.59,17.92) |  | 162.42 (128.32,222.71) | 18.79 (15.12,25.02) |  | 6.15 (6.04,6.26) | 0.31 (0.04,0.58) |
| Kingdom of Belgium | 7025.37 (6274.39,7639.30) | 45.18 (40.33,49.22) |  | 8473.09 (7377.90,9570.32) | 36.35 (31.83,40.73) |  | 0.73 (0.59,0.86) | -0.65 (-0.79,-0.50) |
| Kingdom of Bhutan | 11.78 (7.38,16.23) | 4.67 (2.88,6.32) |  | 31.99 (23.90,41.72) | 5.26 (3.97,6.84) |  | 3.28 (3.15,3.41) | 0.36 (0.22,0.50) |
| Kingdom of Cambodia | 609.35 (394.73,809.98) | 13.21 (8.80,17.23) |  | 2113.29 (1567.29,2757.01) | 16.99 (12.67,21.96) |  | 4.21 (4.06,4.37) | 0.83 (0.73,0.92) |
| Kingdom of Denmark | 3061.78 (2767.93,3345.26) | 37.77 (34.22,41.26) |  | 5273.54 (4535.03,6008.41) | 43.45 (37.30,49.33) |  | 1.68 (1.46,1.90) | 0.38 (0.07,0.69) |
| Kingdom of Eswatini | 33.48 (23.14,43.25) | 12.16 (8.40,15.61) |  | 94.15 (60.39,130.19) | 16.74 (10.96,22.46) |  | 3.61 (3.01,4.22) | 1.41 (0.92,1.89) |
| Kingdom of Lesotho | 56.86 (43.25,73.30) | 7.01 (5.36,8.99) |  | 148.07 (103.30,212.08) | 14.06 (10.03,19.74) |  | 3.73 (3.21,4.26) | 3.02 (2.58,3.46) |
| Kingdom of Morocco | 964.93 (749.85,1200.42) | 6.69 (5.21,8.23) |  | 3999.96 (2883.37,5337.59) | 11.56 (8.38,15.19) |  | 4.93 (4.76,5.09) | 1.98 (1.86,2.11) |
| Kingdom of Norway | 2963.60 (2750.92,3107.23) | 43.22 (40.43,45.28) |  | 4803.99 (4273.62,5204.14) | 47.11 (42.42,50.84) |  | 1.58 (1.50,1.66) | 0.34 (0.17,0.52) |
| Kingdom of Saudi Arabia | 435.04 (311.40,573.92) | 7.05 (5.15,9.25) |  | 3459.78 (2651.23,4327.26) | 14.88 (12.12,18.16) |  | 7.15 (6.97,7.32) | 2.79 (2.46,3.11) |
| Kingdom of Spain | 19922.32 (18255.32,21456.65) | 36.67 (33.70,39.62) |  | 44609.17 (38459.01,50136.97) | 46.22 (40.40,51.48) |  | 2.82 (2.53,3.11) | 0.88 (0.65,1.10) |
| Kingdom of Sweden | 5317.60 (4791.21,5840.78) | 35.01 (31.76,38.28) |  | 7578.32 (6357.65,8860.36) | 34.34 (29.04,40.05) |  | 1.25 (1.07,1.44) | 0.10 (-0.15,0.35) |
| Kingdom of Thailand | 5659.34 (4765.51,6607.40) | 15.87 (13.34,18.46) |  | 28040.12 (21228.15,35845.11) | 26.25 (19.95,33.54) |  | 5.11 (4.98,5.23) | 1.42 (1.29,1.55) |
| Kingdom of the Netherlands | 14542.30 (13439.03,15688.23) | 72.21 (66.87,77.94) |  | 24518.83 (21694.49,27070.79) | 69.80 (62.21,76.79) |  | 1.77 (1.57,1.97) | -0.07 (-0.31,0.16) |
| Kingdom of Tonga | 4.19 (3.39,5.04) | 7.81 (6.39,9.38) |  | 7.11 (5.49,9.09) | 8.93 (6.93,11.36) |  | 1.58 (1.43,1.74) | 0.37 (0.26,0.49) |
| Kyrgyz Republic | 396.76 (357.30,436.63) | 13.11 (11.85,14.42) |  | 474.90 (389.65,569.68) | 9.61 (7.84,11.52) |  | 0.65 (0.27,1.02) | -0.77 (-1.02,-0.52) |
| Lao People's Democratic Republic | 286.99 (153.13,397.29) | 13.60 (7.60,18.56) |  | 689.31 (491.35,908.57) | 14.70 (10.62,19.11) |  | 2.75 (2.65,2.85) | 0.18 (0.15,0.22) |
| Lebanese Republic | 355.25 (238.49,449.76) | 16.92 (11.68,21.13) |  | 1239.64 (1002.83,1584.24) | 20.30 (16.48,25.89) |  | 4.84 (4.54,5.15) | 1.13 (0.88,1.39) |
| Malaysia | 1754.58 (1545.77,1995.00) | 18.96 (16.64,21.49) |  | 7360.29 (6412.69,8288.02) | 26.12 (22.65,29.53) |  | 4.69 (4.53,4.85) | 0.89 (0.76,1.02) |
| Mongolia | 89.55 (69.77,114.23) | 8.26 (6.42,10.49) |  | 238.24 (185.41,295.47) | 10.01 (7.82,12.45) |  | 3.26 (2.92,3.60) | 0.55 (0.40,0.71) |
| Montenegro | 144.49 (124.00,169.42) | 23.09 (19.82,27.02) |  | 307.30 (255.27,374.65) | 31.46 (26.15,38.30) |  | 2.83 (2.73,2.94) | 1.32 (1.19,1.46) |
| New Zealand | 2378.09 (2138.90,2617.66) | 60.89 (54.87,66.94) |  | 4342.02 (3762.51,4951.74) | 51.08 (44.46,58.12) |  | 1.89 (1.82,1.96) | -0.64 (-0.71,-0.57) |
| North Macedonia | 368.76 (318.42,424.03) | 20.06 (17.33,23.15) |  | 949.89 (743.10,1164.13) | 29.07 (23.07,35.39) |  | 3.39 (3.02,3.76) | 1.37 (0.99,1.74) |
| Northern Mariana Islands | 3.65 (2.80,4.80) | 19.92 (16.02,24.84) |  | 11.39 (9.69,12.73) | 22.37 (19.09,25.01) |  | 3.86 (3.72,4.00) | 0.25 (0.07,0.43) |
| Palestine | 185.79 (139.03,240.22) | 21.71 (16.41,27.75) |  | 591.32 (485.20,707.46) | 23.13 (19.17,27.26) |  | 3.88 (3.80,3.97) | 0.24 (0.13,0.35) |
| People's Democratic Republic of Algeria | 631.83 (525.37,750.96) | 5.84 (4.87,6.86) |  | 2355.07 (1823.70,3031.79) | 6.95 (5.39,8.79) |  | 4.44 (4.29,4.59) | 0.83 (0.67,0.99) |
| People's Republic of Bangladesh | 2298.07 (1767.95,3004.26) | 4.75 (3.67,6.17) |  | 6093.88 (4492.00,8429.83) | 4.41 (3.29,6.03) |  | 3.42 (3.18,3.67) | -0.28 (-0.40,-0.16) |
| People's Republic of China | 158389.30 (135418.51,182577.30) | 19.04 (16.46,21.81) |  | 658321.36 (531995.02,798063.00) | 31.44 (25.53,37.97) |  | 4.87 (4.70,5.05) | 1.75 (1.64,1.87) |
| Plurinational State of Bolivia | 419.82 (265.70,567.75) | 13.62 (8.73,18.37) |  | 1434.38 (978.35,2003.28) | 16.31 (11.17,22.63) |  | 4.25 (4.18,4.32) | 0.62 (0.59,0.65) |
| Portuguese Republic | 4578.01 (4058.88,5138.59) | 33.56 (29.66,37.72) |  | 9840.59 (8400.70,11273.58) | 42.44 (36.62,48.89) |  | 2.72 (2.35,3.09) | 0.94 (0.60,1.27) |
| Principality of Andorra | 28.73 (20.67,40.70) | 50.02 (35.95,70.69) |  | 68.80 (46.72,93.00) | 44.68 (30.29,60.44) |  | 2.74 (2.40,3.08) | -0.07 (-0.34,0.20) |
| Principality of Monaco | 35.49 (27.08,42.82) | 51.04 (39.38,61.91) |  | 65.10 (51.42,78.45) | 68.33 (54.05,83.19) |  | 2.06 (1.91,2.22) | 1.10 (0.93,1.26) |
| Puerto Rico | 1052.95 (961.51,1152.41) | 29.15 (26.59,32.00) |  | 3078.82 (2548.00,3713.46) | 46.52 (38.42,56.28) |  | 3.63 (3.22,4.04) | 1.61 (1.30,1.91) |
| Republic of Albania | 174.84 (146.88,209.07) | 8.89 (7.50,10.57) |  | 500.52 (386.24,670.21) | 11.48 (8.85,15.39) |  | 4.01 (3.81,4.22) | 1.31 (1.11,1.51) |
| Republic of Angola | 298.79 (216.23,386.81) | 7.86 (5.79,9.96) |  | 1041.34 (760.48,1381.94) | 9.04 (6.63,11.83) |  | 4.24 (3.98,4.50) | 0.48 (0.34,0.62) |
| Republic of Armenia | 493.65 (455.02,531.53) | 17.77 (16.29,19.16) |  | 847.11 (740.41,969.38) | 19.55 (17.07,22.33) |  | 2.09 (1.91,2.28) | 0.71 (0.47,0.94) |
| Republic of Austria | 5056.09 (4622.71,5449.17) | 42.61 (38.96,45.94) |  | 5239.96 (4580.34,6008.72) | 29.27 (25.66,33.38) |  | 0.18 (0.07,0.29) | -1.25 (-1.40,-1.10) |
| Republic of Azerbaijan | 551.29 (463.22,634.90) | 10.62 (8.95,12.19) |  | 1033.00 (781.44,1311.21) | 9.79 (7.42,12.32) |  | 2.32 (1.90,2.75) | 0.13 (-0.21,0.47) |
| Republic of Belarus | 3156.07 (2730.43,3609.06) | 24.31 (21.10,27.68) |  | 5805.74 (4402.20,7410.17) | 36.09 (27.38,45.99) |  | 1.30 (0.92,1.69) | 0.68 (0.32,1.04) |
| Republic of Benin | 95.58 (80.56,113.06) | 4.95 (4.18,5.83) |  | 283.88 (213.26,375.44) | 5.85 (4.47,7.48) |  | 3.77 (3.61,3.93) | 0.71 (0.61,0.81) |
| Republic of Botswana | 53.68 (38.84,69.45) | 10.20 (7.46,12.97) |  | 153.68 (113.95,213.28) | 11.05 (8.43,14.95) |  | 3.39 (3.21,3.57) | 0.34 (0.14,0.53) |
| Republic of Bulgaria | 3618.28 (3193.22,4119.87) | 29.51 (26.23,33.49) |  | 6487.63 (5329.49,7840.90) | 46.04 (37.65,55.79) |  | 2.36 (2.19,2.53) | 1.88 (1.72,2.05) |
| Republic of Burundi | 219.29 (166.05,278.52) | 9.52 (7.35,11.98) |  | 395.42 (280.69,557.17) | 8.43 (6.15,11.61) |  | 1.70 (1.30,2.10) | -0.68 (-0.84,-0.53) |
| Republic of Cabo Verde | 8.87 (7.45,10.29) | 3.79 (3.18,4.39) |  | 45.95 (36.03,55.31) | 10.83 (8.47,13.09) |  | 5.06 (4.69,5.43) | 2.93 (2.54,3.32) |
| Republic of Cameroon | 275.23 (232.11,327.50) | 6.58 (5.58,7.77) |  | 938.85 (647.22,1275.64) | 7.91 (5.68,10.58) |  | 4.19 (4.11,4.27) | 0.72 (0.64,0.79) |
| Republic of Chad | 114.99 (93.49,143.92) | 4.21 (3.43,5.30) |  | 346.30 (255.13,452.17) | 6.32 (4.73,8.20) |  | 3.87 (3.79,3.94) | 1.55 (1.44,1.66) |
| Republic of Chile | 1484.22 (1353.24,1616.47) | 15.16 (13.85,16.54) |  | 6183.70 (5401.12,6996.06) | 24.09 (21.07,27.25) |  | 5.13 (4.99,5.28) | 1.87 (1.75,2.00) |
| Republic of Colombia | 2135.65 (1965.45,2310.88) | 12.34 (11.27,13.43) |  | 12242.68 (10081.47,14974.08) | 22.28 (18.37,27.26) |  | 5.61 (5.50,5.71) | 1.71 (1.57,1.84) |
| Republic of Costa Rica | 272.16 (245.04,297.77) | 15.64 (14.09,17.12) |  | 1973.89 (1705.93,2321.01) | 35.99 (31.18,42.31) |  | 6.94 (6.71,7.18) | 3.05 (2.82,3.28) |
| Republic of Croatia | 154.15 (123.33,186.00) | 4.10 (3.35,4.88) |  | 465.01 (339.97,658.66) | 4.31 (3.27,5.89) |  | 3.44 (3.30,3.57) | 0.12 (0.04,0.19) |
| Republic of Cuba | 2097.17 (1841.44,2365.47) | 35.68 (31.33,40.18) |  | 4478.08 (3818.70,5156.31) | 49.75 (42.40,57.25) |  | 2.67 (2.42,2.92) | 1.26 (1.07,1.46) |
| Republic of Cyprus | 3192.56 (2921.30,3449.60) | 31.05 (28.42,33.55) |  | 9455.85 (7941.78,11026.68) | 48.47 (40.77,56.59) |  | 3.68 (3.60,3.76) | 1.50 (1.41,1.59) |
| Republic of C么te d'Ivoire | 214.37 (178.32,254.94) | 29.72 (24.72,35.29) |  | 710.34 (587.77,848.11) | 34.23 (28.58,40.59) |  | 4.63 (4.30,4.95) | 1.10 (0.78,1.42) |
| Republic of Djibouti | 13.01 (8.88,18.33) | 9.75 (6.91,13.75) |  | 82.08 (57.48,117.97) | 13.50 (9.66,18.59) |  | 6.16 (6.08,6.24) | 1.09 (1.03,1.15) |
| Republic of Ecuador | 397.60 (360.56,431.09) | 7.77 (7.03,8.42) |  | 2122.35 (1647.99,2690.93) | 13.04 (10.17,16.50) |  | 5.90 (5.53,6.28) | 1.98 (1.66,2.30) |
| Republic of El Salvador | 189.68 (172.93,208.04) | 6.28 (5.71,6.90) |  | 914.11 (742.08,1126.17) | 14.81 (12.01,18.31) |  | 5.21 (4.88,5.54) | 2.79 (2.50,3.09) |
| Republic of Equatorial Guinea | 16.15 (11.36,21.11) | 8.50 (6.20,11.00) |  | 62.40 (41.30,88.48) | 12.25 (8.33,17.07) |  | 4.90 (4.55,5.25) | 1.47 (1.29,1.64) |
| Republic of Estonia | 607.13 (552.29,666.26) | 29.45 (26.73,32.38) |  | 1121.89 (912.60,1331.81) | 40.93 (33.39,48.65) |  | 2.01 (1.79,2.24) | 0.98 (0.80,1.16) |
| Republic of Fiji | 35.12 (28.25,42.37) | 9.77 (7.85,11.67) |  | 82.76 (60.55,108.06) | 11.22 (8.35,14.50) |  | 3.01 (2.77,3.25) | 0.43 (0.22,0.63) |
| Republic of Finland | 1913.41 (1761.89,2065.12) | 26.64 (24.51,28.73) |  | 3888.78 (3382.90,4367.90) | 30.63 (26.84,34.28) |  | 2.46 (2.37,2.55) | 0.54 (0.47,0.61) |
| Republic of Ghana | 310.73 (243.72,392.16) | 5.30 (4.19,6.63) |  | 1153.11 (896.82,1445.97) | 7.41 (5.81,9.21) |  | 4.49 (4.44,4.55) | 1.27 (1.21,1.33) |
| Republic of Guatemala | 171.20 (157.48,186.47) | 5.20 (4.78,5.66) |  | 1085.76 (931.21,1275.93) | 9.79 (8.42,11.48) |  | 6.18 (5.79,6.57) | 1.87 (1.49,2.24) |
| Republic of Guinea | 131.37 (105.91,160.68) | 4.07 (3.30,4.97) |  | 267.73 (197.75,364.09) | 4.85 (3.63,6.48) |  | 2.21 (2.02,2.41) | 0.68 (0.59,0.76) |
| Republic of Guinea-Bissau | 28.10 (17.81,36.11) | 7.29 (4.73,9.20) |  | 58.65 (43.32,74.58) | 8.48 (6.36,10.64) |  | 2.63 (2.47,2.78) | 0.70 (0.62,0.78) |
| Republic of Guyana | 52.94 (46.77,60.17) | 14.12 (12.48,15.94) |  | 125.24 (94.36,162.54) | 19.48 (14.82,25.11) |  | 3.14 (2.88,3.41) | 1.21 (0.92,1.50) |
| Republic of Haiti | 503.02 (321.98,665.65) | 16.22 (10.85,21.16) |  | 1158.72 (780.42,1661.76) | 16.53 (11.29,23.36) |  | 2.90 (2.82,2.98) | 0.22 (0.15,0.29) |
| Republic of Honduras | 101.10 (85.90,119.28) | 4.80 (4.08,5.67) |  | 522.46 (410.29,681.85) | 8.32 (6.62,10.76) |  | 5.67 (5.51,5.82) | 1.93 (1.76,2.10) |
| Republic of Iceland | 96.42 (87.95,105.65) | 33.70 (30.74,36.90) |  | 189.24 (161.88,221.16) | 32.67 (28.14,38.09) |  | 2.26 (2.06,2.46) | -0.06 (-0.29,0.17) |
| Republic of India | 22539.15 (19170.05,25499.04) | 4.61 (3.89,5.25) |  | 69409.24 (61759.49,78517.20) | 5.69 (5.05,6.45) |  | 3.61 (3.44,3.79) | 0.56 (0.39,0.73) |
| Republic of Indonesia | 10053.67 (7779.61,12204.89) | 9.94 (7.74,11.97) |  | 35784.91 (26676.54,45003.32) | 15.03 (11.40,18.74) |  | 4.06 (4.02,4.10) | 1.33 (1.26,1.40) |
| Republic of Iraq | 537.30 (428.28,656.77) | 6.45 (5.16,7.92) |  | 2511.86 (1889.90,3240.54) | 10.04 (7.60,12.68) |  | 5.43 (5.11,5.74) | 1.60 (1.32,1.87) |
| Republic of Italy | 33896.46 (31765.90,35704.27) | 38.15 (35.83,40.12) |  | 56714.21 (49886.76,61489.05) | 39.65 (35.64,42.66) |  | 1.65 (1.34,1.97) | 0.05 (-0.26,0.36) |
| Republic of Kazakhstan | 2468.68 (2177.20,2774.25) | 19.18 (16.84,21.61) |  | 2804.57 (2375.97,3267.97) | 15.43 (13.06,17.98) |  | 0.78 (0.54,1.02) | -0.28 (-0.47,-0.08) |
| Republic of Kenya | 419.96 (337.60,507.75) | 5.01 (4.05,6.05) |  | 1800.68 (1480.40,2267.47) | 7.89 (6.48,9.79) |  | 5.18 (5.01,5.36) | 1.88 (1.68,2.09) |
| Republic of Kiribati | 3.29 (2.67,4.09) | 8.95 (7.28,11.28) |  | 6.71 (5.06,8.98) | 9.43 (7.43,12.48) |  | 2.27 (2.25,2.30) | 0.03 (-0.06,0.11) |
| Republic of Korea | 5634.02 (4861.18,6402.92) | 19.25 (16.59,21.91) |  | 33144.71 (27171.49,39015.40) | 35.48 (29.08,41.81) |  | 6.11 (5.71,6.51) | 2.10 (1.71,2.50) |
| Republic of Latvia | 922.13 (815.96,1041.06) | 25.70 (22.67,29.05) |  | 1183.00 (968.38,1410.39) | 30.02 (24.50,35.79) |  | 0.93 (0.77,1.09) | 0.53 (0.39,0.68) |
| Republic of Liberia | 54.99 (44.08,68.48) | 5.02 (4.02,6.20) |  | 119.77 (73.91,181.42) | 5.94 (3.78,8.87) |  | 2.88 (2.54,3.21) | 0.89 (0.67,1.12) |
| Republic of Lithuania | 1129.36 (1032.96,1225.53) | 25.05 (22.93,27.21) |  | 1775.38 (1472.40,2094.02) | 30.85 (25.55,36.25) |  | 1.39 (1.22,1.56) | 0.56 (0.42,0.70) |
| Republic of Madagascar | 416.60 (335.27,504.40) | 8.26 (6.72,9.96) |  | 935.85 (672.55,1228.03) | 8.48 (6.16,10.94) |  | 2.80 (2.49,3.10) | 0.18 (0.06,0.30) |
| Republic of Malawi | 152.75 (126.09,188.64) | 4.07 (3.38,4.94) |  | 355.50 (274.54,490.05) | 4.84 (3.81,6.50) |  | 2.53 (2.40,2.66) | 0.40 (0.24,0.57) |
| Republic of Maldives | 7.12 (4.02,9.05) | 7.97 (5.06,9.97) |  | 22.58 (18.31,28.14) | 6.59 (5.32,8.14) |  | 3.53 (3.41,3.65) | -0.86 (-0.95,-0.77) |
| Republic of Mali | 265.33 (225.68,312.56) | 6.98 (6.01,8.12) |  | 629.76 (481.78,800.46) | 7.43 (5.79,9.30) |  | 3.25 (3.00,3.49) | 0.41 (0.30,0.52) |
| Republic of Malta | 135.25 (119.65,152.65) | 31.83 (28.19,35.88) |  | 332.57 (280.61,379.82) | 34.01 (28.75,38.87) |  | 2.91 (2.78,3.04) | 0.15 (0.02,0.28) |
| Republic of Mauritius | 74.04 (69.05,79.49) | 10.20 (9.51,10.96) |  | 394.95 (358.26,426.34) | 21.62 (19.58,23.31) |  | 4.88 (4.67,5.10) | 1.77 (1.55,1.99) |
| Republic of Moldova | 1069.83 (958.02,1175.40) | 24.14 (21.66,26.41) |  | 1900.45 (1606.25,2248.52) | 31.64 (26.71,37.44) |  | 2.25 (1.76,2.74) | 1.30 (0.81,1.78) |
| Republic of Mozambique | 157.73 (130.93,186.35) | 3.09 (2.58,3.61) |  | 388.93 (293.03,495.29) | 4.08 (3.11,5.07) |  | 3.41 (3.25,3.56) | 1.35 (1.20,1.50) |
| Republic of Namibia | 37.22 (30.80,43.56) | 5.84 (4.87,6.90) |  | 102.85 (73.60,134.78) | 7.40 (5.50,9.48) |  | 3.14 (2.90,3.38) | 0.70 (0.49,0.91) |
| Republic of Nauru | 0.91 (0.51,1.27) | 19.75 (12.04,27.30) |  | 1.16 (0.69,1.57) | 19.80 (12.26,26.38) |  | 0.51 (0.37,0.65) | -0.09 (-0.19,0.01) |
| Republic of Nicaragua | 90.19 (79.49,102.90) | 5.78 (5.08,6.60) |  | 498.04 (402.61,610.74) | 10.08 (8.17,12.28) |  | 6.07 (5.77,6.38) | 2.27 (1.98,2.57) |
| Republic of Niue | 0.29 (0.24,0.35) | 12.86 (10.50,15.63) |  | 0.33 (0.27,0.41) | 15.64 (12.56,18.92) |  | 0.28 (0.20,0.37) | 0.52 (0.49,0.56) |
| Republic of Palau | 1.59 (1.22,2.09) | 17.18 (13.16,22.26) |  | 3.43 (2.62,4.30) | 16.94 (13.16,21.11) |  | 2.22 (2.10,2.34) | 0.05 (-0.02,0.12) |
| Republic of Panama | 213.86 (194.82,233.33) | 14.27 (12.95,15.57) |  | 1226.65 (939.47,1509.01) | 27.81 (21.29,34.22) |  | 5.98 (5.90,6.05) | 2.33 (2.25,2.41) |
| Republic of Paraguay | 158.39 (135.82,186.88) | 7.19 (6.15,8.49) |  | 852.54 (656.79,1095.31) | 14.76 (11.38,18.97) |  | 5.79 (5.59,5.99) | 2.59 (2.41,2.76) |
| Republic of Peru | 1084.95 (904.84,1269.26) | 9.18 (7.67,10.74) |  | 4895.33 (3651.52,6467.51) | 14.65 (10.91,19.31) |  | 5.12 (4.93,5.31) | 1.50 (1.31,1.69) |
| Republic of Poland | 10699.56 (10229.21,11132.77) | 24.53 (23.44,25.53) |  | 25418.09 (22826.50,27672.24) | 35.32 (31.88,38.43) |  | 2.87 (2.70,3.04) | 1.11 (0.90,1.32) |
| Republic of Rwanda | 309.55 (240.89,384.88) | 10.97 (8.61,13.55) |  | 603.13 (420.90,810.15) | 10.01 (7.04,13.31) |  | 1.83 (1.26,2.41) | -0.97 (-1.28,-0.66) |
| Republic of San Marino | 17.99 (14.84,21.55) | 50.28 (41.55,60.25) |  | 22.62 (15.05,31.53) | 30.45 (20.08,43.72) |  | 1.61 (1.15,2.07) | -0.75 (-1.14,-0.36) |
| Republic of Senegal | 172.10 (143.90,205.78) | 5.57 (4.67,6.70) |  | 530.88 (402.88,699.96) | 7.26 (5.59,9.41) |  | 3.92 (3.77,4.06) | 0.99 (0.84,1.14) |
| Republic of Serbia | 3166.54 (2661.61,3820.75) | 29.90 (25.14,36.39) |  | 5785.19 (4699.05,7054.47) | 35.31 (28.47,43.10) |  | 1.94 (1.74,2.14) | 0.59 (0.46,0.72) |
| Republic of Seychelles | 10.87 (9.71,12.36) | 19.21 (17.18,21.81) |  | 32.16 (27.47,36.83) | 27.82 (23.85,31.88) |  | 3.71 (3.51,3.91) | 1.31 (1.04,1.59) |
| Republic of Sierra Leone | 88.01 (67.82,113.42) | 4.46 (3.47,5.68) |  | 200.60 (146.94,268.10) | 5.56 (4.16,7.37) |  | 2.98 (2.71,3.24) | 1.04 (0.90,1.17) |
| Republic of Singapore | 783.44 (716.23,865.50) | 35.78 (32.53,39.60) |  | 2985.14 (2584.51,3414.72) | 34.92 (30.08,40.08) |  | 4.48 (4.29,4.66) | -0.10 (-0.26,0.07) |
| Republic of Slovenia | 785.14 (695.45,880.41) | 31.70 (28.02,35.65) |  | 1504.71 (1226.00,1851.10) | 34.16 (27.82,42.22) |  | 2.22 (1.88,2.55) | 0.23 (-0.11,0.58) |
| Republic of South Africa | 1917.70 (1675.99,2412.92) | 9.29 (8.04,11.89) |  | 6133.96 (5488.26,6892.11) | 13.46 (12.07,14.96) |  | 3.94 (3.74,4.14) | 1.33 (1.12,1.54) |
| Republic of South Sudan | 246.23 (167.24,350.26) | 9.79 (6.68,13.78) |  | 455.39 (313.94,606.13) | 12.06 (8.35,15.90) |  | 2.10 (1.82,2.38) | 0.76 (0.63,0.88) |
| Republic of Sudan | 657.76 (443.08,938.20) | 6.91 (4.88,9.77) |  | 1901.14 (1246.60,2729.11) | 9.04 (6.06,12.80) |  | 3.43 (3.27,3.58) | 0.94 (0.85,1.03) |
| Republic of Suriname | 42.82 (38.39,47.64) | 17.06 (15.29,18.95) |  | 140.29 (107.27,176.78) | 22.20 (16.99,28.06) |  | 4.40 (4.15,4.65) | 1.19 (0.96,1.42) |
| Republic of Tajikistan | 252.52 (210.61,297.78) | 8.91 (7.46,10.48) |  | 340.26 (256.65,450.62) | 5.56 (4.29,6.93) |  | 0.86 (0.43,1.29) | -1.52 (-1.75,-1.30) |
| Republic of the Congo | 112.52 (80.07,144.35) | 10.86 (8.09,13.75) |  | 293.77 (225.50,379.45) | 10.97 (8.68,13.79) |  | 3.05 (2.80,3.30) | -0.06 (-0.19,0.08) |
| Republic of the Gambia | 9.26 (7.17,11.47) | 2.75 (2.20,3.37) |  | 31.72 (23.86,41.36) | 3.31 (2.54,4.27) |  | 3.97 (3.80,4.15) | 0.51 (0.37,0.64) |
| Republic of the Marshall Islands | 1.96 (1.51,2.37) | 12.16 (9.53,14.61) |  | 4.64 (3.35,6.20) | 13.31 (9.85,17.40) |  | 2.92 (2.83,3.01) | 0.30 (0.24,0.36) |
| Republic of the Niger | 115.43 (87.72,148.68) | 4.46 (3.42,5.73) |  | 379.81 (267.26,517.05) | 5.06 (3.63,6.78) |  | 4.20 (4.04,4.36) | 0.65 (0.54,0.76) |
| Republic of the Philippines | 3568.21 (3203.12,4119.34) | 11.51 (10.27,13.65) |  | 14071.55 (11842.12,16616.10) | 16.55 (14.01,19.38) |  | 4.63 (4.58,4.68) | 1.32 (1.27,1.37) |
| Republic of the Union of Myanmar | 2822.73 (1764.47,4076.18) | 11.91 (7.64,16.80) |  | 6684.73 (4898.89,8657.47) | 13.71 (10.09,17.58) |  | 2.67 (2.58,2.75) | 0.35 (0.30,0.41) |
| Republic of Trinidad and Tobago | 189.32 (175.25,204.65) | 23.11 (21.36,25.06) |  | 586.18 (443.13,748.98) | 30.36 (23.02,38.79) |  | 3.85 (3.72,3.99) | 1.01 (0.88,1.14) |
| Republic of Tunisia | 474.87 (395.71,573.33) | 9.79 (8.11,11.78) |  | 1745.38 (1253.01,2397.03) | 13.13 (9.49,17.88) |  | 4.09 (4.00,4.17) | 0.80 (0.74,0.85) |
| Republic of Turkey | 6798.50 (5750.30,7986.07) | 19.44 (16.53,22.91) |  | 21368.79 (17106.99,26283.82) | 22.86 (18.36,28.06) |  | 3.88 (3.55,4.21) | 0.63 (0.29,0.97) |
| Republic of Uganda | 635.91 (512.72,788.56) | 10.23 (8.34,12.54) |  | 1677.07 (1273.00,2243.68) | 11.44 (8.87,14.84) |  | 2.62 (2.41,2.83) | -0.15 (-0.37,0.08) |
| Republic of Uzbekistan | 901.49 (794.36,1019.20) | 7.60 (6.61,8.61) |  | 1776.34 (1425.78,2187.42) | 6.34 (5.11,7.83) |  | 2.22 (1.62,2.83) | -0.48 (-0.97,0.00) |
| Republic of Vanuatu | 6.59 (4.73,8.68) | 10.77 (7.95,13.95) |  | 19.31 (14.53,25.05) | 11.18 (8.62,14.41) |  | 3.39 (3.27,3.51) | 0.01 (-0.07,0.09) |
| Republic of Yemen | 358.20 (240.72,507.75) | 7.23 (5.15,9.90) |  | 1218.84 (790.59,1773.85) | 8.33 (5.43,11.95) |  | 4.21 (4.11,4.32) | 0.58 (0.49,0.67) |
| Republic of Zambia | 282.63 (231.95,344.40) | 10.01 (8.30,12.12) |  | 1026.03 (633.76,1982.45) | 14.58 (9.44,26.19) |  | 4.23 (3.93,4.52) | 1.18 (1.08,1.28) |
| Republic of Zimbabwe | 417.37 (345.07,501.25) | 10.97 (9.12,13.05) |  | 990.70 (750.99,1274.53) | 14.51 (11.28,18.18) |  | 2.78 (2.30,3.26) | 1.17 (0.70,1.64) |
| Romania | 4574.83 (4023.97,5165.70) | 16.35 (14.36,18.47) |  | 14073.76 (11803.56,16881.29) | 38.46 (32.20,45.92) |  | 3.55 (3.30,3.82) | 2.63 (2.39,2.88) |
| Russian Federation | 43502.93 (42101.20,44670.08) | 23.98 (23.17,24.60) |  | 82019.00 (75112.53,88815.26) | 34.18 (31.30,37.02) |  | 1.90 (1.75,2.06) | 1.07 (0.93,1.22) |
| Saint Kitts and Nevis | 8.36 (7.49,9.19) | 22.19 (19.99,24.36) |  | 21.07 (17.42,25.25) | 31.64 (26.54,37.16) |  | 3.49 (3.26,3.73) | 1.66 (1.46,1.86) |
| Saint Lucia | 14.55 (13.32,15.79) | 17.19 (15.79,18.67) |  | 49.10 (40.03,59.61) | 20.58 (16.82,24.97) |  | 3.87 (3.73,4.01) | 0.25 (0.12,0.39) |
| Saint Vincent and the Grenadines | 11.74 (10.71,12.83) | 16.55 (15.09,18.08) |  | 31.88 (28.01,36.66) | 22.65 (19.93,26.01) |  | 3.36 (3.17,3.55) | 1.00 (0.86,1.13) |
| Slovak Republic | 2570.12 (2241.54,2955.74) | 42.68 (37.23,48.97) |  | 5126.64 (4192.63,6105.90) | 53.35 (43.82,63.47) |  | 2.38 (2.32,2.43) | 0.81 (0.72,0.91) |
| Socialist Republic of Viet Nam | 3801.15 (3025.44,4739.14) | 9.38 (7.52,11.63) |  | 19444.23 (14778.41,23609.69) | 19.08 (14.66,23.00) |  | 5.62 (5.56,5.68) | 2.51 (2.40,2.62) |
| Solomon Islands | 12.96 (7.32,18.84) | 9.72 (6.11,13.61) |  | 37.28 (26.34,50.07) | 10.63 (7.70,14.12) |  | 3.36 (3.22,3.50) | 0.26 (0.13,0.40) |
| State of Eritrea | 116.35 (89.56,143.14) | 10.07 (7.94,12.24) |  | 327.29 (239.29,440.99) | 12.11 (9.05,15.78) |  | 3.74 (3.60,3.88) | 0.62 (0.54,0.69) |
| State of Israel | 1628.25 (1452.50,1819.23) | 33.65 (30.02,37.45) |  | 3710.70 (3080.32,4292.56) | 29.81 (25.05,34.37) |  | 2.19 (1.80,2.58) | -0.83 (-1.20,-0.45) |
| State of Kuwait | 59.77 (53.64,66.83) | 9.44 (8.47,10.48) |  | 612.49 (491.04,757.52) | 19.05 (15.40,23.23) |  | 8.09 (7.68,8.51) | 2.79 (2.27,3.30) |
| State of Libya | 316.95 (248.28,404.12) | 16.75 (13.02,21.10) |  | 1172.32 (848.97,1585.53) | 21.49 (15.65,28.68) |  | 4.84 (4.64,5.05) | 1.32 (1.06,1.57) |
| State of Qatar | 20.15 (16.48,24.58) | 18.50 (15.24,22.47) |  | 228.72 (168.22,312.76) | 23.29 (17.72,31.38) |  | 9.37 (8.93,9.80) | 1.14 (0.55,1.72) |
| Sultanate of Oman | 35.69 (25.92,47.91) | 5.20 (3.84,6.87) |  | 130.13 (97.40,176.39) | 6.43 (4.96,8.11) |  | 4.34 (4.20,4.47) | 1.02 (0.80,1.25) |
| Swiss Confederation | 3181.23 (2862.36,3485.70) | 30.47 (27.64,33.26) |  | 5115.92 (4283.09,5905.75) | 28.11 (24.03,32.46) |  | 1.44 (1.19,1.69) | -0.39 (-0.69,-0.09) |
| Syrian Arab Republic | 436.72 (341.33,549.27) | 7.96 (6.28,9.91) |  | 1388.62 (995.80,1898.32) | 10.56 (7.75,14.15) |  | 3.88 (3.67,4.09) | 0.82 (0.67,0.97) |
| Taiwan (Province of China) | 4437.54 (4058.21,4812.92) | 27.15 (24.84,29.44) |  | 21457.69 (18753.14,24088.02) | 51.62 (45.24,57.72) |  | 4.90 (4.47,5.33) | 1.74 (1.31,2.16) |
| Togolese Republic | 56.18 (44.71,71.17) | 4.79 (3.84,6.05) |  | 234.95 (155.73,328.37) | 6.61 (4.53,9.15) |  | 4.87 (4.78,4.97) | 1.17 (1.10,1.25) |
| Tokelau | 0.16 (0.12,0.19) | 11.83 (9.32,14.57) |  | 0.19 (0.15,0.24) | 12.79 (9.97,16.58) |  | 0.50 (0.43,0.58) | 0.21 (0.18,0.25) |
| Turkmenistan | 150.87 (136.82,165.82) | 7.53 (6.81,8.34) |  | 284.62 (212.68,375.14) | 6.79 (5.11,8.87) |  | 2.11 (1.49,2.73) | -0.30 (-0.84,0.24) |
| Tuvalu | 0.77 (0.60,0.98) | 11.90 (9.63,14.95) |  | 1.29 (1.04,1.63) | 12.64 (10.21,15.89) |  | 1.56 (1.51,1.62) | 0.14 (0.11,0.17) |
| Ukraine | 21363.08 (19510.95,23188.73) | 29.65 (27.11,32.18) |  | 19446.72 (14564.24,25204.31) | 25.12 (18.71,32.57) |  | -0.54 (-0.67,-0.41) | -0.72 (-0.84,-0.60) |
| Union of the Comoros | 18.10 (12.25,23.96) | 9.34 (6.63,12.25) |  | 49.76  (35.55,66.89) | 10.56 (7.55,13.96) |  | 3.24 (3.07,3.41) | 0.34 (0.22,0.45) |
| United Arab Emirates | 104.16 (68.64,143.23) | 21.16 (14.40,29.03) |  | 635.77 (456.80,941.06) | 18.79 (14.27,26.88) |  | 6.66 (6.39,6.94) | 1.20 (0.69,1.72) |
| United Kingdom of Great Britain and Northern Ireland | 39088.35 (37237.45,40105.05) | 42.86 (41.02,43.92) |  | 50411.39 (46085.05,52744.07) | 38.75 (35.98,40.37) |  | 0.95 (0.86,1.04) | -0.24 (-0.31,-0.17) |
| United Mexican States | 3317.41 (3196.44,3438.07) | 8.03 (7.71,8.32) |  | 20657.33 (18125.28,23264.84) | 16.16 (14.20,18.20) |  | 6.12 (6.02,6.22) | 2.28 (2.18,2.38) |
| United Republic of Tanzania | 947.32 (763.40,1167.30) | 8.94 (7.31,10.80) |  | 2535.91 (1878.62,3278.41) | 10.15 (7.58,12.91) |  | 3.17 (3.07,3.27) | 0.39 (0.33,0.45) |
| United States of America | 152328.60 (141711.74,158999.16) | 47.35 (44.26,49.35) |  | 214114.60 (197870.73,225186.65) | 38.17 (35.56,39.98) |  | 0.93 (0.84,1.02) | -0.90 (-1.03,-0.77) |
| United States Virgin Islands | 29.24  (24.61,34.31) | 35.69 (30.15,41.92) |  | 52.53  (40.20,67.22) | 30.36 (23.40,38.83) |  | 1.97 (1.56,2.37) | -0.50 (-0.76,-0.23) |

Abbreviations: ASDR, age-standardized DALYs rate; EAPC, estimated annual percentage change; SDI, Sociodemographic Index; UI, uncertainty interval.

**eTable5.** Deaths of CRC Between 1990 and 2021 at the 204 Countries and Territories Level

| Location | Rate per 100,000 (95% UI) | | | | | | | |
| --- | --- | --- | --- | --- | --- | --- | --- | --- |
|  | 1990 |  |  | 2021 |  |  | 1990-2021 |  |
|  | Deaths cases | ASMR |  | Deaths cases | ASMR |  | Cases change | EAPC (95% CI) |
| Countries | | | | | | | | |
| American Samoa | 3.01 (2.56,3.52) | 15.09 (12.86,17.60) |  | 6.76 (5.67,8.31) | 15.48 (12.96,18.89) |  | 2.65 (2.56,2.73) | 0.22 (0.10,0.34) |
| Antigua and Barbuda | 6.87 (6.27,7.50) | 12.23 (11.14,13.38) |  | 14.83 (13.58,16.12) | 14.95 (13.61,16.34) |  | 2.61 (2.40,2.82) | 0.74 (0.55,0.93) |
| Arab Republic of Egypt | 1456.39 (1292.45,1641.91) | 5.65 (4.99,6.47) |  | 5014.05 (4079.17,6134.59) | 8.78 (7.29,10.58) |  | 4.63 (4.41,4.86) | 2.15 (1.84,2.46) |
| Argentine Republic | 6775.37 (6202.43,7379.46) | 21.69 (19.76,23.60) |  | 11066.55 (9747.37,12451.16) | 19.44 (17.08,21.88) |  | 1.88 (1.63,2.14) | -0.06 (-0.28,0.16) |
| Australia | 4603.50 (4165.62,5026.49) | 23.80 (21.56,26.00) |  | 6673.85 (5734.16,7639.43) | 13.98 (12.19,15.90) |  | 0.93 (0.83,1.04) | -1.95 (-2.05,-1.85) |
| Barbados | 56.58 (51.22,62.05) | 18.70 (16.98,20.46) |  | 119.41 (94.16,145.22) | 22.93 (18.10,27.93) |  | 2.67 (2.52,2.82) | 0.95 (0.69,1.21) |
| Belize | 5.88 (5.37,6.47) | 6.35 (5.79,6.98) |  | 26.17 (22.58,30.46) | 9.20 (7.95,10.64) |  | 5.05 (4.69,5.42) | 1.23 (0.73,1.72) |
| Bermuda | 16.82 (15.42,18.41) | 28.26 (25.85,30.92) |  | 26.81 (22.23,32.98) | 18.37 (15.27,22.59) |  | 1.77 (1.51,2.04) | -1.25 (-1.43,-1.07) |
| Bolivarian Republic of Venezuela | 820.43 (753.51,884.19) | 8.81 (8.06,9.50) |  | 2986.53 (2230.55,3874.12) | 10.25 (7.64,13.20) |  | 4.40 (4.27,4.53) | 0.49 (0.38,0.60) |
| Bosnia and Herzegovina | 528.81 (461.17,597.67) | 13.76 (11.94,15.56) |  | 1206.46 (948.49,1493.24) | 18.90 (14.84,23.39) |  | 3.15 (2.82,3.48) | 1.39 (1.19,1.60) |
| Brunei Darussalam | 27.63 (22.03,33.35) | 28.47 (23.21,34.24) |  | 62.35 (52.31,73.53) | 20.16 (17.04,23.91) |  | 3.15 (2.78,3.53) | -0.50 (-0.81,-0.19) |
| Burkina Faso | 197.62 (160.69,246.84) | 5.13 (4.22,6.32) |  | 503.15 (373.42,646.95) | 6.17 (4.70,7.85) |  | 3.32 (3.18,3.46) | 0.81 (0.73,0.89) |
| Canada | 6717.75 (6173.17,7295.85) | 20.69 (18.99,22.45) |  | 10761.41 (9390.39,12097.89) | 14.15 (12.47,15.80) |  | 1.78 (1.67,1.89) | -1.04 (-1.15,-0.93) |
| Central African Republic | 101.29 (67.47,136.33) | 9.58 (6.90,12.86) |  | 188.17 (126.11,271.30) | 9.12 (6.40,12.86) |  | 2.07 (2.01,2.12) | -0.13 (-0.20,-0.07) |
| Commonwealth of Dominica | 7.69 (6.83,8.91) | 13.00 (11.55,14.98) |  | 12.16 (10.32,14.47) | 15.18 (12.81,17.99) |  | 1.60 (1.53,1.66) | 0.61 (0.54,0.68) |
| Commonwealth of the Bahamas | 23.68 (21.37,26.29) | 15.63 (14.14,17.33) |  | 70.88 (57.64,87.33) | 18.47 (15.15,22.54) |  | 3.90 (3.77,4.03) | 0.80 (0.66,0.94) |
| Cook Islands | 0.88 (0.73,1.02) | 7.83 (6.60,9.01) |  | 1.45 (1.17,1.80) | 5.68 (4.59,7.09) |  | 1.50 (1.32,1.68) | -1.19 (-1.35,-1.03) |
| Czech Republic | 4817.20 (4334.01,5388.35) | 34.55 (31.12,38.43) |  | 4526.19 (3801.39,5350.54) | 20.16 (16.98,23.84) |  | -0.37 (-0.50,-0.25) | -2.05 (-2.25,-1.85) |
| Democratic People's Republic of Korea | 1781.81 (1297.05,2432.21) | 11.60 (8.60,15.37) |  | 3412.23 (2275.86,5055.17) | 10.51 (7.05,15.44) |  | 2.28 (2.15,2.41) | -0.16 (-0.26,-0.06) |
| Democratic Republic of Sao Tome and Principe | 4.95 (4.14,5.85) | 8.20 (6.89,9.61) |  | 10.41 (8.57,12.76) | 10.85 (8.98,13.19) |  | 2.58 (2.47,2.69) | 1.13 (1.05,1.20) |
| Democratic Republic of the Congo | 916.82 (714.88,1184.68) | 6.62 (5.17,8.67) |  | 2168.22 (1495.54,3151.07) | 6.59 (4.52,9.67) |  | 2.81 (2.56,3.07) | 0.06 (-0.13,0.25) |
| Democratic Republic of Timor-Leste | 22.50 (15.97,29.94) | 8.55 (6.27,11.09) |  | 76.72 (58.76,100.16) | 9.40 (7.23,12.26) |  | 4.45 (4.23,4.67) | 0.45 (0.26,0.63) |
| Democratic Socialist Republic of Sri Lanka | 492.11 (433.69,558.60) | 4.98 (4.36,5.67) |  | 1184.39 (781.56,1641.80) | 4.52 (3.03,6.20) |  | 3.21 (3.05,3.38) | 0.08 (-0.09,0.25) |
| Dominican Republic | 259.30 (216.05,305.36) | 7.81 (6.50,9.15) |  | 900.32 (701.43,1154.51) | 9.14 (7.10,11.73) |  | 4.45 (4.18,4.72) | 1.02 (0.80,1.23) |
| Eastern Republic of Uruguay | 1017.17 (926.87,1106.18) | 26.06 (23.73,28.35) |  | 1638.53 (1441.10,1850.26) | 27.46 (24.25,30.91) |  | 1.39 (1.28,1.50) | 0.03 (-0.07,0.14) |
| Federal Democratic Republic of Ethiopia | 3870.48 (2168.99,4766.84) | 21.93 (12.86,26.83) |  | 6115.19 (5090.59,7352.38) | 15.91 (13.33,19.01) |  | 1.15 (0.82,1.47) | -1.32 (-1.51,-1.12) |
| Federal Democratic Republic of Nepal | 374.09 (264.34,514.04) | 4.09 (2.99,5.53) |  | 923.38 (682.40,1218.82) | 4.14 (3.09,5.45) |  | 3.21 (2.83,3.59) | 0.14 (-0.21,0.49) |
| Federal Republic of Germany | 34764.44 (31294.37,37867.45) | 26.39 (23.77,28.75) |  | 30916.26 (25821.42,35199.14) | 14.63 (12.49,16.47) |  | -0.57 (-0.68,-0.47) | -2.18 (-2.30,-2.06) |
| Federal Republic of Nigeria | 2119.20 (1655.70,2687.88) | 5.24 (4.14,6.50) |  | 4622.72 (3642.42,5758.22) | 5.78 (4.76,6.96) |  | 2.66 (2.54,2.77) | 0.49 (0.41,0.57) |
| Federal Republic of Somalia | 220.42 (142.38,318.09) | 9.76 (6.56,14.23) |  | 552.67 (356.31,825.82) | 9.92 (6.38,14.54) |  | 3.20 (3.13,3.28) | 0.21 (0.14,0.27) |
| Federated States of Micronesia | 5.84 (4.61,7.48) | 12.79 (10.23,16.20) |  | 8.21 (5.94,11.29) | 12.42 (9.00,16.65) |  | 1.08 (0.94,1.21) | -0.11 (-0.14,-0.07) |
| Federative Republic of Brazil | 7970.04 (7540.27,8369.29) | 9.65 (9.00,10.15) |  | 28820.97 (26426.57,30696.37) | 11.60 (10.60,12.37) |  | 4.30 (4.18,4.42) | 0.63 (0.54,0.72) |
| French Republic | 20853.65 (19035.64,22565.55) | 24.09 (22.02,26.01) |  | 24584.56 (20486.19,28213.83) | 15.12 (12.88,17.10) |  | 0.63 (0.57,0.69) | -1.46 (-1.52,-1.41) |
| Gabonese Republic | 73.72 (44.77,105.15) | 13.58 (8.46,19.35) |  | 129.34 (93.39,169.84) | 13.54 (9.99,17.31) |  | 1.81 (1.70,1.92) | -0.03 (-0.08,0.03) |
| Georgia | 629.36 (555.70,712.14) | 10.15 (8.98,11.44) |  | 795.12 (684.95,908.88) | 13.30 (11.42,15.29) |  | 1.90 (1.42,2.38) | 2.09 (1.59,2.60) |
| Grand Duchy of Luxembourg | 145.25 (134.36,154.41) | 26.74 (24.78,28.40) |  | 166.89 (146.55,185.65) | 14.62 (12.82,16.25) |  | 0.49 (0.41,0.57) | -1.91 (-2.08,-1.74) |
| Greenland | 12.79 (11.25,14.84) | 42.36 (37.22,48.92) |  | 15.56 (13.15,18.70) | 24.60 (20.85,29.54) |  | 0.69 (0.61,0.78) | -1.79 (-1.88,-1.70) |
| Grenada | 10.09 (8.79,11.65) | 13.40 (11.65,15.46) |  | 18.22 (15.36,21.27) | 17.32 (14.67,20.21) |  | 2.24 (2.11,2.38) | 1.07 (0.83,1.31) |
| Guam | 10.17 (8.72,11.56) | 15.78 (13.35,17.94) |  | 21.56 (18.39,24.98) | 10.23 (8.77,11.85) |  | 2.95 (2.67,3.23) | -0.77 (-1.15,-0.38) |
| Hashemite Kingdom of Jordan | 139.15 (113.22,167.47) | 10.87 (9.01,13.11) |  | 594.41 (456.84,799.82) | 8.78 (6.70,11.64) |  | 4.81 (4.65,4.97) | -0.61 (-0.82,-0.40) |
| Hellenic Republic | 2382.21 (2175.42,2587.09) | 15.87 (14.49,17.19) |  | 3877.75 (3361.45,4315.71) | 14.03 (12.29,15.52) |  | 1.34 (1.14,1.53) | -0.75 (-0.96,-0.53) |
| Hungary | 4473.47 (3784.24,5128.72) | 30.77 (26.05,35.12) |  | 5211.45 (4366.55,6242.47) | 26.01 (21.73,31.13) |  | 0.51 (0.28,0.73) | -0.63 (-0.87,-0.39) |
| Independent State of Papua New Guinea | 66.84 (48.01,90.07) | 3.87 (2.88,5.04) |  | 173.28 (132.85,217.79) | 3.56 (2.80,4.41) |  | 3.06 (2.89,3.23) | -0.36 (-0.44,-0.28) |
| Independent State of Samoa | 7.42 (6.03,8.80) | 9.59 (7.79,11.34) |  | 12.61 (10.15,15.49) | 9.35 (7.59,11.42) |  | 1.71 (1.67,1.76) | -0.10 (-0.17,-0.04) |
| Ireland | 1044.49 (968.61,1122.57) | 25.80 (23.92,27.61) |  | 1182.82 (1002.68,1350.66) | 14.38 (12.21,16.40) |  | 0.56 (0.41,0.71) | -1.72 (-1.81,-1.64) |
| Islamic Republic of Afghanistan | 796.88 (303.98,1227.91) | 11.83 (5.00,18.07) |  | 1223.67 (555.46,1914.83) | 12.30 (6.20,18.55) |  | 1.35 (1.16,1.55) | 0.26 (0.19,0.33) |
| Islamic Republic of Iran | 1700.64 (1433.97,1956.30) | 7.25 (6.12,8.34) |  | 5409.91 (4625.88,6012.27) | 7.37 (6.31,8.19) |  | 4.45 (4.21,4.70) | 0.51 (0.30,0.73) |
| Islamic Republic of Mauritania | 59.37 (46.09,72.89) | 6.45 (5.06,7.89) |  | 136.96 (100.65,184.13) | 7.09 (5.18,9.50) |  | 2.84 (2.60,3.08) | 0.35 (0.18,0.52) |
| Islamic Republic of Pakistan | 2704.53 (2282.62,3111.08) | 5.03 (4.21,5.79) |  | 7225.12 (5933.14,9021.82) | 6.29 (5.22,7.80) |  | 2.92 (2.76,3.08) | 0.46 (0.23,0.69) |
| Jamaica | 201.03 (178.56,223.04) | 10.97 (9.74,12.17) |  | 473.52 (365.96,620.30) | 15.09 (11.62,19.81) |  | 2.83 (2.49,3.17) | 1.09 (0.74,1.43) |
| Japan | 31351.31 (29409.63,32588.03) | 19.00 (17.73,19.82) |  | 67923.61 (56450.94,74359.76) | 15.89 (13.97,16.97) |  | 2.50 (2.43,2.57) | -0.64 (-0.70,-0.57) |
| Kingdom of Bahrain | 18.58 (16.28,21.52) | 12.91 (11.35,14.87) |  | 73.88 (58.70,102.17) | 10.98 (8.94,14.46) |  | 4.32 (4.23,4.42) | -0.90 (-1.22,-0.58) |
| Kingdom of Belgium | 4008.35 (3553.11,4376.00) | 25.44 (22.53,27.79) |  | 3675.97 (3103.69,4194.48) | 14.13 (12.20,15.98) |  | -0.10 (-0.21,0.02) | -1.80 (-1.90,-1.71) |
| Kingdom of Bhutan | 10.70 (6.67,14.72) | 4.50 (2.79,6.04) |  | 26.00 (19.58,33.67) | 4.41 (3.31,5.68) |  | 2.93 (2.83,3.02) | -0.08 (-0.19,0.03) |
| Kingdom of Cambodia | 561.16 (365.16,741.87) | 12.81 (8.71,16.66) |  | 1674.41 (1249.72,2175.54) | 14.37 (10.80,18.37) |  | 3.69 (3.56,3.82) | 0.37 (0.30,0.45) |
| Kingdom of Denmark | 1972.66 (1779.76,2160.91) | 23.56 (21.31,25.78) |  | 2449.27 (2114.31,2760.51) | 18.97 (16.41,21.34) |  | 0.27 (0.04,0.51) | -1.07 (-1.36,-0.78) |
| Kingdom of Eswatini | 30.37 (21.07,39.10) | 11.74 (8.17,15.06) |  | 79.84 (51.53,109.16) | 15.24 (10.05,20.14) |  | 3.42 (2.80,4.03) | 1.26 (0.75,1.76) |
| Kingdom of Lesotho | 54.25 (41.49,69.81) | 6.92 (5.28,8.89) |  | 134.60 (94.17,192.30) | 13.47 (9.64,19.03) |  | 3.58 (3.05,4.11) | 2.96 (2.51,3.41) |
| Kingdom of Morocco | 823.33 (644.87,1008.67) | 5.96 (4.68,7.29) |  | 2724.92 (1991.94,3532.30) | 8.33 (6.20,10.64) |  | 4.20 (4.05,4.35) | 1.32 (1.21,1.43) |
| Kingdom of Norway | 1716.73 (1571.72,1804.74) | 24.02 (22.17,25.21) |  | 1989.41 (1719.65,2160.23) | 18.12 (15.88,19.54) |  | 0.34 (0.23,0.45) | -0.98 (-1.07,-0.89) |
| Kingdom of Saudi Arabia | 329.02 (238.24,431.91) | 5.82 (4.29,7.52) |  | 1449.74 (1135.07,1816.18) | 7.64 (6.29,9.36) |  | 4.97 (4.72,5.22) | 1.08 (0.72,1.44) |
| Kingdom of Spain | 10879.42 (9883.13,11753.33) | 19.99 (18.12,21.63) |  | 18173.54 (15272.12,20682.09) | 16.59 (14.25,18.69) |  | 1.86 (1.66,2.05) | -0.50 (-0.63,-0.37) |
| Kingdom of Sweden | 2985.00 (2675.13,3283.01) | 18.83 (16.94,20.69) |  | 3490.15 (2847.02,4096.71) | 14.37 (11.88,16.75) |  | 0.51 (0.36,0.65) | -0.78 (-0.94,-0.61) |
| Kingdom of Thailand | 4387.70 (3691.69,5126.96) | 13.23 (11.05,15.50) |  | 15991.04 (12224.09,20388.19) | 14.89 (11.43,18.95) |  | 4.01 (3.89,4.13) | 0.11 (-0.02,0.24) |
| Kingdom of the Netherlands | 4876.74 (4495.25,5263.63) | 23.94 (22.16,25.80) |  | 7444.83 (6510.57,8299.48) | 19.99 (17.63,22.18) |  | 1.47 (1.33,1.62) | -0.50 (-0.68,-0.32) |
| Kingdom of Tonga | 3.51 (2.88,4.20) | 6.95 (5.68,8.30) |  | 5.62 (4.40,7.11) | 7.22 (5.65,9.12) |  | 1.47 (1.29,1.64) | 0.13 (0.01,0.25) |
| Kyrgyz Republic | 323.28 (293.53,354.79) | 10.93 (9.94,11.98) |  | 325.83 (265.78,394.07) | 7.03 (5.66,8.56) |  | 0.09 (-0.22,0.39) | -1.18 (-1.39,-0.97) |
| Lao People's Democratic Republic | 269.61 (145.27,372.03) | 13.40 (7.76,18.13) |  | 572.35 (407.38,755.48) | 13.03 (9.55,16.89) |  | 2.32 (2.25,2.40) | -0.17 (-0.20,-0.13) |
| Lebanese Republic | 268.24 (182.76,335.12) | 13.53 (9.38,16.62) |  | 695.35 (565.76,884.20) | 10.99 (8.98,14.02) |  | 3.84 (3.54,4.14) | -0.21 (-0.45,0.03) |
| Malaysia | 1463.59 (1281.96,1655.94) | 16.50 (14.34,18.70) |  | 4777.00 (4147.79,5341.31) | 18.01 (15.58,20.27) |  | 3.77 (3.60,3.95) | 0.10 (-0.04,0.23) |
| Mongolia | 81.03 (63.08,103.00) | 7.67 (5.96,9.76) |  | 179.50 (140.26,221.57) | 8.12 (6.35,10.04) |  | 2.53 (2.23,2.84) | 0.02 (-0.12,0.16) |
| Montenegro | 85.14 (73.40,99.52) | 13.96 (12.04,16.24) |  | 168.12 (140.33,200.16) | 17.74 (14.86,20.97) |  | 2.45 (2.33,2.57) | 0.90 (0.77,1.04) |
| New Zealand | 1174.26 (1059.62,1296.00) | 30.27 (27.28,33.43) |  | 1601.92 (1378.46,1824.10) | 18.15 (15.68,20.59) |  | 0.92 (0.82,1.03) | -1.71 (-1.80,-1.62) |
| North Macedonia | 285.30 (246.80,329.49) | 16.07 (13.90,18.67) |  | 588.56 (467.87,713.19) | 19.26 (15.65,23.16) |  | 2.55 (2.26,2.85) | 0.61 (0.25,0.97) |
| Northern Mariana Islands | 2.37 (1.86,3.10) | 15.62 (12.61,19.56) |  | 7.00 (5.98,7.80) | 15.57 (13.29,17.46) |  | 3.81 (3.64,3.98) | -0.00 (-0.23,0.23) |
| Palestine | 147.42 (111.41,188.40) | 18.27 (14.03,23.16) |  | 340.35 (282.20,400.86) | 15.16 (12.68,17.71) |  | 2.84 (2.74,2.95) | -0.54 (-0.71,-0.38) |
| People's Democratic Republic of Algeria | 513.43 (426.23,611.61) | 5.34 (4.42,6.28) |  | 1434.92 (1126.29,1804.44) | 4.80 (3.78,5.99) |  | 3.50 (3.36,3.65) | 0.03 (-0.15,0.21) |
| People's Republic of Bangladesh | 2129.49 (1644.68,2777.34) | 4.56 (3.52,5.89) |  | 4750.66 (3543.84,6607.47) | 3.58 (2.71,4.89) |  | 2.81 (2.59,3.04) | -0.85 (-1.00,-0.70) |
| People's Republic of China | 119303.50 (102706.42,137153.13) | 15.49 (13.43,17.70) |  | 275129.23 (223378.58,330960.39) | 13.64 (11.09,16.31) |  | 2.69 (2.58,2.79) | -0.49 (-0.55,-0.43) |
| Plurinational State of Bolivia | 396.46 (252.68,534.39) | 13.44 (8.72,18.07) |  | 1177.50 (795.45,1633.75) | 14.04 (9.59,19.38) |  | 3.82 (3.74,3.90) | 0.19 (0.16,0.22) |
| Portuguese Republic | 3109.02 (2758.30,3465.71) | 23.31 (20.58,25.91) |  | 4685.60 (3967.70,5321.41) | 17.32 (14.85,19.68) |  | 1.49 (1.29,1.68) | -0.88 (-1.07,-0.70) |
| Principality of Andorra | 13.48 (9.75,18.94) | 24.65 (17.89,34.55) |  | 25.61 (18.43,33.97) | 15.91 (11.45,21.20) |  | 2.02 (1.76,2.27) | -1.11 (-1.37,-0.85) |
| Principality of Monaco | 18.11 (13.92,21.81) | 24.09 (18.62,29.14) |  | 27.00 (21.54,32.49) | 25.23 (20.13,30.60) |  | 1.34 (1.22,1.47) | 0.26 (0.16,0.37) |
| Puerto Rico | 509.83 (464.01,556.73) | 14.49 (13.16,15.82) |  | 1003.12 (836.00,1205.71) | 13.48 (11.26,16.21) |  | 2.14 (1.84,2.43) | -0.27 (-0.48,-0.06) |
| Republic of Albania | 144.37 (121.82,172.40) | 7.70 (6.50,9.17) |  | 325.63 (251.90,426.13) | 7.52 (5.79,9.81) |  | 3.09 (2.87,3.31) | 0.25 (0.03,0.48) |
| Republic of Angola | 281.48 (204.21,367.41) | 7.91 (5.87,10.02) |  | 903.96 (658.86,1204.54) | 8.53 (6.26,11.21) |  | 3.96 (3.74,4.19) | 0.27 (0.17,0.38) |
| Republic of Armenia | 364.67 (332.82,394.69) | 13.62 (12.34,14.82) |  | 568.92 (497.76,654.08) | 13.12 (11.50,15.07) |  | 1.84 (1.65,2.02) | 0.26 (0.05,0.48) |
| Republic of Austria | 3022.68 (2743.73,3255.64) | 24.74 (22.48,26.68) |  | 2345.63 (2014.38,2702.93) | 11.80 (10.26,13.52) |  | -0.76 (-0.82,-0.69) | -2.42 (-2.48,-2.37) |
| Republic of Azerbaijan | 444.30 (374.16,510.22) | 8.87 (7.48,10.17) |  | 713.02 (540.50,902.75) | 7.18 (5.46,8.98) |  | 1.83 (1.49,2.17) | -0.26 (-0.55,0.03) |
| Republic of Belarus | 2091.11 (1826.90,2374.80) | 16.19 (14.15,18.39) |  | 2814.28 (2146.98,3530.81) | 17.32 (13.20,21.73) |  | 0.26 (-0.07,0.59) | -0.47 (-0.78,-0.15) |
| Republic of Benin | 93.26 (78.98,110.14) | 4.98 (4.23,5.85) |  | 258.03 (196.98,338.06) | 5.66 (4.39,7.17) |  | 3.56 (3.42,3.71) | 0.61 (0.51,0.70) |
| Republic of Botswana | 48.72 (35.81,63.01) | 9.91 (7.38,12.64) |  | 130.18 (96.84,178.28) | 10.08 (7.76,13.38) |  | 3.16 (2.95,3.37) | 0.14 (-0.07,0.35) |
| Republic of Bulgaria | 2505.70 (2202.77,2862.94) | 21.35 (18.84,24.22) |  | 3744.05 (3049.42,4471.98) | 25.71 (20.96,30.73) |  | 1.76 (1.56,1.96) | 1.05 (0.85,1.25) |
| Republic of Burundi | 211.16 (161.75,268.40) | 9.53 (7.39,11.91) |  | 362.82 (258.86,515.40) | 8.33 (6.11,11.45) |  | 1.54 (1.15,1.94) | -0.71 (-0.86,-0.57) |
| Republic of Cabo Verde | 8.50 (7.18,9.86) | 3.58 (3.02,4.13) |  | 37.92 (29.45,45.03) | 9.12 (7.01,10.81) |  | 4.52 (4.10,4.94) | 2.49 (2.06,2.92) |
| Republic of Cameroon | 258.37 (218.31,305.50) | 6.54 (5.57,7.70) |  | 821.26 (569.82,1108.25) | 7.48 (5.44,9.94) |  | 3.99 (3.91,4.07) | 0.58 (0.50,0.67) |
| Republic of Chad | 113.08 (91.84,142.46) | 4.27 (3.47,5.38) |  | 323.74 (241.01,424.01) | 6.29 (4.79,8.11) |  | 3.70 (3.63,3.77) | 1.50 (1.37,1.62) |
| Republic of Chile | 1180.74 (1079.53,1285.91) | 12.50 (11.36,13.65) |  | 3411.04 (2988.85,3840.84) | 13.13 (11.54,14.80) |  | 3.83 (3.69,3.97) | 0.47 (0.35,0.59) |
| Republic of Colombia | 1561.35 (1426.32,1689.33) | 9.43 (8.56,10.28) |  | 5783.34 (4770.34,6998.40) | 10.49 (8.66,12.68) |  | 4.22 (4.10,4.35) | 0.19 (0.04,0.35) |
| Republic of Costa Rica | 161.51 (145.54,177.57) | 9.49 (8.55,10.44) |  | 831.51 (719.04,959.64) | 15.13 (13.09,17.48) |  | 5.75 (5.54,5.96) | 1.80 (1.60,2.00) |
| Republic of Croatia | 141.05 (113.02,169.63) | 4.06 (3.32,4.84) |  | 397.35 (292.12,555.69) | 4.00 (3.06,5.39) |  | 3.23 (3.06,3.39) | -0.08 (-0.18,0.02) |
| Republic of Cuba | 1377.41 (1205.74,1549.83) | 24.31 (21.24,27.30) |  | 2309.00 (1964.96,2666.81) | 24.29 (20.78,27.97) |  | 1.90 (1.67,2.13) | 0.15 (-0.03,0.33) |
| Republic of Cyprus | 1586.74 (1454.06,1714.85) | 15.77 (14.45,17.04) |  | 3528.22 (3009.47,4078.64) | 17.47 (14.89,20.22) |  | 2.78 (2.67,2.88) | 0.45 (0.34,0.56) |
| Republic of C么te d'Ivoire | 129.60 (107.53,155.12) | 19.97 (16.45,23.81) |  | 248.67 (207.47,295.77) | 12.75 (10.76,14.93) |  | 2.46 (2.32,2.61) | -1.12 (-1.28,-0.96) |
| Republic of Djibouti | 11.72 (8.05,16.60) | 9.54 (6.83,13.41) |  | 69.73 (48.99,98.93) | 12.66 (9.13,17.12) |  | 5.98 (5.88,6.07) | 0.96 (0.89,1.04) |
| Republic of Ecuador | 360.69 (328.73,392.30) | 7.34 (6.68,7.96) |  | 1501.89 (1175.80,1898.14) | 9.46 (7.42,11.88) |  | 5.19 (4.82,5.57) | 1.23 (0.90,1.56) |
| Republic of El Salvador | 151.87 (138.75,166.23) | 5.12 (4.67,5.62) |  | 513.17 (413.92,626.01) | 8.11 (6.54,9.90) |  | 4.07 (3.86,4.29) | 1.52 (1.35,1.70) |
| Republic of Equatorial Guinea | 15.43 (10.96,20.05) | 8.55 (6.33,11.02) |  | 48.68 (32.79,68.91) | 10.49 (7.24,14.52) |  | 4.17 (3.86,4.48) | 0.88 (0.74,1.03) |
| Republic of Estonia | 378.03 (346.91,412.26) | 18.40 (16.88,20.14) |  | 529.22 (433.46,622.46) | 17.80 (14.52,20.87) |  | 0.92 (0.72,1.13) | -0.41 (-0.57,-0.25) |
| Republic of Fiji | 29.24 (23.48,35.20) | 8.90 (7.13,10.61) |  | 65.90 (48.50,86.20) | 9.72 (7.29,12.58) |  | 2.89 (2.64,3.15) | 0.28 (0.09,0.47) |
| Republic of Finland | 1085.22 (988.83,1167.93) | 14.97 (13.63,16.14) |  | 1582.16 (1352.82,1796.18) | 11.32 (9.78,12.77) |  | 1.26 (1.08,1.44) | -0.90 (-1.03,-0.78) |
| Republic of Ghana | 286.23 (224.50,360.51) | 5.23 (4.17,6.56) |  | 990.10 (769.45,1238.70) | 6.89 (5.39,8.51) |  | 4.32 (4.24,4.39) | 1.11 (1.04,1.18) |
| Republic of Guatemala | 146.91 (135.43,159.77) | 4.85 (4.49,5.26) |  | 739.72 (634.83,861.87) | 6.93 (5.98,8.05) |  | 5.45 (5.13,5.78) | 0.97 (0.63,1.31) |
| Republic of Guinea | 128.09 (103.72,156.92) | 4.09 (3.32,4.99) |  | 247.54 (182.36,336.97) | 4.69 (3.52,6.25) |  | 2.09 (1.87,2.30) | 0.57 (0.48,0.66) |
| Republic of Guinea-Bissau | 27.00 (17.12,34.67) | 7.38 (4.84,9.29) |  | 53.15 (39.65,67.62) | 8.39 (6.31,10.47) |  | 2.45 (2.31,2.59) | 0.64 (0.56,0.72) |
| Republic of Guyana | 40.88 (36.10,45.90) | 11.39 (10.05,12.74) |  | 80.25 (60.84,102.93) | 13.25 (10.17,16.90) |  | 2.66 (2.42,2.91) | 0.76 (0.48,1.03) |
| Republic of Haiti | 436.16 (281.39,574.30) | 14.98 (10.17,19.46) |  | 899.36 (612.35,1276.58) | 13.83 (9.56,19.25) |  | 2.55 (2.47,2.63) | -0.09 (-0.16,-0.02) |
| Republic of Honduras | 83.35 (70.87,98.16) | 4.14 (3.51,4.88) |  | 375.39 (295.94,489.42) | 6.27 (4.99,8.15) |  | 5.22 (5.05,5.39) | 1.50 (1.32,1.69) |
| Republic of Iceland | 46.59 (42.06,50.91) | 15.84 (14.39,17.33) |  | 69.58 (57.61,81.38) | 11.19 (9.38,13.03) |  | 1.43 (1.31,1.55) | -1.02 (-1.15,-0.88) |
| Republic of India | 20062.43 (17078.75,22807.51) | 4.31 (3.62,4.94) |  | 54017.54 (48106.10,61278.99) | 4.60 (4.09,5.21) |  | 3.18 (3.05,3.32) | 0.11 (-0.02,0.25) |
| Republic of Indonesia | 8744.91 (6814.40,10597.23) | 9.21 (7.19,11.05) |  | 27107.60 (20431.05,33822.21) | 12.39 (9.50,15.30) |  | 3.63 (3.56,3.70) | 0.98 (0.89,1.07) |
| Republic of Iraq | 421.07 (340.04,515.22) | 5.25 (4.25,6.48) |  | 1413.11 (1070.75,1769.28) | 6.33 (4.84,7.83) |  | 4.19 (3.94,4.45) | 0.64 (0.42,0.85) |
| Republic of Italy | 17782.72 (16468.59,18611.50) | 19.85 (18.35,20.79) |  | 23428.64 (19953.58,25626.73) | 14.28 (12.57,15.43) |  | 0.92 (0.80,1.03) | -1.10 (-1.22,-0.98) |
| Republic of Kazakhstan | 1929.68 (1696.22,2162.97) | 15.43 (13.51,17.36) |  | 1818.57 (1545.51,2116.50) | 10.53 (8.90,12.23) |  | 0.07 (-0.09,0.24) | -0.91 (-1.16,-0.66) |
| Republic of Kenya | 375.34 (303.85,454.14) | 4.70 (3.82,5.68) |  | 1519.83 (1251.17,1905.95) | 7.18 (5.91,8.86) |  | 5.08 (4.87,5.29) | 1.87 (1.63,2.11) |
| Republic of Kiribati | 3.06 (2.49,3.81) | 8.87 (7.20,11.28) |  | 5.91 (4.50,7.87) | 9.00 (7.25,11.87) |  | 2.09 (2.06,2.11) | -0.08 (-0.16,0.00) |
| Republic of Korea | 3800.39 (3301.76,4331.93) | 14.12 (12.18,15.99) |  | 11660.28 (9597.58,13743.91) | 12.56 (10.32,14.83) |  | 3.62 (3.51,3.72) | -0.54 (-0.70,-0.38) |
| Republic of Latvia | 638.06 (569.13,717.47) | 17.73 (15.82,19.99) |  | 703.78 (576.22,840.77) | 16.66 (13.60,19.90) |  | 0.53 (0.35,0.71) | -0.11 (-0.28,0.06) |
| Republic of Liberia | 53.67 (43.10,66.74) | 5.11 (4.13,6.30) |  | 103.46 (64.50,155.77) | 5.58 (3.60,8.29) |  | 2.44 (2.14,2.75) | 0.63 (0.41,0.84) |
| Republic of Lithuania | 744.06 (684.87,806.78) | 16.50 (15.17,17.95) |  | 1041.58 (857.66,1235.40) | 16.92 (13.90,19.95) |  | 1.17 (0.99,1.35) | 0.08 (-0.08,0.23) |
| Republic of Madagascar | 390.98 (315.66,473.47) | 8.13 (6.64,9.82) |  | 817.60 (590.35,1070.80) | 8.12 (5.92,10.46) |  | 2.58 (2.28,2.88) | 0.11 (-0.00,0.23) |
| Republic of Malawi | 143.90 (118.60,176.47) | 4.06 (3.38,4.93) |  | 312.82 (242.79,428.88) | 4.55 (3.57,6.08) |  | 2.31 (2.19,2.43) | 0.23 (0.06,0.40) |
| Republic of Maldives | 6.03 (3.53,7.62) | 7.35 (4.88,9.20) |  | 13.50 (10.95,16.70) | 4.37 (3.53,5.34) |  | 2.29 (2.12,2.46) | -2.01 (-2.13,-1.89) |
| Republic of Mali | 250.71 (214.73,293.94) | 6.98 (6.00,8.10) |  | 564.12 (431.95,715.62) | 7.10 (5.55,8.96) |  | 3.10 (2.85,3.36) | 0.28 (0.18,0.39) |
| Republic of Malta | 78.51 (69.46,88.22) | 18.91 (16.72,21.21) |  | 142.59 (118.05,164.22) | 13.62 (11.31,15.67) |  | 1.89 (1.76,2.01) | -1.12 (-1.23,-1.01) |
| Republic of Mauritius | 56.72 (53.03,60.86) | 8.35 (7.77,8.98) |  | 254.32 (232.73,272.98) | 14.25 (12.99,15.31) |  | 4.40 (4.19,4.60) | 1.18 (0.98,1.38) |
| Republic of Moldova | 769.86 (688.44,842.37) | 18.02 (16.23,19.63) |  | 1090.21 (922.45,1280.06) | 18.07 (15.29,21.24) |  | 1.56 (1.10,2.02) | 0.46 (0.00,0.93) |
| Republic of Mozambique | 155.97 (129.67,183.26) | 3.26 (2.74,3.80) |  | 365.54 (273.77,463.63) | 4.15 (3.15,5.18) |  | 3.26 (3.10,3.42) | 1.25 (1.09,1.42) |
| Republic of Namibia | 33.54 (27.85,39.32) | 5.57 (4.65,6.58) |  | 83.20 (61.01,107.70) | 6.37 (4.83,8.03) |  | 2.80 (2.54,3.05) | 0.38 (0.15,0.61) |
| Republic of Nauru | 0.77 (0.44,1.08) | 18.42 (11.62,25.15) |  | 0.91 (0.55,1.22) | 16.98 (10.74,22.30) |  | 0.28 (0.18,0.37) | -0.32 (-0.37,-0.28) |
| Republic of Nicaragua | 68.06 (59.74,77.77) | 4.56 (3.97,5.23) |  | 279.28 (227.51,339.31) | 5.89 (4.81,7.11) |  | 5.05 (4.72,5.37) | 1.27 (0.98,1.56) |
| Republic of Niue | 0.26 (0.22,0.32) | 11.42 (9.40,13.71) |  | 0.25 (0.20,0.30) | 12.03 (9.73,14.51) |  | -0.24 (-0.32,-0.16) | 0.08 (0.05,0.11) |
| Republic of Palau | 1.31 (1.01,1.70) | 15.16 (11.65,19.44) |  | 2.45 (1.86,3.06) | 13.55 (10.47,16.95) |  | 1.83 (1.75,1.91) | -0.20 (-0.30,-0.10) |
| Republic of Panama | 124.72 (113.97,135.08) | 8.59 (7.83,9.32) |  | 495.25 (382.55,602.09) | 11.12 (8.57,13.52) |  | 4.84 (4.73,4.94) | 1.08 (0.99,1.17) |
| Republic of Paraguay | 132.15 (112.75,156.14) | 6.18 (5.26,7.25) |  | 593.60 (457.70,750.14) | 10.57 (8.18,13.31) |  | 5.26 (5.04,5.48) | 2.07 (1.89,2.25) |
| Republic of Peru | 965.42 (811.49,1121.46) | 8.41 (7.06,9.76) |  | 3098.45 (2310.23,3988.14) | 9.32 (6.95,12.00) |  | 3.97 (3.79,4.15) | 0.26 (0.07,0.45) |
| Republic of Poland | 9310.97 (8883.69,9685.18) | 21.59 (20.56,22.48) |  | 18179.61 (16286.87,19763.10) | 24.35 (21.86,26.45) |  | 2.13 (2.03,2.24) | 0.19 (0.03,0.36) |
| Republic of Rwanda | 294.54 (230.37,364.29) | 11.01 (8.63,13.49) |  | 529.55 (371.56,715.96) | 9.49 (6.69,12.68) |  | 1.56 (1.01,2.11) | -1.14 (-1.43,-0.85) |
| Republic of San Marino | 8.72 (7.18,10.41) | 23.74 (19.60,28.33) |  | 9.59 (6.58,13.09) | 11.13 (7.46,15.39) |  | 1.26 (0.80,1.71) | -1.51 (-1.87,-1.15) |
| Republic of Senegal | 163.65 (136.22,197.59) | 5.54 (4.63,6.69) |  | 476.54 (363.23,626.52) | 6.90 (5.27,8.92) |  | 3.78 (3.63,3.93) | 0.87 (0.73,1.02) |
| Republic of Serbia | 2476.08 (2060.86,3014.48) | 25.06 (21.03,30.47) |  | 3628.29 (2953.33,4402.69) | 21.32 (17.31,25.92) |  | 1.20 (1.03,1.37) | -0.59 (-0.68,-0.49) |
| Republic of Seychelles | 9.30 (8.29,10.55) | 16.47 (14.69,18.66) |  | 21.83 (18.81,25.07) | 19.96 (17.19,22.88) |  | 2.94 (2.76,3.12) | 0.75 (0.51,1.00) |
| Republic of Sierra Leone | 86.01 (66.47,110.71) | 4.51 (3.51,5.71) |  | 182.21 (134.44,245.30) | 5.36 (4.03,7.11) |  | 2.73 (2.50,2.96) | 0.91 (0.77,1.05) |
| Republic of Singapore | 456.39 (415.68,503.20) | 22.08 (20.03,24.41) |  | 1044.35 (903.73,1185.52) | 12.43 (10.73,14.16) |  | 2.55 (2.26,2.84) | -2.03 (-2.23,-1.82) |
| Republic of Slovenia | 531.51 (471.56,592.21) | 21.42 (18.96,23.94) |  | 784.85 (630.82,956.26) | 16.18 (13.05,19.72) |  | 1.22 (0.98,1.47) | -1.09 (-1.39,-0.80) |
| Republic of South Africa | 1682.10 (1463.84,2145.01) | 8.56 (7.40,11.07) |  | 4827.80 (4348.86,5384.35) | 11.19 (10.02,12.42) |  | 3.53 (3.27,3.80) | 0.94 (0.68,1.21) |
| Republic of South Sudan | 233.94 (159.28,332.82) | 9.67 (6.67,13.51) |  | 404.06 (281.84,537.43) | 11.53 (8.11,15.14) |  | 1.87 (1.63,2.11) | 0.67 (0.56,0.78) |
| Republic of Sudan | 576.06 (395.21,814.58) | 6.38 (4.59,8.88) |  | 1328.47 (887.11,1916.77) | 6.95 (4.73,9.80) |  | 2.69 (2.59,2.79) | 0.37 (0.30,0.44) |
| Republic of Suriname | 30.86 (27.65,34.22) | 12.71 (11.41,14.09) |  | 85.19 (64.71,107.31) | 13.84 (10.52,17.44) |  | 3.85 (3.62,4.08) | 0.63 (0.42,0.84) |
| Republic of Tajikistan | 213.73 (178.95,252.47) | 7.76 (6.52,9.15) |  | 266.60 (201.87,345.52) | 4.69 (3.63,5.88) |  | 0.66 (0.29,1.03) | -1.58 (-1.80,-1.35) |
| Republic of the Congo | 105.21 (75.85,134.56) | 10.76 (8.20,13.49) |  | 248.39 (190.90,317.89) | 10.10 (8.03,12.53) |  | 2.71 (2.50,2.93) | -0.29 (-0.41,-0.18) |
| Republic of the Gambia | 8.52 (6.68,10.53) | 2.68 (2.15,3.29) |  | 27.70 (20.99,35.92) | 3.05 (2.34,3.93) |  | 3.85 (3.67,4.03) | 0.35 (0.23,0.47) |
| Republic of the Marshall Islands | 1.77 (1.37,2.13) | 11.69 (9.23,13.90) |  | 3.78 (2.74,5.00) | 12.01 (9.12,15.47) |  | 2.56 (2.47,2.65) | 0.10 (0.04,0.17) |
| Republic of the Niger | 110.24 (84.01,141.84) | 4.55 (3.51,5.83) |  | 352.36 (250.74,477.71) | 5.05 (3.66,6.74) |  | 4.09 (3.93,4.24) | 0.57 (0.47,0.68) |
| Republic of the Philippines | 2929.22 (2624.04,3416.57) | 10.30 (9.16,12.34) |  | 10582.32 (8917.80,12374.70) | 13.18 (11.18,15.35) |  | 4.42 (4.35,4.49) | 1.06 (0.98,1.13) |
| Republic of the Union of Myanmar | 2592.30 (1638.64,3708.56) | 11.48 (7.45,16.05) |  | 5371.63 (3958.44,6915.91) | 11.56 (8.58,14.83) |  | 2.23 (2.15,2.30) | -0.08 (-0.15,-0.01) |
| Republic of Trinidad and Tobago | 121.53 (111.89,131.15) | 15.79 (14.52,17.07) |  | 277.62 (212.32,353.79) | 14.56 (11.18,18.53) |  | 2.68 (2.53,2.83) | -0.22 (-0.35,-0.09) |
| Republic of Tunisia | 358.76 (293.21,429.57) | 7.94 (6.52,9.50) |  | 955.75 (695.22,1283.36) | 7.52 (5.47,10.00) |  | 2.99 (2.88,3.09) | -0.35 (-0.42,-0.27) |
| Republic of Turkey | 5512.26 (4692.52,6452.92) | 16.72 (14.33,19.58) |  | 11634.91 (9355.71,14230.96) | 12.93 (10.42,15.75) |  | 2.49 (2.15,2.82) | -0.82 (-1.17,-0.46) |
| Republic of Uganda | 597.59 (486.55,740.69) | 10.09 (8.30,12.34) |  | 1454.33 (1110.16,1938.80) | 10.64 (8.29,13.83) |  | 2.37 (2.15,2.59) | -0.32 (-0.55,-0.09) |
| Republic of Uzbekistan | 721.53 (632.44,813.58) | 6.23 (5.40,7.04) |  | 1272.43 (1022.82,1564.17) | 4.82 (3.86,5.91) |  | 1.84 (1.30,2.39) | -0.73 (-1.15,-0.30) |
| Republic of Vanuatu | 5.79 (4.17,7.58) | 10.30 (7.62,13.23) |  | 16.27 (12.42,20.98) | 10.23 (7.90,13.11) |  | 3.28 (3.17,3.39) | -0.10 (-0.16,-0.03) |
| Republic of Yemen | 310.86 (214.23,432.36) | 6.71 (4.92,9.03) |  | 924.93 (609.81,1351.80) | 6.91 (4.55,9.83) |  | 3.71 (3.63,3.79) | 0.16 (0.10,0.23) |
| Republic of Zambia | 265.44 (217.26,322.08) | 9.93 (8.20,12.00) |  | 880.83 (551.45,1662.65) | 13.54 (8.97,23.41) |  | 3.95 (3.70,4.20) | 0.99 (0.90,1.08) |
| Republic of Zimbabwe | 376.29 (311.45,450.15) | 10.52 (8.72,12.45) |  | 874.01 (667.78,1115.57) | 13.76 (10.88,17.01) |  | 2.79 (2.32,3.26) | 1.24 (0.79,1.70) |
| Romania | 3447.78 (3011.17,3902.24) | 12.62 (11.02,14.32) |  | 8029.06 (6763.91,9501.66) | 21.09 (17.92,24.89) |  | 2.58 (2.34,2.82) | 1.42 (1.19,1.64) |
| Russian Federation | 30863.94 (29835.48,31741.35) | 17.31 (16.69,17.81) |  | 45875.04 (42132.80,49665.84) | 18.99 (17.47,20.56) |  | 1.03 (0.88,1.19) | 0.12 (-0.04,0.28) |
| Saint Kitts and Nevis | 6.36 (5.73,7.02) | 17.05 (15.42,18.75) |  | 10.41 (8.69,12.27) | 17.13 (14.45,19.91) |  | 2.03 (1.77,2.29) | 0.54 (0.37,0.71) |
| Saint Lucia | 10.01 (9.18,10.91) | 12.46 (11.49,13.56) |  | 25.89 (21.30,31.27) | 11.03 (9.08,13.30) |  | 3.00 (2.74,3.26) | -0.78 (-1.01,-0.55) |
| Saint Vincent and the Grenadines | 8.07 (7.36,8.83) | 11.73 (10.70,12.85) |  | 17.99 (15.83,20.51) | 13.23 (11.65,15.05) |  | 2.77 (2.58,2.97) | 0.44 (0.29,0.59) |
| Slovak Republic | 1622.73 (1412.92,1858.90) | 27.09 (23.56,30.98) |  | 2387.61 (1944.52,2850.03) | 24.71 (20.25,29.54) |  | 1.28 (1.22,1.34) | -0.30 (-0.39,-0.20) |
| Socialist Republic of Viet Nam | 3206.25 (2571.52,3980.60) | 8.13 (6.55,10.00) |  | 11681.72 (9004.13,14150.54) | 12.12 (9.45,14.63) |  | 4.44 (4.36,4.52) | 1.46 (1.33,1.59) |
| Solomon Islands | 11.68 (6.67,16.93) | 9.50 (6.12,13.21) |  | 31.37 (22.27,42.04) | 9.73 (7.09,12.79) |  | 3.17 (3.06,3.29) | 0.07 (-0.05,0.19) |
| State of Eritrea | 106.80 (82.47,131.24) | 10.00 (7.86,12.21) |  | 292.05 (214.58,393.11) | 11.82 (8.89,15.28) |  | 3.66 (3.52,3.80) | 0.58 (0.49,0.67) |
| State of Israel | 1022.91 (907.28,1140.98) | 21.63 (19.24,24.02) |  | 1760.94 (1422.90,2050.95) | 13.35 (10.94,15.48) |  | 1.10 (0.76,1.44) | -2.13 (-2.45,-1.81) |
| State of Kuwait | 32.47 (29.17,36.14) | 6.00 (5.33,6.65) |  | 230.40 (185.06,287.05) | 8.67 (7.06,10.72) |  | 6.81 (6.39,7.23) | 1.64 (1.15,2.13) |
| State of Libya | 243.12 (188.18,310.78) | 13.54 (10.43,17.20) |  | 687.38 (503.82,917.84) | 13.95 (10.27,18.59) |  | 3.79 (3.64,3.94) | 0.50 (0.32,0.69) |
| State of Qatar | 12.68 (10.51,15.41) | 14.65 (12.09,17.70) |  | 78.44 (57.79,107.58) | 11.38 (8.70,15.34) |  | 7.08 (6.66,7.50) | -0.62 (-1.31,0.07) |
| Sultanate of Oman | 26.61 (19.49,35.21) | 4.18 (3.11,5.48) |  | 62.16 (47.22,80.93) | 3.65 (2.85,4.55) |  | 2.96 (2.83,3.09) | -0.01 (-0.24,0.22) |
| Swiss Confederation | 1625.80 (1454.72,1773.26) | 14.98 (13.47,16.29) |  | 2091.88 (1710.43,2436.63) | 10.36 (8.65,12.00) |  | 0.72 (0.58,0.87) | -1.29 (-1.46,-1.12) |
| Syrian Arab Republic | 338.43 (267.86,419.32) | 6.64 (5.28,8.19) |  | 756.41 (549.40,1037.68) | 6.41 (4.73,8.55) |  | 2.67 (2.47,2.87) | -0.22 (-0.37,-0.07) |
| Taiwan (Province of China) | 2552.73 (2339.19,2761.99) | 17.02 (15.51,18.43) |  | 9338.54 (8058.98,10547.24) | 21.86 (18.95,24.57) |  | 3.99 (3.67,4.31) | 0.48 (0.15,0.80) |
| Togolese Republic | 52.17 (41.67,65.91) | 4.74 (3.83,5.96) |  | 203.60 (134.98,284.20) | 6.26 (4.33,8.62) |  | 4.66 (4.58,4.74) | 1.06 (0.98,1.14) |
| Tokelau | 0.14 (0.11,0.18) | 11.08 (8.75,13.59) |  | 0.15 (0.11,0.19) | 10.03 (7.75,12.86) |  | 0.07 (0.03,0.10) | -0.33 (-0.35,-0.31) |
| Turkmenistan | 123.73 (111.95,136.24) | 6.42 (5.80,7.10) |  | 206.27 (156.97,270.06) | 5.18 (3.97,6.74) |  | 1.70 (1.12,2.28) | -0.66 (-1.17,-0.16) |
| Tuvalu | 0.70 (0.56,0.89) | 11.52 (9.37,14.49) |  | 1.05 (0.84,1.33) | 10.88 (8.76,13.79) |  | 1.23 (1.18,1.27) | -0.21 (-0.23,-0.19) |
| Ukraine | 14611.72 (13369.16,15827.68) | 20.41 (18.64,22.12) |  | 12319.14 (9192.74,15854.89) | 15.67 (11.64,20.17) |  | -0.78 (-0.93,-0.64) | -1.06 (-1.20,-0.91) |
| Union of the Comoros | 16.85 (11.63,22.34) | 9.19 (6.60,12.07) |  | 44.85 (32.04,60.62) | 10.04 (7.17,13.40) |  | 3.15 (3.00,3.31) | 0.24 (0.13,0.34) |
| United Arab Emirates | 75.29 (50.44,102.70) | 17.62 (12.20,23.74) |  | 326.47 (235.69,488.71) | 13.05 (9.99,18.53) |  | 5.54 (5.28,5.80) | 0.79 (0.22,1.36) |
| United Kingdom of Great Britain and Northern Ireland | 22692.27 (21466.93,23344.36) | 24.40 (23.14,25.09) |  | 22130.21 (19879.61,23362.10) | 15.72 (14.30,16.51) |  | -0.06 (-0.23,0.11) | -1.45 (-1.56,-1.34) |
| United Mexican States | 2497.56 (2407.12,2587.93) | 6.42 (6.15,6.66) |  | 10930.08 (9651.06,12242.21) | 8.78 (7.77,9.81) |  | 5.06 (4.91,5.21) | 1.13 (0.98,1.28) |
| United Republic of Tanzania | 878.09 (710.22,1080.84) | 8.74 (7.19,10.52) |  | 2231.71 (1652.53,2905.10) | 9.50 (7.18,12.14) |  | 3.04 (2.96,3.12) | 0.28 (0.23,0.33) |
| United States of America | 67231.68 (61658.00,70474.93) | 20.57 (18.92,21.54) |  | 75087.06 (68061.57,79710.66) | 12.79 (11.72,13.51) |  | 0.18 (0.10,0.26) | -1.71 (-1.79,-1.63) |
| United States Virgin Islands | 16.04 (13.64,18.95) | 21.41 (18.15,25.33) |  | 24.44 (18.71,31.31) | 13.95 (10.72,17.67) |  | 1.50 (1.16,1.83) | -1.23 (-1.47,-0.99) |

Abbreviations: ASMR, age-standardized deaths rate; EAPC, estimated annual percentage change; SDI, Sociodemographic Index; UI, uncertainty interval.

|  |  |
| --- | --- |

**eTable6.** DALYs of CRC Between 1990 and 2021 at the 204 Countries and Territories Level

| Location | Rate per 100,000 (95% UI) | | | | | | | |
| --- | --- | --- | --- | --- | --- | --- | --- | --- |
|  | 1990 |  |  | 2021 |  |  | 1990-2021 |  |
|  | DALYs cases | ASDR |  | DALYs cases | ASDR |  | Cases change | EAPC (95% CI) |
| Countries | | | | | | | | |
| American Samoa | 88.17 (73.57,104.62) | 355.65 (302.58,416.80) |  | 179.74 (149.41,222.88) | 366.75 (306.83,450.38) |  | 2.30 (2.21,2.39) | 0.22 (0.10,0.34) |
| Antigua and Barbuda | 145.95 (133.45,159.21) | 274.16 (250.52,300.07) |  | 342.58 (314.98,370.48) | 320.56 (294.20,346.47) |  | 3.00 (2.83,3.17) | 0.61 (0.45,0.77) |
| Arab Republic of Egypt | 46641.02 (41635.72,52035.82) | 146.25 (130.00,164.50) |  | 149499.57 (121974.88,183001.04) | 212.77 (174.34,258.80) |  | 4.33 (4.13,4.53) | 1.80 (1.55,2.05) |
| Argentine Republic | 156058.42 (141297.17,171552.79) | 483.66 (437.93,531.63) |  | 243772.90 (213624.64,274727.89) | 443.27 (387.89,500.57) |  | 1.73 (1.53,1.94) | 0.00 (-0.20,0.21) |
| Australia | 106528.92 (97307.09,116426.58) | 552.27 (505.74,602.89) |  | 134817.46 (118206.53,153417.80) | 315.46 (278.06,356.65) |  | 0.49 (0.38,0.60) | -2.06 (-2.17,-1.95) |
| Barbados | 1190.09 (1084.20,1305.00) | 422.19 (384.78,461.92) |  | 2505.48 (1949.11,3102.76) | 496.33 (387.48,616.82) |  | 2.83 (2.67,2.99) | 0.86 (0.62,1.09) |
| Belize | 142.87 (130.69,157.33) | 147.75 (135.15,163.02) |  | 696.43 (597.34,811.17) | 218.78 (188.42,255.02) |  | 5.42 (5.04,5.79) | 1.30 (0.83,1.78) |
| Bermuda | 386.04 (352.73,423.75) | 616.72 (563.57,676.94) |  | 523.00 (436.57,642.96) | 396.54 (329.53,490.21) |  | 1.26 (1.04,1.48) | -1.26 (-1.44,-1.08) |
| Bolivarian Republic of Venezuela | 22364.49 (20757.46,24071.25) | 211.56 (195.10,228.30) |  | 77061.05 (56518.16,100615.77) | 254.94 (187.83,332.69) |  | 4.27 (4.12,4.42) | 0.58 (0.47,0.70) |
| Bosnia and Herzegovina | 14004.70 (12177.26,15936.57) | 329.20 (287.30,372.90) |  | 26033.61 (19965.64,32600.88) | 423.43 (323.22,528.91) |  | 2.38 (2.06,2.70) | 1.17 (0.94,1.40) |
| Brunei Darussalam | 797.46 (638.18,966.12) | 674.44 (540.56,813.05) |  | 1794.39 (1514.11,2112.22) | 466.43 (393.67,547.69) |  | 3.09 (2.71,3.47) | -0.72 (-1.03,-0.41) |
| Burkina Faso | 5200.72 (4141.61,6533.70) | 116.95 (94.09,146.10) |  | 13033.35 (9456.95,16998.12) | 134.58 (99.55,173.68) |  | 3.29 (3.12,3.46) | 0.66 (0.57,0.76) |
| Canada | 150885.62 (139350.97,162674.34) | 469.27 (433.30,506.53) |  | 215088.13 (191662.39,238146.12) | 317.47 (285.08,351.22) |  | 1.41 (1.29,1.52) | -1.09 (-1.18,-1.00) |
| Central African Republic | 3113.50 (2023.89,4222.84) | 242.27 (164.09,327.82) |  | 6016.98 (3932.95,8839.67) | 229.71 (154.29,327.17) |  | 2.21 (2.15,2.26) | -0.17 (-0.24,-0.10) |
| Commonwealth of Dominica | 165.73 (149.55,188.10) | 280.60 (253.75,315.84) |  | 275.68 (230.62,335.24) | 331.61 (278.95,402.29) |  | 1.83 (1.72,1.94) | 0.67 (0.60,0.74) |
| Commonwealth of the Bahamas | 668.34 (600.03,744.70) | 399.03 (358.66,442.94) |  | 1863.70 (1487.91,2332.59) | 444.71 (357.74,553.53) |  | 3.69 (3.56,3.82) | 0.65 (0.54,0.76) |
| Cook Islands | 23.04 (18.58,27.35) | 178.86 (146.12,209.07) |  | 32.40 (25.77,41.10) | 129.05 (102.56,164.07) |  | 1.01 (0.84,1.18) | -1.17 (-1.34,-0.99) |
| Czech Republic | 108051.71 (96936.21,120857.36) | 788.42 (708.34,879.45) |  | 92436.22 (77340.63,109800.40) | 446.39 (371.66,531.64) |  | -0.71 (-0.85,-0.57) | -2.15 (-2.33,-1.96) |
| Democratic People's Republic of Korea | 53752.74 (38267.79,75627.05) | 303.97 (220.29,419.72) |  | 95104.46 (63452.77,143702.10) | 283.00 (189.17,427.86) |  | 1.96 (1.85,2.06) | -0.10 (-0.18,-0.02) |
| Democratic Republic of Sao Tome and Principe | 116.59 (96.29,139.98) | 179.60 (149.71,213.36) |  | 263.68 (208.00,337.49) | 231.19 (188.42,286.26) |  | 2.74 (2.60,2.88) | 0.94 (0.86,1.01) |
| Democratic Republic of the Congo | 26806.91 (20791.17,34700.23) | 158.32 (123.82,204.66) |  | 63847.37 (43731.99,93516.27) | 156.42 (107.84,227.19) |  | 2.89 (2.60,3.18) | 0.04 (-0.15,0.24) |
| Democratic Republic of Timor-Leste | 721.50 (501.77,980.98) | 212.77 (151.98,282.77) |  | 2044.44 (1534.11,2686.97) | 227.22 (171.70,296.93) |  | 3.73 (3.46,3.99) | 0.32 (0.10,0.54) |
| Democratic Socialist Republic of Sri Lanka | 13791.75 (12103.26,15633.89) | 118.27 (103.46,133.84) |  | 29736.57 (19284.02,41877.83) | 109.54 (71.59,153.93) |  | 2.84 (2.65,3.02) | 0.07 (-0.11,0.24) |
| Dominican Republic | 6828.04 (5709.29,8014.24) | 173.95 (145.45,204.25) |  | 22066.63 (17243.17,28379.90) | 215.86 (168.90,277.61) |  | 4.25 (4.07,4.44) | 1.15 (1.00,1.30) |
| Eastern Republic of Uruguay | 22257.58 (20432.69,24129.30) | 583.05 (533.50,631.50) |  | 31771.54 (28208.15,35690.03) | 598.78 (533.29,672.27) |  | 1.03 (0.93,1.12) | -0.01 (-0.11,0.09) |
| Federal Democratic Republic of Ethiopia | 113209.06 (61301.49,140527.86) | 528.87 (298.28,650.33) |  | 158082.67 (130517.21,190982.30) | 343.04 (284.97,415.16) |  | 0.63 (0.31,0.96) | -1.74 (-1.95,-1.53) |
| Federal Democratic Republic of Nepal | 11515.43 (7870.82,15997.72) | 107.60 (75.33,147.97) |  | 25511.78 (18732.24,33825.12) | 103.55 (76.75,136.66) |  | 2.81 (2.44,3.19) | -0.03 (-0.39,0.32) |
| Federal Republic of Germany | 711506.65 (649316.06,773808.27) | 569.63 (520.88,618.19) |  | 586274.79 (514071.93,658727.78) | 323.01 (286.33,360.79) |  | -0.83 (-0.92,-0.73) | -2.07 (-2.19,-1.96) |
| Federal Republic of Nigeria | 55084.62 (42152.81,71187.51) | 119.91 (92.85,153.40) |  | 122930.39 (91479.87,158965.30) | 126.42 (98.66,158.92) |  | 2.71 (2.59,2.83) | 0.31 (0.23,0.38) |
| Federal Republic of Somalia | 7123.71 (4589.62,10326.23) | 248.01 (161.08,356.98) |  | 17562.93 (11257.27,27028.51) | 243.25 (156.39,363.26) |  | 3.08 (3.00,3.15) | 0.04 (-0.02,0.09) |
| Federated States of Micronesia | 164.98 (125.62,213.77) | 316.65 (245.83,406.90) |  | 243.96 (172.32,337.29) | 303.82 (219.46,415.67) |  | 1.28 (1.17,1.40) | -0.14 (-0.18,-0.10) |
| Federative Republic of Brazil | 215010.96 (205934.94,225207.46) | 226.33 (215.25,237.51) |  | 730348.39 (681571.03,772153.39) | 287.32 (267.76,303.88) |  | 4.04 (3.92,4.16) | 0.75 (0.65,0.85) |
| French Republic | 410040.41 (379655.53,441528.75) | 506.41 (470.14,544.85) |  | 437361.41 (375392.17,495051.46) | 324.49 (282.40,367.06) |  | 0.32 (0.27,0.36) | -1.37 (-1.43,-1.31) |
| Gabonese Republic | 1958.69 (1171.35,2783.44) | 331.64 (199.60,467.76) |  | 3614.78 (2570.46,4833.16) | 319.35 (230.62,421.91) |  | 1.98 (1.88,2.08) | -0.14 (-0.20,-0.08) |
| Georgia | 17503.91 (15442.84,19827.01) | 278.64 (246.19,315.15) |  | 18598.27 (15912.67,21533.41) | 328.78 (281.26,381.66) |  | 1.13 (0.70,1.57) | 1.57 (1.15,1.99) |
| Grand Duchy of Luxembourg | 3105.84 (2896.82,3302.32) | 581.81 (542.30,619.96) |  | 3185.39 (2810.24,3545.88) | 299.97 (264.08,334.57) |  | 0.11 (0.03,0.20) | -2.09 (-2.27,-1.90) |
| Greenland | 374.16 (325.39,437.60) | 1003.05 (881.56,1163.01) |  | 409.53 (344.58,494.69) | 572.67 (484.22,684.00) |  | 0.37 (0.32,0.41) | -1.83 (-1.90,-1.75) |
| Grenada | 223.02 (193.49,257.15) | 321.69 (279.13,371.19) |  | 443.22 (369.44,519.16) | 387.52 (324.20,453.25) |  | 2.65 (2.52,2.77) | 0.83 (0.68,0.98) |
| Guam | 293.10 (255.43,332.80) | 356.48 (307.61,403.09) |  | 594.26 (516.79,679.33) | 291.86 (253.74,332.83) |  | 2.73 (2.51,2.95) | -0.12 (-0.44,0.20) |
| Hashemite Kingdom of Jordan | 4267.90 (3447.36,5152.45) | 273.88 (222.82,328.14) |  | 17097.31 (13056.23,23271.80) | 205.95 (158.78,277.99) |  | 4.55 (4.39,4.70) | -0.91 (-1.13,-0.68) |
| Hellenic Republic | 48871.53 (44858.90,52811.12) | 326.07 (299.85,351.93) |  | 68382.37 (60458.73,75648.53) | 297.27 (268.42,326.68) |  | 0.87 (0.70,1.03) | -0.55 (-0.71,-0.39) |
| Hungary | 101438.64 (85820.90,116735.49) | 700.15 (593.01,804.26) |  | 113138.87 (95756.57,135404.58) | 614.96 (519.37,736.20) |  | 0.33 (0.11,0.56) | -0.53 (-0.76,-0.30) |
| Independent State of Papua New Guinea | 2216.15 (1530.93,3028.82) | 101.97 (73.33,137.01) |  | 5699.86 (4328.90,7238.01) | 91.65 (70.55,115.21) |  | 3.07 (2.88,3.26) | -0.44 (-0.53,-0.35) |
| Independent State of Samoa | 194.59 (157.71,235.20) | 222.16 (180.37,265.86) |  | 331.57 (260.81,414.58) | 221.44 (176.93,274.29) |  | 1.73 (1.66,1.81) | -0.02 (-0.10,0.06) |
| Ireland | 22583.18 (20956.34,24171.33) | 565.04 (523.25,605.38) |  | 23968.28 (20725.68,27202.26) | 308.17 (267.90,349.96) |  | 0.36 (0.23,0.48) | -1.84 (-1.92,-1.75) |
| Islamic Republic of Afghanistan | 23297.29 (8037.45,36521.49) | 320.35 (115.69,502.95) |  | 40776.89 (17118.61,64991.37) | 324.71 (146.82,503.12) |  | 1.75 (1.50,2.00) | 0.16 (0.09,0.22) |
| Islamic Republic of Iran | 50997.80 (42389.08,58906.08) | 177.13 (148.77,203.86) |  | 142537.03 (122423.24,158787.71) | 174.30 (149.14,193.93) |  | 3.95 (3.70,4.21) | 0.42 (0.19,0.64) |
| Islamic Republic of Mauritania | 1515.45 (1162.75,1870.37) | 147.90 (114.18,181.79) |  | 3352.51 (2440.98,4579.22) | 152.11 (111.06,206.82) |  | 2.67 (2.43,2.91) | 0.11 (-0.06,0.28) |
| Islamic Republic of Pakistan | 74920.02 (63620.16,85703.82) | 124.61 (105.38,142.84) |  | 212736.13 (169934.15,266858.29) | 154.08 (125.46,192.31) |  | 3.13 (2.97,3.29) | 0.40 (0.15,0.64) |
| Jamaica | 4276.95 (3793.69,4744.30) | 240.18 (213.25,266.24) |  | 10907.72 (8261.79,14549.92) | 353.13 (266.97,471.23) |  | 3.07 (2.67,3.47) | 1.27 (0.87,1.67) |
| Japan | 752196.86 (717565.39,777195.40) | 445.57 (424.43,460.65) |  | 1171590.98 (1020788.47,1259680.66) | 358.44 (327.93,378.99) |  | 1.37 (1.29,1.46) | -0.77 (-0.84,-0.71) |
| Kingdom of Bahrain | 556.21 (489.30,645.85) | 282.95 (249.02,326.38) |  | 2212.30 (1741.81,3080.58) | 227.10 (183.24,307.76) |  | 4.51 (4.41,4.62) | -1.16 (-1.43,-0.90) |
| Kingdom of Belgium | 79632.70 (71436.30,86776.49) | 527.79 (474.83,576.02) |  | 67951.87 (59199.97,76500.28) | 301.55 (264.48,338.67) |  | -0.42 (-0.52,-0.31) | -1.76 (-1.88,-1.65) |
| Kingdom of Bhutan | 336.93 (210.09,466.68) | 117.59 (73.37,162.10) |  | 675.85 (494.09,887.64) | 106.36 (78.43,138.93) |  | 2.22 (2.11,2.33) | -0.40 (-0.51,-0.29) |
| Kingdom of Cambodia | 17059.60 (10911.62,23005.55) | 333.46 (216.76,443.32) |  | 47236.22 (34945.74,61843.64) | 353.09 (262.55,456.87) |  | 3.41 (3.31,3.51) | 0.15 (0.09,0.22) |
| Kingdom of Denmark | 41173.88 (37232.55,45122.69) | 530.70 (481.49,581.60) |  | 45208.63 (39100.09,50736.56) | 384.64 (333.80,432.46) |  | -0.03 (-0.24,0.19) | -1.36 (-1.63,-1.08) |
| Kingdom of Eswatini | 862.49 (609.23,1117.89) | 277.16 (193.02,357.85) |  | 2402.33 (1520.80,3340.98) | 378.16 (242.58,517.06) |  | 3.61 (2.90,4.32) | 1.41 (0.83,1.99) |
| Kingdom of Lesotho | 1360.39 (1021.73,1786.05) | 157.89 (119.78,205.22) |  | 3789.64 (2592.09,5497.32) | 328.58 (228.45,473.52) |  | 4.05 (3.45,4.65) | 3.20 (2.70,3.69) |
| Kingdom of Morocco | 22942.60 (17754.61,28906.17) | 149.62 (116.57,187.19) |  | 71875.61 (52058.08,95041.52) | 200.91 (146.47,263.30) |  | 3.98 (3.86,4.11) | 1.16 (1.06,1.26) |
| Kingdom of Norway | 34432.93 (32283.34,36014.88) | 530.51 (501.41,554.62) |  | 36613.71 (32948.98,39351.76) | 369.37 (337.20,395.53) |  | 0.13 (0.03,0.22) | -1.22 (-1.31,-1.12) |
| Kingdom of Saudi Arabia | 10092.43 (7178.71,13422.66) | 143.54 (103.96,189.79) |  | 50305.71 (38171.67,65034.56) | 190.23 (152.15,235.94) |  | 5.47 (5.27,5.68) | 1.18 (0.84,1.53) |
| Kingdom of Spain | 234647.69 (215630.38,253273.55) | 444.21 (408.14,480.03) |  | 340723.22 (294865.82,383569.17) | 361.91 (315.71,403.81) |  | 1.36 (1.18,1.55) | -0.59 (-0.73,-0.46) |
| Kingdom of Sweden | 59148.43 (53472.01,64669.45) | 412.60 (375.63,449.61) |  | 61587.48 (51468.83,71399.81) | 293.06 (249.23,339.71) |  | 0.27 (0.14,0.40) | -0.91 (-1.08,-0.75) |
| Kingdom of Thailand | 124081.96 (104757.86,144741.41) | 320.18 (271.58,372.78) |  | 397084.48 (298452.28,509792.46) | 380.26 (287.87,486.34) |  | 3.53 (3.37,3.70) | 0.26 (0.12,0.41) |
| Kingdom of the Netherlands | 104933.40 (97912.02,113230.23) | 533.63 (498.50,574.81) |  | 151494.45 (135284.01,167033.75) | 444.07 (399.16,488.14) |  | 1.32 (1.16,1.49) | -0.51 (-0.69,-0.32) |
| Kingdom of Tonga | 92.53 (75.59,110.87) | 162.09 (132.24,193.54) |  | 132.88 (102.64,171.30) | 163.21 (125.84,209.31) |  | 1.08 (0.94,1.21) | -0.02 (-0.12,0.09) |
| Kyrgyz Republic | 9322.67 (8346.20,10302.42) | 296.13 (265.87,326.32) |  | 9606.52 (7879.25,11525.44) | 181.61 (148.95,218.19) |  | 0.13 (-0.16,0.42) | -1.42 (-1.58,-1.26) |
| Lao People's Democratic Republic | 8170.37 (4269.17,11409.98) | 353.61 (189.35,489.69) |  | 16726.77 (11671.71,22481.59) | 323.48 (229.50,429.29) |  | 2.22 (2.15,2.28) | -0.39 (-0.43,-0.34) |
| Lebanese Republic | 6856.66 (4552.31,8698.82) | 309.78 (208.42,389.03) |  | 14290.98 (11659.32,18127.42) | 236.40 (193.55,298.75) |  | 2.97 (2.73,3.22) | -0.43 (-0.65,-0.21) |
| Malaysia | 39650.96 (34883.74,45172.89) | 399.40 (351.23,454.40) |  | 122302.94 (106768.65,136769.39) | 418.71 (364.59,469.07) |  | 3.62 (3.44,3.80) | -0.01 (-0.18,0.15) |
| Mongolia | 2379.58 (1860.72,3033.46) | 206.20 (159.68,262.13) |  | 5401.18 (4202.11,6649.92) | 207.23 (162.14,256.08) |  | 2.65 (2.41,2.89) | -0.15 (-0.29,-0.00) |
| Montenegro | 2102.84 (1802.39,2474.57) | 331.36 (284.55,388.34) |  | 3665.36 (3024.19,4476.96) | 378.80 (314.03,462.92) |  | 1.97 (1.81,2.13) | 0.56 (0.45,0.67) |
| New Zealand | 27094.49 (24747.24,29769.60) | 706.98 (646.50,775.40) |  | 31922.64 (27860.61,36063.48) | 390.26 (342.89,440.26) |  | 0.41 (0.29,0.52) | -2.05 (-2.15,-1.95) |
| North Macedonia | 7155.82 (6203.03,8121.58) | 375.64 (326.42,427.47) |  | 13343.91 (10509.49,16397.47) | 404.98 (321.47,494.20) |  | 2.16 (1.85,2.46) | 0.28 (-0.03,0.59) |
| Northern Mariana Islands | 80.75 (61.04,108.84) | 365.90 (290.65,465.11) |  | 194.23 (163.92,216.93) | 358.92 (305.62,398.44) |  | 3.13 (2.97,3.28) | -0.02 (-0.26,0.23) |
| Palestine | 3889.51 (2873.65,5084.35) | 425.17 (317.32,550.85) |  | 9435.67 (7748.42,11315.19) | 342.54 (282.99,404.33) |  | 2.99 (2.90,3.07) | -0.65 (-0.78,-0.52) |
| People's Democratic Republic of Algeria | 13828.91 (11360.60,16482.56) | 113.64 (95.15,133.63) |  | 35662.14 (27589.56,45698.77) | 99.94 (78.40,126.74) |  | 3.17 (3.06,3.28) | -0.25 (-0.36,-0.13) |
| People's Republic of Bangladesh | 63532.58 (48464.80,83225.90) | 120.99 (92.77,158.33) |  | 128048.17 (92939.32,180918.72) | 88.88 (65.03,124.89) |  | 2.51 (2.33,2.70) | -0.99 (-1.09,-0.89) |
| People's Republic of China | 3565195.70 (3027610.19,4106701.24) | 390.63 (333.24,448.92) |  | 6848389.89 (5513406.57,8284228.27) | 331.73 (267.78,400.70) |  | 2.08 (1.97,2.19) | -0.62 (-0.71,-0.54) |
| Plurinational State of Bolivia | 10521.88 (6675.19,14194.52) | 311.72 (199.12,420.12) |  | 28732.23 (19714.74,40354.55) | 308.93 (211.81,431.97) |  | 3.41 (3.37,3.45) | -0.04 (-0.08,-0.00) |
| Portuguese Republic | 67278.38 (60120.21,76002.16) | 499.42 (446.16,562.70) |  | 87869.11 (75968.93,100292.59) | 383.54 (331.36,439.81) |  | 1.00 (0.76,1.24) | -0.77 (-0.99,-0.55) |
| Principality of Andorra | 315.76 (228.32,447.97) | 545.05 (396.14,771.54) |  | 528.45 (365.06,711.95) | 344.77 (238.41,466.30) |  | 1.61 (1.38,1.84) | -1.16 (-1.40,-0.92) |
| Principality of Monaco | 348.87 (271.36,422.42) | 533.71 (419.96,648.54) |  | 500.64 (400.59,603.17) | 551.88 (440.65,676.50) |  | 1.24 (1.14,1.35) | 0.22 (0.11,0.33) |
| Puerto Rico | 12025.55 (10970.52,13110.89) | 335.03 (304.78,365.77) |  | 21159.11 (17665.07,25426.46) | 337.60 (281.28,406.32) |  | 1.84 (1.54,2.13) | -0.00 (-0.18,0.18) |
| Republic of Albania | 3524.65 (2977.64,4212.05) | 167.95 (142.30,200.53) |  | 6699.63 (5148.22,8974.58) | 156.46 (120.27,209.47) |  | 2.46 (2.26,2.65) | 0.11 (-0.10,0.32) |
| Republic of Angola | 8595.95 (6156.88,11232.58) | 195.62 (143.43,253.90) |  | 27221.12 (19416.60,36786.31) | 203.35 (148.46,271.46) |  | 3.94 (3.72,4.17) | 0.17 (0.05,0.28) |
| Republic of Armenia | 10480.46 (9651.68,11265.32) | 360.75 (331.27,389.01) |  | 12935.78 (11310.89,14863.50) | 301.97 (265.01,345.93) |  | 0.86 (0.70,1.03) | -0.32 (-0.52,-0.11) |
| Republic of Austria | 62034.40 (57167.62,66732.22) | 540.11 (496.64,582.50) |  | 44313.38 (38999.07,50543.15) | 253.07 (223.60,287.33) |  | -1.02 (-1.08,-0.97) | -2.46 (-2.52,-2.40) |
| Republic of Azerbaijan | 13611.65 (11494.27,15763.38) | 247.59 (208.97,286.22) |  | 20515.90 (15353.01,26254.12) | 184.50 (138.24,234.39) |  | 1.47 (1.09,1.84) | -0.73 (-1.00,-0.45) |
| Republic of Belarus | 52966.38 (46053.78,60454.50) | 409.49 (356.29,467.39) |  | 65916.96 (49884.06,84086.79) | 415.90 (314.40,530.09) |  | -0.10 (-0.45,0.24) | -0.69 (-1.00,-0.37) |
| Republic of Benin | 2359.45 (1981.63,2803.98) | 114.26 (96.35,135.37) |  | 6748.52 (4927.15,9041.96) | 123.35 (93.20,162.85) |  | 3.62 (3.47,3.78) | 0.41 (0.31,0.51) |
| Republic of Botswana | 1370.23 (991.95,1802.24) | 231.19 (168.48,298.88) |  | 3548.05 (2535.03,5177.08) | 226.73 (167.80,313.81) |  | 3.01 (2.79,3.23) | -0.03 (-0.27,0.20) |
| Republic of Bulgaria | 63685.78 (56294.81,72184.83) | 520.65 (461.61,589.83) |  | 81397.51 (66580.59,97733.05) | 605.00 (493.22,726.67) |  | 1.20 (1.04,1.37) | 0.85 (0.68,1.02) |
| Republic of Burundi | 6003.09 (4481.73,7778.29) | 239.24 (180.34,308.34) |  | 10583.67 (7424.77,15179.20) | 193.62 (138.14,275.06) |  | 1.62 (1.23,2.01) | -1.01 (-1.18,-0.84) |
| Republic of Cabo Verde | 178.63 (151.46,208.33) | 78.44 (66.53,92.01) |  | 795.47 (623.00,956.26) | 179.56 (140.03,216.00) |  | 4.50 (4.12,4.89) | 2.14 (1.73,2.55) |
| Republic of Cameroon | 7023.22 (5866.12,8400.98) | 150.03 (126.62,177.31) |  | 22776.37 (15314.50,31636.22) | 167.20 (116.22,226.65) |  | 4.02 (3.95,4.10) | 0.48 (0.39,0.56) |
| Republic of Chad | 2860.25 (2326.77,3568.82) | 98.83 (80.13,123.68) |  | 8896.17 (6554.40,11676.05) | 142.86 (106.23,186.72) |  | 3.96 (3.90,4.03) | 1.41 (1.29,1.54) |
| Republic of Chile | 27911.59 (25487.07,30376.40) | 273.96 (250.16,298.57) |  | 74017.40 (65141.85,83182.88) | 292.24 (258.58,329.15) |  | 3.58 (3.43,3.74) | 0.53 (0.40,0.67) |
| Republic of Colombia | 41678.20 (38476.13,44735.19) | 219.06 (201.46,235.91) |  | 142012.43 (116973.76,171036.64) | 258.01 (212.77,310.81) |  | 3.91 (3.79,4.03) | 0.38 (0.22,0.54) |
| Republic of Costa Rica | 3959.15 (3578.53,4355.88) | 216.69 (196.16,238.03) |  | 20357.90 (17641.11,23650.83) | 370.71 (321.50,430.24) |  | 5.69 (5.47,5.91) | 1.99 (1.76,2.22) |
| Republic of Croatia | 4198.15 (3300.77,5141.72) | 94.64 (75.99,113.37) |  | 11490.08 (8141.64,16459.47) | 92.14 (67.87,128.91) |  | 3.13 (2.97,3.29) | -0.13 (-0.24,-0.03) |
| Republic of Cuba | 31643.48 (27892.26,35468.89) | 527.48 (467.04,588.25) |  | 45573.05 (39330.55,52194.24) | 524.86 (450.88,601.57) |  | 1.26 (1.02,1.50) | 0.07 (-0.13,0.28) |
| Republic of Cyprus | 36605.83 (33656.05,39714.23) | 355.66 (326.68,386.26) |  | 74400.11 (62856.62,86954.74) | 388.50 (327.70,454.12) |  | 2.50 (2.38,2.61) | 0.41 (0.28,0.54) |
| Republic of C么te d'Ivoire | 2765.47 (2354.11,3279.24) | 372.42 (316.80,440.07) |  | 4982.63 (4174.77,6015.95) | 247.79 (208.91,294.36) |  | 2.27 (2.14,2.40) | -1.00 (-1.13,-0.87) |
| Republic of Djibouti | 365.26 (244.83,516.21) | 230.22 (159.38,323.52) |  | 2070.56 (1426.36,2994.36) | 290.23 (205.38,408.80) |  | 5.78 (5.65,5.92) | 0.77 (0.67,0.86) |
| Republic of Ecuador | 8809.36 (8033.44,9561.64) | 158.96 (145.01,172.47) |  | 35828.83 (27566.31,45730.83) | 215.97 (166.95,275.35) |  | 4.92 (4.54,5.30) | 1.26 (0.94,1.59) |
| Republic of El Salvador | 4149.36 (3799.24,4540.00) | 129.34 (118.16,142.04) |  | 12737.53 (10187.14,15570.88) | 206.97 (165.35,253.14) |  | 3.74 (3.55,3.94) | 1.56 (1.39,1.74) |
| Republic of Equatorial Guinea | 452.84 (314.32,598.58) | 214.28 (152.08,278.98) |  | 1448.62 (948.44,2102.10) | 245.07 (165.22,345.96) |  | 4.12 (3.79,4.45) | 0.60 (0.44,0.77) |
| Republic of Estonia | 9082.88 (8305.36,9959.27) | 445.38 (406.75,488.00) |  | 10062.36 (8192.51,11781.90) | 382.11 (310.79,447.00) |  | 0.05 (-0.15,0.25) | -0.86 (-1.04,-0.68) |
| Republic of Fiji | 897.59 (716.09,1073.90) | 219.15 (175.55,263.36) |  | 1814.62 (1321.73,2381.43) | 226.96 (167.30,295.51) |  | 2.57 (2.33,2.81) | 0.18 (-0.05,0.40) |
| Republic of Finland | 23112.55 (21410.24,24866.32) | 329.49 (304.70,355.06) |  | 29540.00 (25812.32,33215.37) | 243.95 (215.21,273.85) |  | 0.83 (0.71,0.95) | -0.98 (-1.07,-0.89) |
| Republic of Ghana | 8070.65 (6311.81,10229.66) | 120.06 (94.69,151.08) |  | 26615.39 (20382.55,34054.57) | 150.82 (117.21,189.48) |  | 4.13 (4.06,4.20) | 0.96 (0.89,1.04) |
| Republic of Guatemala | 4318.03 (4004.24,4667.30) | 111.83 (103.26,121.41) |  | 19707.04 (16781.41,23068.29) | 168.37 (143.72,196.84) |  | 5.10 (4.78,5.42) | 1.23 (0.93,1.54) |
| Republic of Guinea | 3313.14 (2657.52,4040.29) | 97.02 (77.96,118.31) |  | 6689.59 (4788.94,9195.94) | 110.34 (80.27,151.15) |  | 2.19 (2.04,2.35) | 0.55 (0.45,0.64) |
| Republic of Guinea-Bissau | 780.81 (490.15,1003.81) | 181.17 (115.38,231.91) |  | 1609.31 (1174.45,2092.57) | 195.76 (146.32,248.83) |  | 2.58 (2.44,2.71) | 0.45 (0.37,0.52) |
| Republic of Guyana | 1105.04 (966.72,1245.70) | 272.79 (239.67,306.88) |  | 2203.26 (1653.10,2866.73) | 325.81 (246.26,422.11) |  | 2.70 (2.46,2.95) | 0.88 (0.62,1.15) |
| Republic of Haiti | 12343.50 (7639.12,16395.00) | 358.34 (231.51,472.76) |  | 25546.67 (17158.59,36499.65) | 325.88 (222.11,464.01) |  | 2.58 (2.49,2.67) | -0.12 (-0.19,-0.05) |
| Republic of Honduras | 2502.82 (2119.45,2948.27) | 107.03 (90.85,126.01) |  | 9933.30 (7614.43,13111.44) | 147.97 (116.10,194.46) |  | 4.73 (4.60,4.85) | 1.17 (1.03,1.32) |
| Republic of Iceland | 990.79 (911.28,1077.37) | 352.63 (323.43,383.55) |  | 1363.36 (1173.86,1576.52) | 239.60 (207.01,275.80) |  | 1.12 (1.02,1.22) | -1.20 (-1.30,-1.09) |
| Republic of India | 636945.68 (548000.80,719399.08) | 118.08 (101.11,133.98) |  | 1541696.22 (1369023.49,1755414.62) | 120.93 (107.62,137.15) |  | 2.81 (2.68,2.95) | -0.05 (-0.18,0.09) |
| Republic of Indonesia | 269726.19 (205455.74,328285.38) | 239.09 (185.80,290.41) |  | 767833.22 (572468.07,973190.12) | 297.58 (223.31,371.79) |  | 3.37 (3.32,3.42) | 0.72 (0.63,0.82) |
| Republic of Iraq | 12499.96 (9984.00,15205.63) | 141.53 (113.71,173.65) |  | 41145.00 (30690.48,53217.65) | 154.27 (116.39,193.92) |  | 4.12 (3.96,4.29) | 0.35 (0.19,0.51) |
| Republic of Italy | 382791.23 (362379.55,399473.34) | 442.97 (419.62,461.81) |  | 427742.23 (381470.13,461600.90) | 309.66 (284.37,331.08) |  | 0.35 (0.21,0.49) | -1.22 (-1.36,-1.07) |
| Republic of Kazakhstan | 55058.41 (49415.70,60949.23) | 410.39 (366.42,455.55) |  | 49100.47 (42011.60,56786.02) | 261.66 (224.14,303.16) |  | -0.16 (-0.29,-0.04) | -1.20 (-1.40,-0.99) |
| Republic of Kenya | 10989.70 (8872.49,13252.78) | 119.33 (96.43,144.51) |  | 43750.07 (35787.75,55865.55) | 171.46 (141.02,215.33) |  | 4.97 (4.76,5.17) | 1.60 (1.38,1.83) |
| Republic of Kiribati | 94.25 (77.50,117.69) | 226.77 (184.48,283.66) |  | 181.78 (134.62,246.33) | 223.53 (170.29,299.18) |  | 2.13 (2.11,2.15) | -0.17 (-0.25,-0.10) |
| Republic of Korea | 109036.24 (94901.80,124319.55) | 336.07 (291.31,382.59) |  | 244907.01 (204210.06,286793.18) | 267.43 (223.39,313.79) |  | 2.61 (2.45,2.76) | -0.87 (-1.05,-0.69) |
| Republic of Latvia | 15345.57 (13607.14,17349.96) | 432.47 (382.89,489.48) |  | 14214.48 (11640.42,16997.64) | 380.02 (310.97,453.77) |  | -0.20 (-0.37,-0.03) | -0.43 (-0.59,-0.27) |
| Republic of Liberia | 1366.24 (1084.37,1718.08) | 115.24 (92.35,143.37) |  | 2921.84 (1766.00,4515.18) | 124.19 (77.79,186.56) |  | 2.90 (2.52,3.27) | 0.55 (0.33,0.78) |
| Republic of Lithuania | 17877.23 (16429.86,19409.06) | 399.38 (365.64,434.46) |  | 20787.19 (17218.01,24514.52) | 380.34 (315.77,449.63) |  | 0.45 (0.25,0.65) | -0.20 (-0.37,-0.02) |
| Republic of Madagascar | 11316.20 (8959.64,13604.10) | 202.99 (163.12,244.41) |  | 25296.80 (18063.89,33192.96) | 193.53 (139.73,252.26) |  | 2.81 (2.53,3.08) | -0.06 (-0.17,0.06) |
| Republic of Malawi | 4177.57 (3447.84,5177.43) | 98.85 (82.07,121.55) |  | 9128.45 (7003.29,12937.39) | 109.53 (85.08,150.62) |  | 2.33 (2.21,2.45) | 0.17 (-0.03,0.36) |
| Republic of Maldives | 183.91 (97.45,236.96) | 182.21 (107.23,230.28) |  | 356.82 (288.62,450.03) | 94.22 (76.20,117.52) |  | 1.77 (1.53,2.01) | -2.49 (-2.63,-2.35) |
| Republic of Mali | 7028.47 (5922.42,8309.34) | 166.41 (141.80,195.77) |  | 15642.12 (11830.56,20127.56) | 164.24 (125.81,208.77) |  | 2.99 (2.75,3.23) | 0.16 (0.05,0.27) |
| Republic of Malta | 1745.39 (1551.06,1971.27) | 410.51 (365.57,462.97) |  | 2740.65 (2328.69,3144.19) | 292.12 (249.33,335.41) |  | 1.46 (1.36,1.56) | -1.14 (-1.23,-1.04) |
| Republic of Mauritius | 1571.52 (1473.88,1688.32) | 202.39 (189.94,217.70) |  | 6429.38 (5889.36,6912.33) | 354.37 (324.38,380.39) |  | 4.11 (3.90,4.32) | 1.24 (1.02,1.45) |
| Republic of Moldova | 20548.50 (18384.42,22553.17) | 453.14 (406.19,495.87) |  | 26785.38 (22644.61,31410.39) | 450.95 (381.09,528.67) |  | 1.28 (0.86,1.71) | 0.44 (0.02,0.86) |
| Republic of Mozambique | 3900.07 (3243.07,4627.10) | 67.45 (56.35,79.08) |  | 9537.08 (7055.32,12391.55) | 86.42 (64.64,109.87) |  | 3.45 (3.27,3.63) | 1.29 (1.12,1.47) |
| Republic of Namibia | 978.71 (805.71,1154.50) | 138.97 (114.40,163.61) |  | 2389.94 (1676.67,3188.20) | 157.04 (113.73,204.68) |  | 2.70 (2.41,3.00) | 0.30 (0.03,0.57) |
| Republic of Nauru | 24.57 (13.74,34.84) | 464.87 (270.62,650.38) |  | 28.27 (16.48,38.63) | 428.75 (258.39,574.72) |  | 0.24 (0.15,0.33) | -0.33 (-0.37,-0.29) |
| Republic of Nicaragua | 1944.92 (1716.61,2203.95) | 111.39 (97.97,126.82) |  | 7557.88 (6073.97,9242.67) | 145.04 (116.78,176.92) |  | 4.84 (4.56,5.11) | 1.26 (1.00,1.52) |
| Republic of Niue | 5.85 (4.74,7.18) | 269.57 (215.35,332.67) |  | 5.99 (4.75,7.37) | 281.49 (224.77,345.69) |  | -0.21 (-0.32,-0.09) | -0.00 (-0.04,0.04) |
| Republic of Palau | 34.64 (26.46,45.75) | 344.14 (263.37,452.81) |  | 63.15 (47.67,79.82) | 290.20 (223.31,362.20) |  | 1.69 (1.57,1.82) | -0.43 (-0.52,-0.34) |
| Republic of Panama | 3165.38 (2905.29,3417.24) | 202.28 (185.48,218.65) |  | 12034.61 (9266.42,14754.78) | 272.38 (209.78,334.02) |  | 4.70 (4.59,4.81) | 1.23 (1.12,1.33) |
| Republic of Paraguay | 3345.88 (2855.58,3960.66) | 143.29 (122.55,169.78) |  | 14608.63 (11240.66,18662.97) | 243.96 (188.24,310.59) |  | 5.12 (4.91,5.34) | 1.99 (1.81,2.18) |
| Republic of Peru | 24656.83 (20678.85,28786.12) | 194.30 (162.75,227.38) |  | 71622.15 (53409.42,94503.85) | 210.60 (157.02,278.12) |  | 3.53 (3.34,3.72) | 0.19 (-0.01,0.38) |
| Republic of Poland | 219290.32 (210467.76,227392.53) | 501.91 (481.90,520.76) |  | 366683.18 (332272.90,397994.12) | 522.54 (474.13,567.42) |  | 1.66 (1.53,1.78) | 0.03 (-0.13,0.19) |
| Republic of Rwanda | 8879.14 (6865.62,11055.23) | 280.09 (218.41,346.77) |  | 14867.50 (10266.26,20524.82) | 216.77 (151.91,293.35) |  | 1.26 (0.74,1.79) | -1.60 (-1.93,-1.27) |
| Republic of San Marino | 170.52 (141.93,203.56) | 488.78 (407.32,586.46) |  | 172.80 (114.57,239.85) | 239.27 (152.40,336.44) |  | 0.95 (0.55,1.35) | -1.41 (-1.75,-1.08) |
| Republic of Senegal | 4311.28 (3535.60,5210.95) | 127.66 (105.75,153.72) |  | 12119.72 (9114.36,16097.75) | 150.77 (114.40,198.68) |  | 3.63 (3.47,3.79) | 0.71 (0.55,0.87) |
| Republic of Serbia | 61318.32 (51615.74,74366.29) | 557.06 (470.88,679.03) |  | 77936.14 (62827.43,94855.02) | 484.60 (388.90,590.27) |  | 0.61 (0.42,0.80) | -0.53 (-0.66,-0.41) |
| Republic of Seychelles | 232.82 (208.02,263.78) | 408.47 (364.49,462.04) |  | 561.02 (481.02,643.68) | 469.22 (403.01,539.66) |  | 3.07 (2.90,3.25) | 0.57 (0.34,0.81) |
| Republic of Sierra Leone | 2128.50 (1612.93,2776.39) | 101.40 (77.24,131.66) |  | 4828.68 (3485.33,6572.92) | 119.43 (87.67,161.53) |  | 3.07 (2.78,3.35) | 0.89 (0.75,1.04) |
| Republic of Singapore | 12101.70 (11051.04,13321.36) | 516.91 (470.83,569.02) |  | 23337.62 (20591.10,26390.87) | 272.39 (239.77,309.64) |  | 2.10 (1.84,2.36) | -2.18 (-2.40,-1.97) |
| Republic of Slovenia | 12146.95 (10728.83,13600.93) | 493.06 (435.07,553.73) |  | 14623.82 (11843.05,17889.57) | 337.40 (273.75,416.54) |  | 0.46 (0.17,0.75) | -1.44 (-1.75,-1.14) |
| Republic of South Africa | 46361.00 (40722.94,56803.46) | 204.54 (178.73,256.10) |  | 127809.56 (114215.31,144690.38) | 262.56 (235.23,295.79) |  | 3.47 (3.22,3.73) | 0.98 (0.73,1.23) |
| Republic of South Sudan | 6359.21 (4298.46,9103.78) | 234.88 (160.39,333.63) |  | 11887.60 (8211.61,15942.55) | 273.52 (191.51,363.38) |  | 2.17 (1.87,2.47) | 0.55 (0.40,0.70) |
| Republic of Sudan | 16973.72 (11031.17,24643.61) | 164.87 (110.80,237.22) |  | 40005.62 (25653.08,59276.15) | 174.14 (115.92,252.47) |  | 2.80 (2.68,2.93) | 0.27 (0.20,0.34) |
| Republic of Suriname | 800.23 (716.78,891.09) | 301.50 (270.59,334.17) |  | 2139.88 (1632.99,2686.93) | 333.10 (255.41,418.25) |  | 3.65 (3.43,3.88) | 0.59 (0.39,0.79) |
| Republic of Tajikistan | 6421.08 (5372.10,7649.50) | 212.56 (178.08,252.74) |  | 8357.00 (6175.21,11709.12) | 122.52 (92.92,159.66) |  | 0.73 (0.30,1.16) | -1.83 (-2.04,-1.62) |
| Republic of the Congo | 3075.19 (2137.85,3945.75) | 266.40 (192.81,339.67) |  | 7473.18 (5630.75,9750.58) | 242.56 (187.26,310.42) |  | 2.81 (2.59,3.03) | -0.42 (-0.55,-0.29) |
| Republic of the Gambia | 237.09 (180.89,296.00) | 62.65 (48.75,78.06) |  | 751.70 (556.52,995.22) | 70.75 (53.17,92.31) |  | 3.63 (3.43,3.84) | 0.28 (0.12,0.44) |
| Republic of the Marshall Islands | 52.86 (39.64,63.79) | 289.32 (223.09,350.28) |  | 119.63 (85.68,162.09) | 299.61 (218.73,393.72) |  | 2.77 (2.69,2.85) | 0.12 (0.05,0.20) |
| Republic of the Niger | 3137.22 (2403.76,4079.16) | 105.39 (80.68,135.35) |  | 9468.67 (6649.73,13039.95) | 110.28 (78.25,149.79) |  | 3.86 (3.71,4.00) | 0.34 (0.24,0.44) |
| Republic of the Philippines | 92925.32 (83551.02,105069.82) | 263.22 (235.65,305.88) |  | 311024.04 (259599.48,365688.28) | 342.33 (287.17,402.30) |  | 4.15 (4.08,4.22) | 1.02 (0.96,1.09) |
| Republic of the Union of Myanmar | 79091.02 (47983.19,115618.37) | 304.55 (190.71,441.54) |  | 147249.70 (107476.27,193002.15) | 286.48 (209.56,372.14) |  | 1.85 (1.77,1.93) | -0.33 (-0.42,-0.25) |
| Republic of Trinidad and Tobago | 2954.92 (2729.94,3188.29) | 348.82 (322.17,376.80) |  | 6801.21 (5131.39,8753.27) | 355.66 (268.26,457.15) |  | 2.73 (2.60,2.86) | 0.03 (-0.10,0.16) |
| Republic of Tunisia | 9230.06 (7640.21,11119.52) | 179.45 (148.22,215.01) |  | 23409.38 (16663.95,32389.89) | 173.62 (124.24,238.55) |  | 2.85 (2.77,2.94) | -0.27 (-0.34,-0.20) |
| Republic of Turkey | 156583.28 (129710.46,185945.91) | 414.18 (348.96,486.36) |  | 283677.34 (227589.37,349142.97) | 299.82 (240.50,368.43) |  | 1.84 (1.51,2.18) | -1.12 (-1.45,-0.79) |
| Republic of Uganda | 16375.05 (13010.50,20522.11) | 238.90 (193.27,296.41) |  | 42261.15 (31033.83,57923.02) | 252.54 (192.21,337.66) |  | 2.49 (2.25,2.74) | -0.39 (-0.65,-0.12) |
| Republic of Uzbekistan | 21805.07 (19467.89,24383.81) | 174.02 (153.92,194.76) |  | 39161.68 (31820.73,47761.39) | 131.07 (105.91,160.54) |  | 1.85 (1.32,2.38) | -0.91 (-1.35,-0.48) |
| Republic of Vanuatu | 176.80 (123.34,237.82) | 252.35 (181.52,331.03) |  | 490.40 (364.10,644.38) | 252.45 (192.41,326.02) |  | 3.22 (3.10,3.34) | -0.11 (-0.21,-0.01) |
| Republic of Yemen | 9291.86 (6104.43,13326.24) | 169.86 (116.25,237.83) |  | 27237.50 (17874.55,40480.89) | 169.25 (111.11,246.93) |  | 3.64 (3.54,3.74) | 0.05 (-0.03,0.12) |
| Republic of Zambia | 7860.10 (6376.20,9610.72) | 245.87 (201.25,299.31) |  | 26969.16 (16170.83,54833.21) | 329.22 (207.45,621.59) |  | 4.03 (3.76,4.30) | 0.88 (0.79,0.98) |
| Republic of Zimbabwe | 9981.49 (8257.17,12082.21) | 236.01 (195.70,281.78) |  | 26122.27 (19473.54,34413.02) | 333.74 (255.00,427.34) |  | 3.32 (2.80,3.83) | 1.53 (1.00,2.07) |
| Romania | 91287.25 (80293.81,102298.66) | 324.57 (285.45,364.51) |  | 177258.68 (150603.62,210784.46) | 504.85 (430.58,593.48) |  | 1.91 (1.68,2.15) | 1.17 (0.94,1.39) |
| Russian Federation | 790776.07 (765639.68,812614.24) | 433.85 (419.87,445.81) |  | 1029232.57 (944840.91,1113830.62) | 436.30 (400.45,472.48) |  | 0.53 (0.36,0.71) | -0.21 (-0.39,-0.03) |
| Saint Kitts and Nevis | 141.73 (127.14,156.00) | 389.84 (350.99,427.73) |  | 258.09 (213.50,311.18) | 370.59 (309.14,438.31) |  | 2.45 (2.10,2.79) | 0.28 (0.12,0.44) |
| Saint Lucia | 236.41 (217.61,255.67) | 272.09 (250.78,294.20) |  | 583.48 (475.80,712.19) | 244.76 (199.83,298.55) |  | 2.94 (2.74,3.13) | -0.55 (-0.73,-0.38) |
| Saint Vincent and the Grenadines | 187.47 (170.33,205.37) | 261.05 (237.25,286.53) |  | 424.29 (370.92,490.32) | 301.78 (264.09,348.32) |  | 2.80 (2.61,2.98) | 0.47 (0.34,0.60) |
| Slovak Republic | 39173.77 (34298.45,44929.87) | 655.35 (575.57,752.30) |  | 52942.60 (43605.12,63340.53) | 563.00 (463.95,671.00) |  | 1.01 (0.94,1.08) | -0.49 (-0.59,-0.39) |
| Socialist Republic of Viet Nam | 87550.49 (69187.84,110585.44) | 208.76 (165.74,262.44) |  | 315042.74 (238349.37,387372.35) | 299.88 (228.83,365.46) |  | 4.47 (4.39,4.55) | 1.38 (1.27,1.49) |
| Solomon Islands | 360.59 (192.17,531.55) | 235.62 (136.54,340.99) |  | 975.79 (674.84,1314.34) | 243.85 (172.74,326.51) |  | 3.20 (3.06,3.33) | 0.11 (-0.02,0.25) |
| State of Eritrea | 3544.58 (2718.02,4400.35) | 258.09 (200.39,313.35) |  | 9054.01 (6523.69,12425.89) | 282.31 (208.73,376.44) |  | 3.45 (3.30,3.61) | 0.31 (0.23,0.39) |
| State of Israel | 21555.52 (19242.74,23957.64) | 450.61 (405.59,500.48) |  | 32991.24 (27951.42,37851.36) | 270.88 (231.00,310.26) |  | 0.84 (0.51,1.17) | -2.12 (-2.43,-1.81) |
| State of Kuwait | 986.65 (879.77,1110.13) | 139.12 (125.13,154.43) |  | 6724.14 (5363.04,8444.60) | 194.76 (155.30,241.81) |  | 6.53 (6.13,6.93) | 1.40 (0.89,1.92) |
| State of Libya | 6539.17 (5121.16,8375.83) | 323.05 (252.82,415.47) |  | 19360.23 (14088.64,26536.92) | 332.66 (242.52,449.39) |  | 3.94 (3.80,4.08) | 0.42 (0.26,0.58) |
| State of Qatar | 422.90 (347.10,514.39) | 309.20 (256.48,374.01) |  | 2683.85 (1976.91,3710.33) | 232.78 (176.63,318.28) |  | 7.28 (6.81,7.75) | -0.65 (-1.26,-0.03) |
| Sultanate of Oman | 771.09 (552.20,1039.88) | 101.39 (74.22,133.64) |  | 1828.76 (1348.84,2527.19) | 81.01 (62.04,103.75) |  | 2.94 (2.83,3.05) | -0.35 (-0.62,-0.09) |
| Swiss Confederation | 33785.18 (30781.91,36628.60) | 335.34 (306.89,362.11) |  | 38831.31 (32791.62,44771.35) | 219.17 (188.16,250.76) |  | 0.36 (0.22,0.49) | -1.49 (-1.67,-1.31) |
| Syrian Arab Republic | 10291.30 (7969.61,12959.55) | 170.06 (133.52,211.58) |  | 20673.93 (14851.85,28738.64) | 150.89 (109.66,207.89) |  | 2.30 (2.10,2.49) | -0.53 (-0.71,-0.34) |
| Taiwan (Province of China) | 72603.08 (66569.21,78514.85) | 426.07 (390.68,460.46) |  | 205500.97 (180532.06,229553.12) | 503.50 (445.35,558.95) |  | 3.14 (2.89,3.40) | 0.21 (-0.05,0.47) |
| Togolese Republic | 1466.77 (1159.61,1885.17) | 109.37 (87.44,138.07) |  | 5755.65 (3784.61,8166.03) | 139.97 (92.93,195.14) |  | 4.68 (4.61,4.76) | 0.95 (0.87,1.02) |
| Tokelau | 3.51 (2.69,4.44) | 263.61 (202.02,331.93) |  | 3.52 (2.74,4.58) | 240.72 (187.63,314.76) |  | -0.16 (-0.26,-0.05) | -0.35 (-0.39,-0.31) |
| Turkmenistan | 3791.14 (3454.52,4151.33) | 175.71 (159.64,192.74) |  | 6275.44 (4731.34,8270.11) | 140.91 (106.84,184.79) |  | 1.68 (1.13,2.23) | -0.69 (-1.19,-0.19) |
| Tuvalu | 20.06 (15.47,25.77) | 285.36 (226.39,363.08) |  | 28.19 (22.33,36.16) | 263.31 (209.89,336.45) |  | 1.06 (0.99,1.13) | -0.28 (-0.31,-0.25) |
| Ukraine | 374269.82 (343286.88,405218.98) | 526.76 (484.33,569.18) |  | 298105.21 (217349.12,388052.03) | 397.57 (288.28,518.22) |  | -1.09 (-1.26,-0.92) | -1.21 (-1.37,-1.05) |
| Union of the Comoros | 489.94 (321.36,653.63) | 226.70 (153.79,300.40) |  | 1191.40 (846.19,1650.32) | 230.84 (164.12,314.31) |  | 2.76 (2.55,2.97) | -0.06 (-0.20,0.09) |
| United Arab Emirates | 2603.76 (1696.60,3550.32) | 426.71 (290.48,576.04) |  | 11268.13 (8022.84,16889.17) | 264.33 (200.81,376.61) |  | 5.51 (5.22,5.80) | -0.17 (-0.62,0.29) |
| United Kingdom of Great Britain and Northern Ireland | 466187.22 (448382.34,478204.31) | 535.50 (516.32,548.80) |  | 415463.62 (384297.42,434094.51) | 335.26 (314.03,348.83) |  | -0.38 (-0.54,-0.22) | -1.56 (-1.67,-1.45) |
| United Mexican States | 65192.51 (63132.16,67480.59) | 142.38 (137.66,147.36) |  | 292715.05 (257982.06,329408.11) | 223.11 (196.93,250.75) |  | 5.10 (4.97,5.22) | 1.55 (1.42,1.68) |
| United Republic of Tanzania | 24742.50 (19902.62,30880.15) | 211.51 (171.18,262.25) |  | 61623.14 (45053.27,81808.04) | 220.30 (162.36,289.53) |  | 2.94 (2.85,3.04) | 0.12 (0.07,0.18) |
| United States of America | 1461904.88 (1382093.16,1520864.74) | 469.35 (445.83,487.61) |  | 1692400.79 (1586159.48,1776257.87) | 315.59 (298.11,330.02) |  | 0.41 (0.35,0.48) | -1.40 (-1.46,-1.33) |
| United States Virgin Islands | 418.71 (354.08,494.43) | 481.68 (408.44,566.22) |  | 528.37 (404.63,677.64) | 330.77 (254.83,422.05) |  | 0.97 (0.61,1.34) | -1.01 (-1.22,-0.80) |

Abbreviations: ASDR, age-standardized DALYs rate; EAPC, estimated annual percentage change; SDI, Sociodemographic Index; UI, uncertainty interval.
